# Corrigendum to: Bmal1 is involved in the regulation of macrophage cholesterol homeostasis

**DOI:** 10.1172/jci.insight.205164

**Published:** 2026-03-09

**Authors:** Xiaoyue Pan, John O’Hare, Cyrus Mowdawalla, Samantha Mota, Nan Wang, M. Mahmood Hussain

Original citation: *JCI Insight*. 2025;10(21):e194304. https://doi.org/10.1172/jci.insight.194304

Citation for this corrigendum: *JCI Insight*. 2026;11(5):e205164. https://doi.org/10.1172/jci.insight.205164

During final revision of the manuscript, several text changes and 3 references were inadvertently omitted. The HTML and PDF versions have been updated online. The *Journal* has also published an online version of the article that highlights the changes that have been made (Supplemental File, Marked).

The authors and the *Journal* regret the error.

## Version 1 05/05/2026

Udpated date

