# Supplementary material for: Bmal1 is involved in the regulation of macrophage cholesterol homeostasis
Source: JCI Insight. 2025 Sep 30;10(21):e194304. doi: 10.1172/jci.insight.194304 (PMC12643489; doi:10.1172/jci.insight.194304)
Supplement: Supplemental data [file jciinsight-10-194304-s008.pdf]

Sup Figure 1

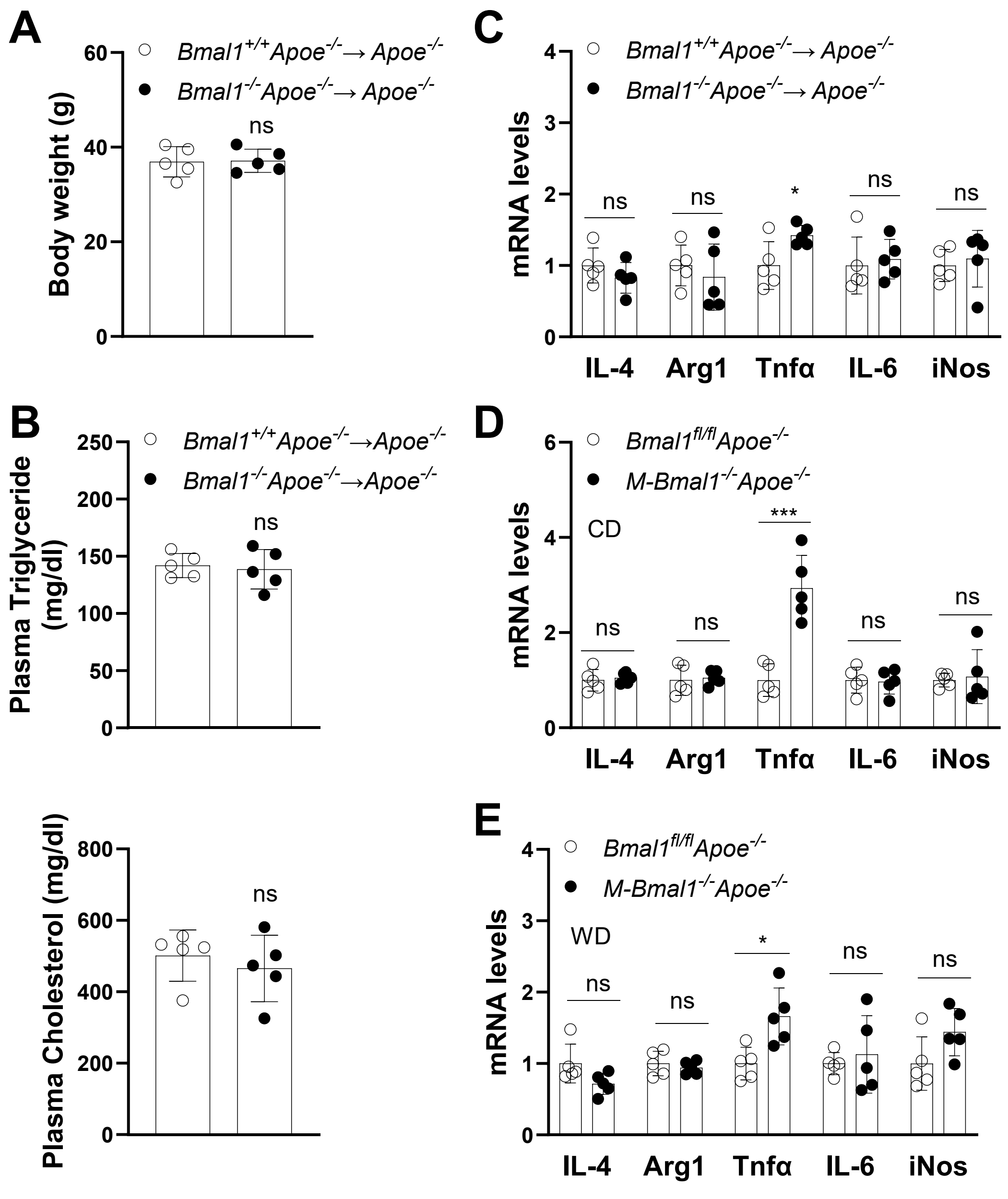

Supplementary Fig 1. No changes in plasma lipids in *Apoe*$^{-/-}$ mice transplanted with bone marrow cells derived from *Bmal1*$^{-/-}$*Apoe*$^{-/-}$ mice.

(**A**) Body weights and (**B**) plasma triglycerides and cholesterol were measured in the mice described in **Fig 1A**. Values are represented as mean ± SD, n=5. * P < 0.05, ** P < 0.01; *** P < 0.001, compared with control, unpair t-tests. (**C-E**) Mϕs from different mice in Fig 1A-C were used to measure (n=5) mRNA levels of M1 or M2 macrophage-associated genes. Mean ± SD, n=5, * P < 0.05, ** P < 0.01; *** P < 0.001, compared with control, multiple t-tests followed by Holm-Šídák method .

Sup Figure 2

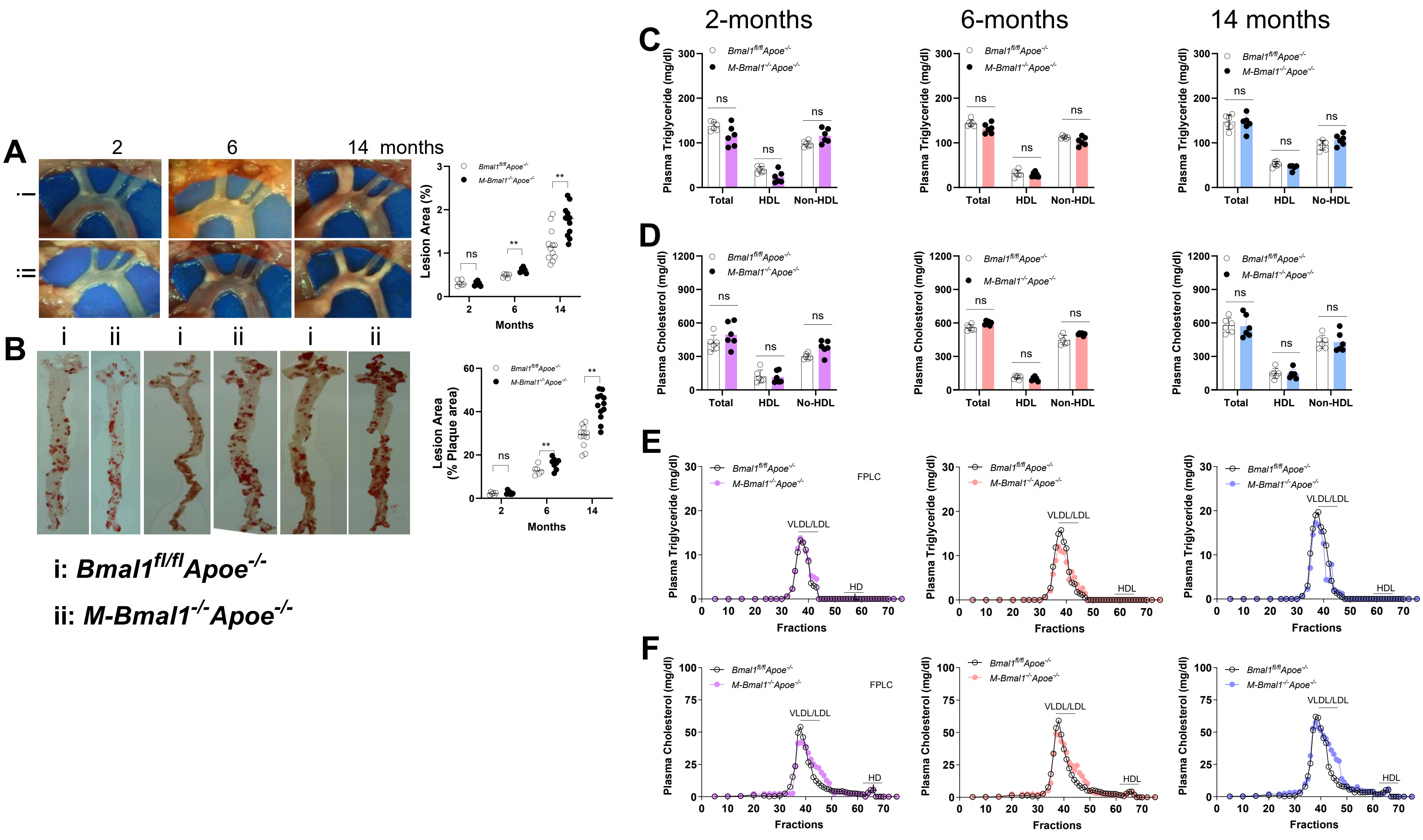

i: ***Bmal1$^{fl/fl}$Apoe$^{-/-}$***

ii: ***M-Bmal1$^{-/-}$Apoe$^{-/-}$***

Supplementary Fig 2. Age dependent changes in lipids and lipoproteins, and atherosclerosis in *M-Bmal1$^{-/-}$Apoe$^{-/-}$* mice.

(A) Male *Bmal1$^{fl/fl}$Apoe$^{-/-}$* and *M-Bmal1$^{-/-}$Apoe$^{-/-}$* mice were fed a chow diet for 2, 6 or 14 months, then dissected to visualize atherosclerotic lesions at the aortic arch and photographed (left). Plaque areas were quantified and graphed (right). Scale bar, 2 mm. Mean ± SD, n=12–15, * $P < 0.05$, ** $P < 0.01$, *** $P < 0.001$ compared with control *Bmal1$^{fl/fl}$Apoe$^{-/-}$* mice, multiple t-test followed by Holm-Šídák method.

(**B**) Whole aortas were stained with Oil Red O (left) and quantified (right). Scale bar, 5 mm. Mean ± SD, n=12–15, * $P < 0.05$, ** $P < 0.01$, *** $P < 0.001$ compared with control *Bmal1$^{fl/fl}$Apoe$^{-/-}$* mice, multiple t-test followed by Holm-Šídák method.

(**C-D**) Triglycerides (C) and cholesterol (D) were measured in total plasma, HDL or non-HDL fractions isolated from *M-Bmal1$^{-/-}$Apoe$^{-/-}$* or *Bmal1$^{fl/fl}$Apoe$^{-/-}$* mice. Mean ± SD, n=12–15, * $P < 0.05$, ** $P < 0.01$, *** $P < 0.001$ compared with control Bmal1$^{fl/fl}$Apoe$^{-/-}$ mice, multiple t-test followed by Holm-Šídák method. (E-F) Plasma was subjected to FPLC, and cholesterol and triglycerides were measured in various fractions.

Sup Figure 3

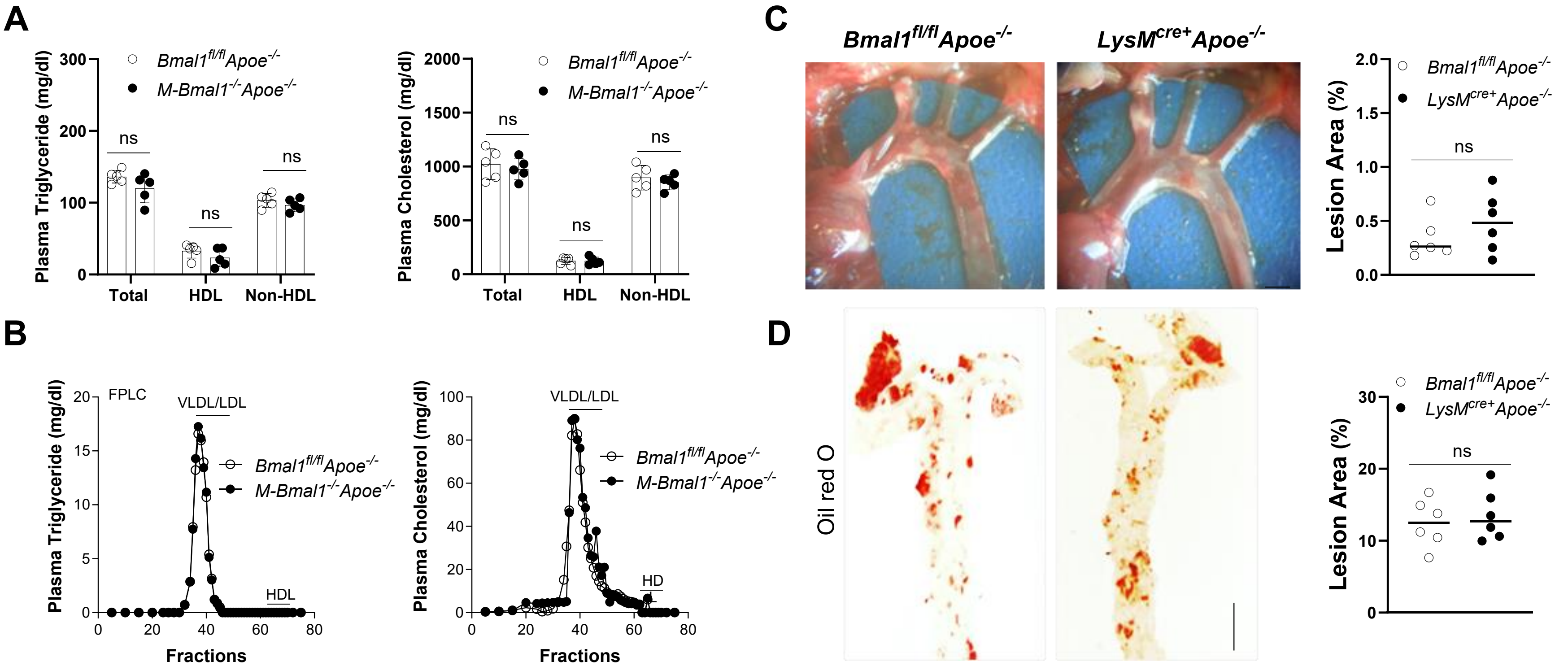

Supplementary Fig 3. No differences in plasma lipids and lipoproteins between WD-fed *M-Bmal1*$^{-/-}$*Apoe*$^{-/-}$ and control mice.

(**A**) Triglyceride and cholesterol levels were measured in total plasma, HDL or non-HDL fractions of *M-Bmal1*$^{-/-}$*Apoe*$^{-/-}$ and *Bmal1*$^{fl/fl}$*Apoe*$^{-/-}$ WD-fed mice. Mean ± SD, n=5-6, multiple t-test followed by Holm-Šídák method.

(**B**) Cholesterol and triglycerides were measured in lipoproteins after FPLC separation.

(**C-D**) Male *Bmal1*$^{fl/fl}$*Apoe*$^{-/-}$ and *LysM*$^{cre+}$*Apoe*$^{-/-}$ mice were fed a chow diet for 7-8 months, then dissected to visualize atherosclerotic lesions at the aortic arch and photographed (**C** left). Plaque areas were quantified and graphed (**C** right). Scale bar, 2 mm. Whole aortas were stained with Oil Red O (**D** left) and quantified (**D** right). Scale bar, 5 mm. Mean ± SD, n=6, unpaired t-test.

Sup Figure 4

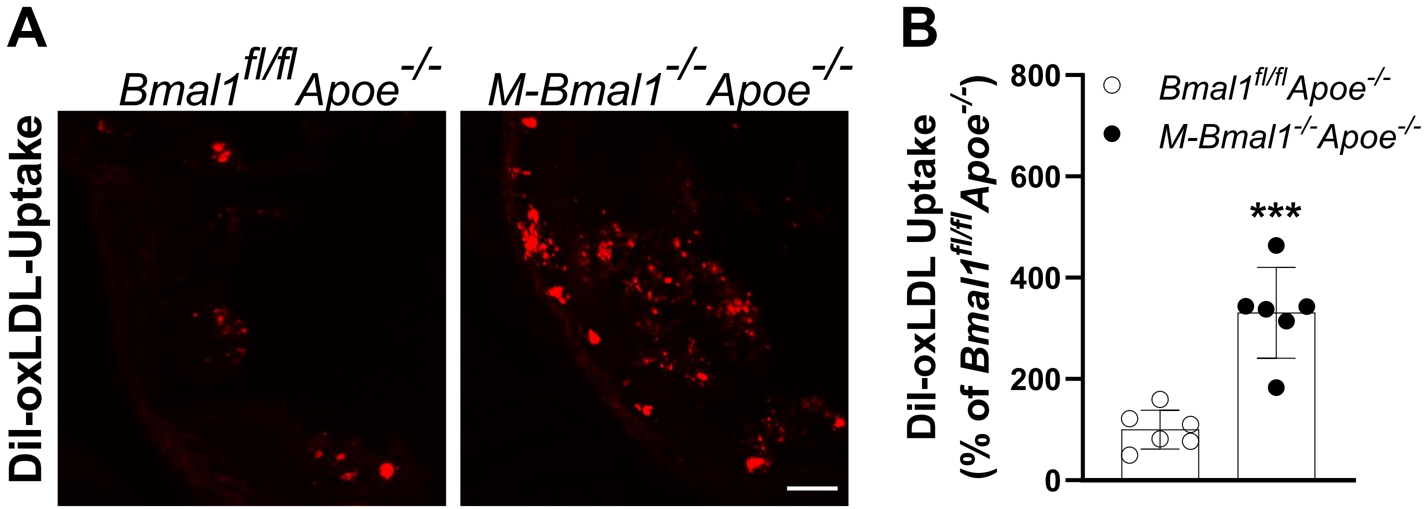

Supplementary Fig 4. Mϕ Bmal1 deficiency increases aortic oxLDL uptake. **(A-B)** 7-8 months male M-*Bmal1*$^{-/-}$*Apoe*$^{-/-}$ *or Bmal1*$^{fl/fl}$*Apoe*$^{-/-}$ mice were injected with DiI-labeled oxLDL (30 µg protein/50 µL) at ZT 12. After 18 h, sections from cardiac/aortic junctions were visualized under a fluorescence microscope, photographed and quantified; scale, 200 µm. Mean ± SD, n=6, *** P < 0.001 compared with control *Bmal1*$^{fl/fl}$ *Apoe*$^{-/-}$ mice, unpaired t-test.

Sup Figure 5

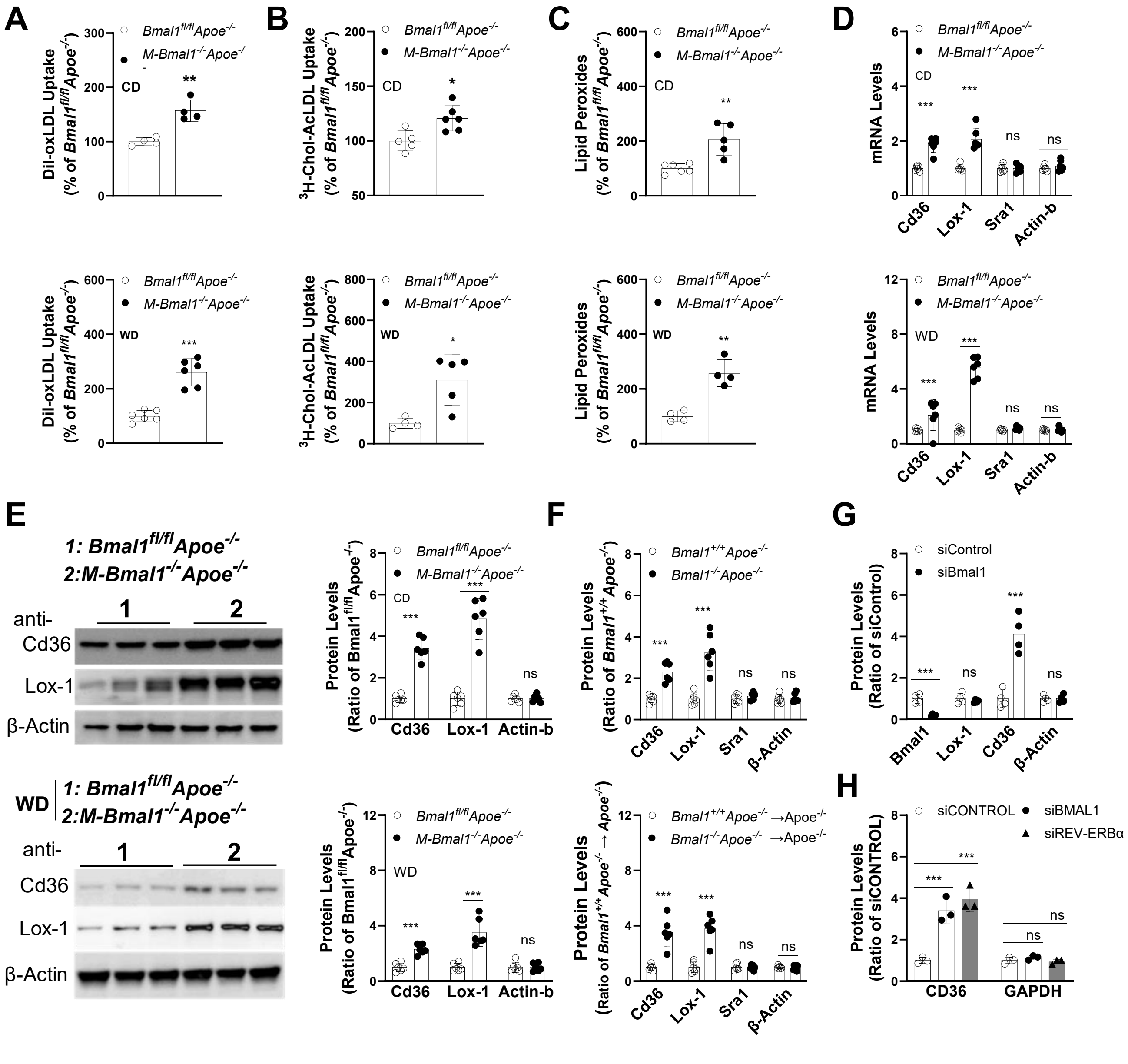

Supplementary Fig 5. Bmal1 deficiency increases ox-LDL uptake and Cd36 expression in CD or WD fed Bmal1 deficient Mϕs.

(**A-B**) BMDMs from different mice were used to measure uptake of DiI-oxLDL (A) and ^{3}H-cholesterol ac-LDL (B). Mean ± SD, n=4–6, * P < 0.05, ** P < 0.01, *** P < 0.001 compared with control, unpaired t-test,

(**C**) Lipid peroxides were measured in BMDMs isolated from various mouse models. Mean ± SD, n=4–6, * P < 0.05, ** P < 0.01, *** P < 0.001 compared with control, unpaired t-test,

(**D-E**) mRNA (D) and protein (E, left: images, right:) levels of various scavenger receptors, measured in BMDMs from different Bmal1 deficient mice. Mean ± SD, n=4–6, * P < 0.05, ** P < 0.01, *** P < 0.001 compared with control, multiple t-test followed by Holm-Šídák method.

.

(**F-H**) Quantifications of protein bands shown in Figure 2. Protein bands (shown in Figure 2 as representative images) were quantified using ImageJ and normalized to control proteins. The panel numbers (G, H and O) correspond to those in Figure 2. (F) BMDMs from the indicated mouse models were used to measure protein (Figure 2G) levels (n=4–6). Mean ± SD, n=4–6, * P < 0.05, ** P < 0.01, *** P < 0.001 compared with control, unpaired t-test, or multiple t-test followed by Holm-Šídák method.

(G) WT BMDMs were transfected with siControl or siBmal1 for 12 hours. After 48 h, protein levels were measured. Mean ± SD, n=4–6, * P < 0.05, ** P < 0.01, *** P < 0.001 compared with control, unpaired t-test, or multiple t-test followed by Holm-Šídák method.

(H) Human PBMCs (2.0×10^{6}) were differentiated into Mϕs. Cells were then transfected with the indicated siRNAs for 48 h and subjected to gene protein (Figure 2O Down) studies. Mean ± SD, n=4–6, * P < 0.05, ** P < 0.01, *** P < 0.001 compared with control, multiple t-test followed by Holm-Šídák method.

Sup Figure 6

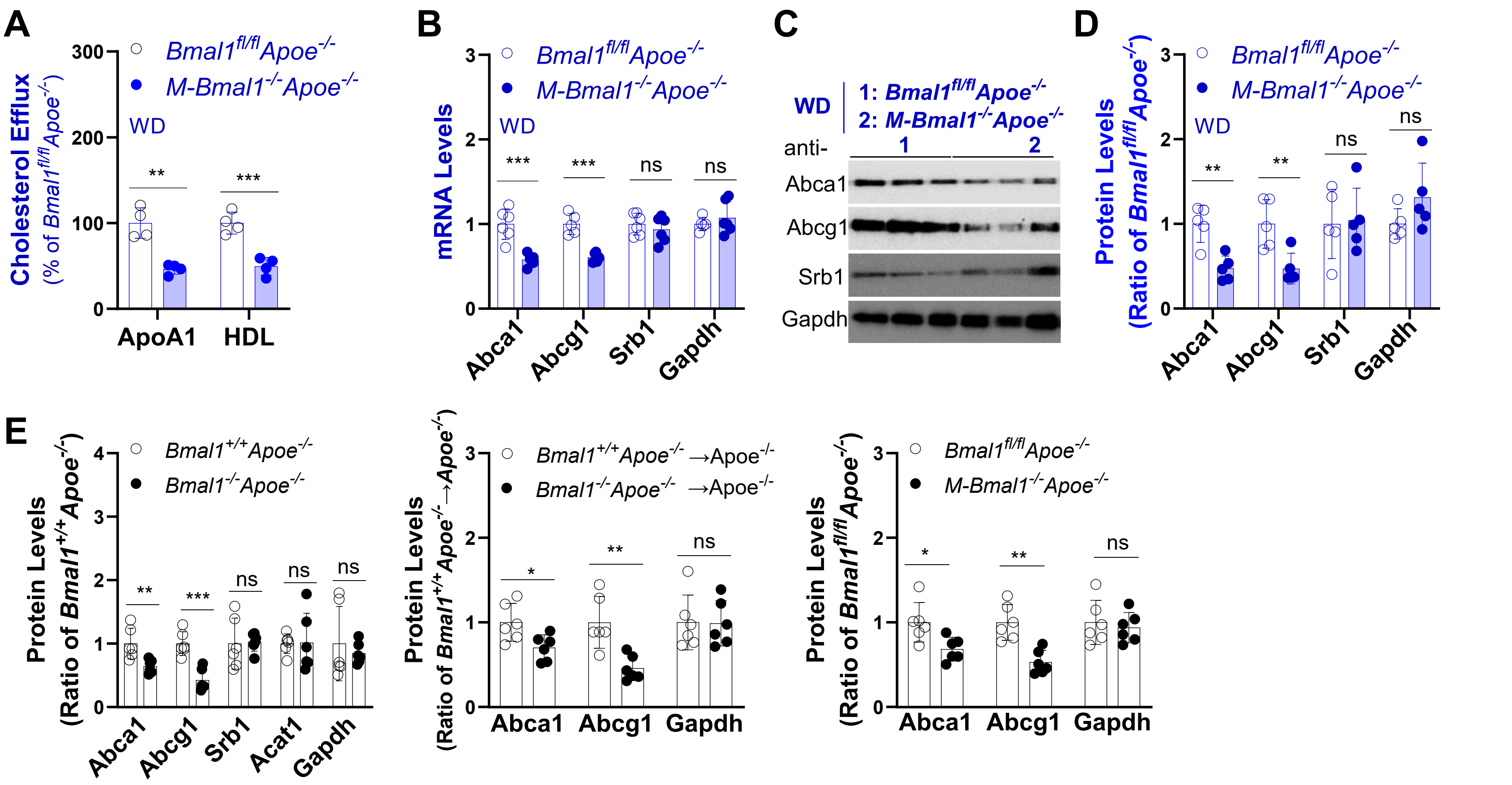

Supplementary Fig 6. Reduced cholesterol efflux and expression of Abca1/Abcg1 in WD fed Bmal1 deficient Mϕs.
(**A**) BMDMs from the indicated Bmal1 deficient mice were labeled with radiolabeled cholesterol, washed, and used to determine cholesterol efflux to the extracellular acceptors apoA1 and HDL. Mean ± SD, n=4, * $P < 0.05$, ** $P < 0.01$, *** $P < 0.001$ compared with control Bmal1$^{fl/fl}$ Apoe$^{-/-}$ mice, multiple t-test followed by Holm-Šídák method.

(**B-C**) BMDMs from different mice were used to measure mRNA (B) and protein (C-D) levels of cholesterol efflux transporters.
(**E**) Protein bands (shown in Figure 3E as representative images) were quantified using ImageJ and normalized to control proteins. Mϕs from different mice were used to measure (n=4-6) protein (Figure 3E) levels of cholesterol efflux-associated genes.

Mean ± SD, n=4–6, * $P < 0.05$, ** $P < 0.01$, *** $P < 0.001$ compared with control Bmal1$^{fl/fl}$ Apoe$^{-/-}$ mice, multiple t-test followed by Holm-Šídák method.

Sup Figure 7

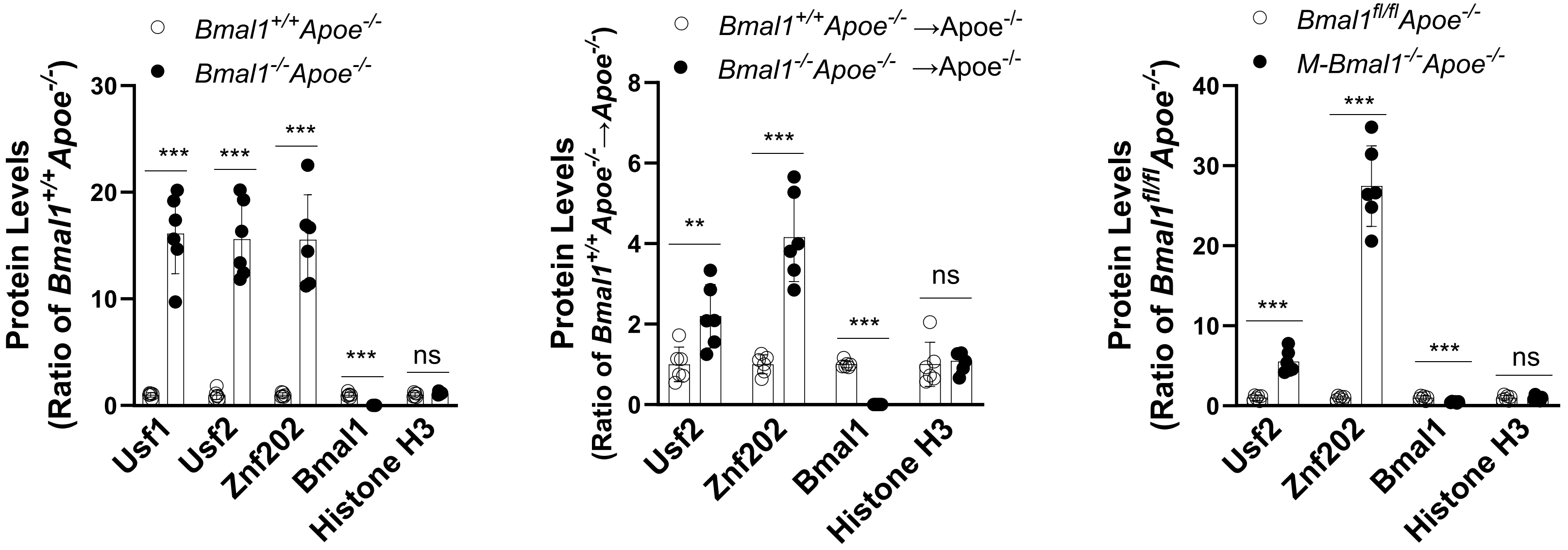

Supplementary Figure 7: Quantifications of protein bands shown in Figure 4. Protein bands (shown in Figure 4B as representative images) were quantified using ImageJ and normalized to control proteins. Mφs from various mouse models were used to measure protein (Figure 4B) levels of cholesterol efflux transport-associated TFs.
Mean ± SD, n=6, * P < 0.05, ** P < 0.01, *** P < 0.001 compared with control mice, multiple t-test followed by Holm-Šídák method.

Sup Figure 8

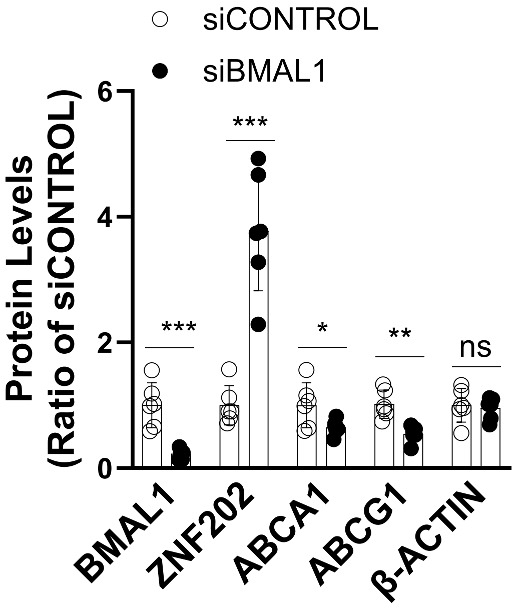

Supplementary Figure 8: Quantifications of protein bands shown in Figure 5. Protein bands (shown in Figure 5C as representative images) were quantified using ImageJ and normalized to control proteins. Human PBMCs were treated with siCONTROL or siBMAL1. After 48 h, the protein (Figure 5C) levels of ABCA1, ABCG1 and ZNF202 were quantified. siBmal1 increases Znf202 expression.

Mean ± SD, n=6, * $P < 0.05$, ** $P < 0.01$, *** $P < 0.001$ compared with control, multiple t-test followed by Holm-Šídák method.

.

Sup Figure 9

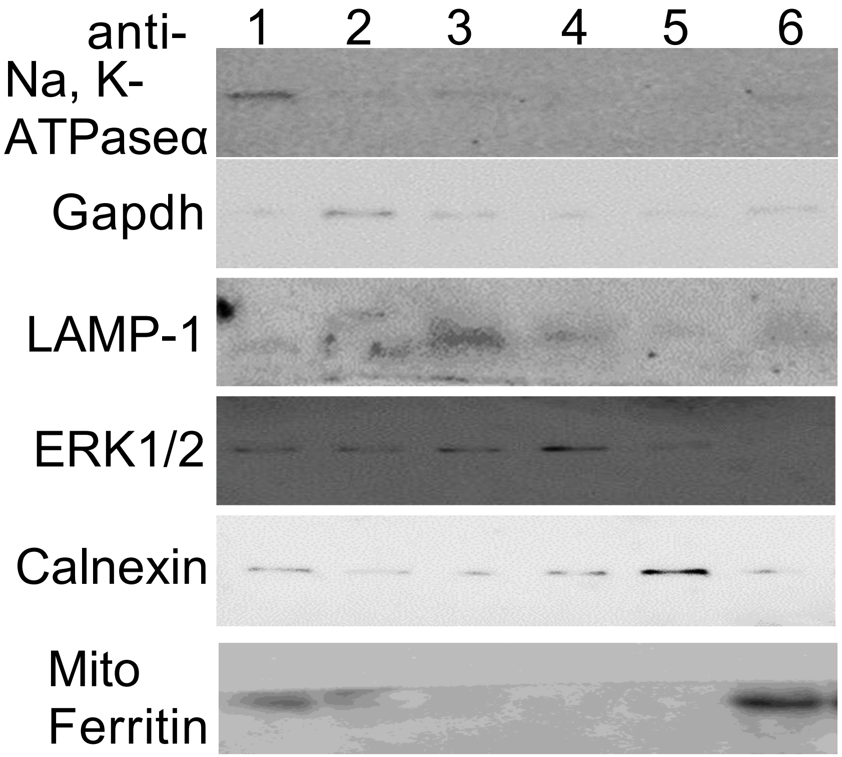

1: Plasma membrane (PM)
2: Cytosol
3: Lysosomes (Lyso)
4: Endsomes (Endo)
5: Endoplasmic reticulum (ER)
6: Mitochondria (Mito)

Supplementary Fig 9. Separation of subcellular organelles.
Subcellular organelles were purified as previously described [57, 68]. The purity of the organelles was determined by measurement of marker proteins via immunoblotting.

Sup Figure 10

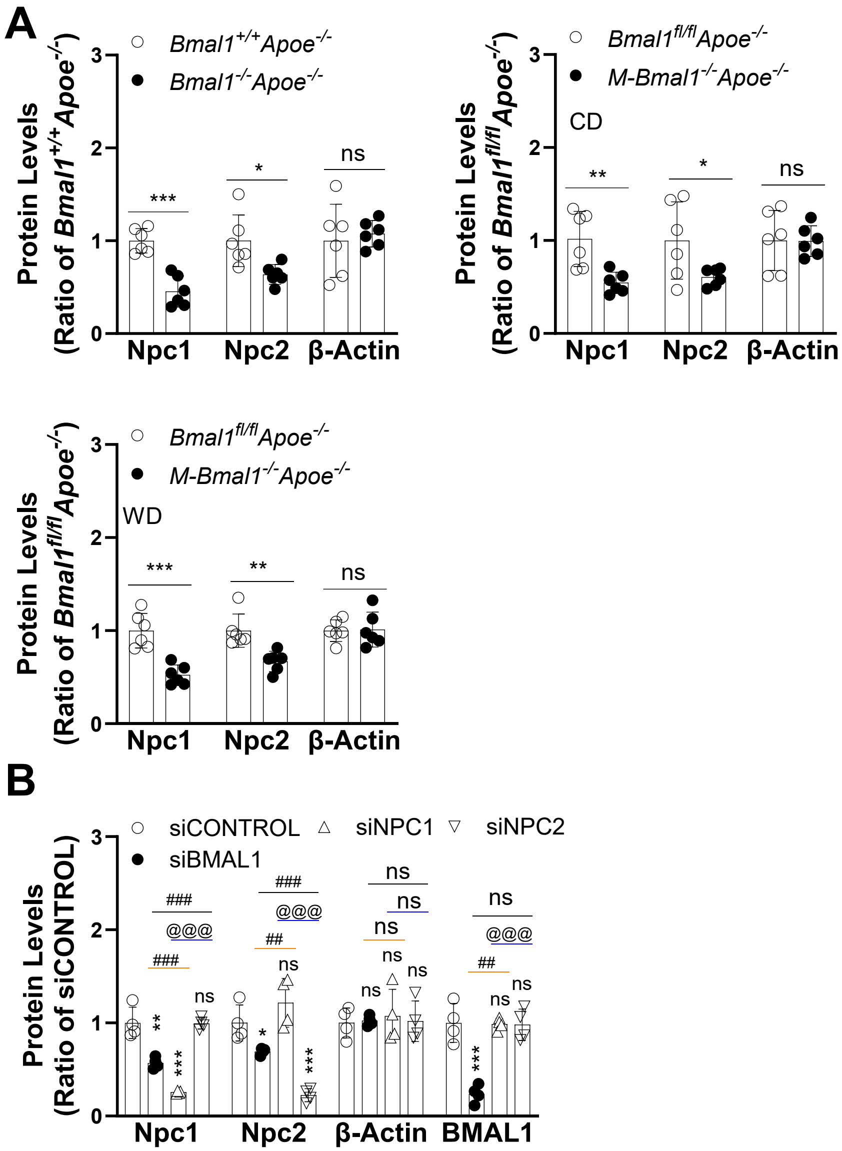

Supplementary Figure 10: Quantifications of protein bands shown in Figure 6. Protein bands (shown in Figure 6 G and H as representative images) were quantified using ImageJ and normalized to control proteins, as shown in (A) and (B).

(A) BMDMs from various mouse models were used to measure protein (Figure 6G) levels of Npc1 and Npc2. Mean ± SD, n=6, * P < 0.05, ** P < 0.01, *** P < 0.001 compared with control, multiple t-test followed by Holm-Šídák method.

(B) Mϕs derived from human PBMCs (2.0×10^{6}) were transfected with the indicated siRNAs for 48 h and used to measure protein (Figure 6H bottom). We replaced them with four for western blotting.

Mean ± SD, n=6, * P < 0.05, ** P < 0.01, *** P < 0.001 compared with control, multiple t-test. # P < 0.05, ## P < 0.01, ### P < 0.001 compared with siBMAL1, unpair t-test. @ P < 0.05, @@ P < 0.01, @@@ P < 0.001 compared with siNPC1, 1-way ANOVA with by Tukey's test multiple comparisons tests.

Sup Figure 11

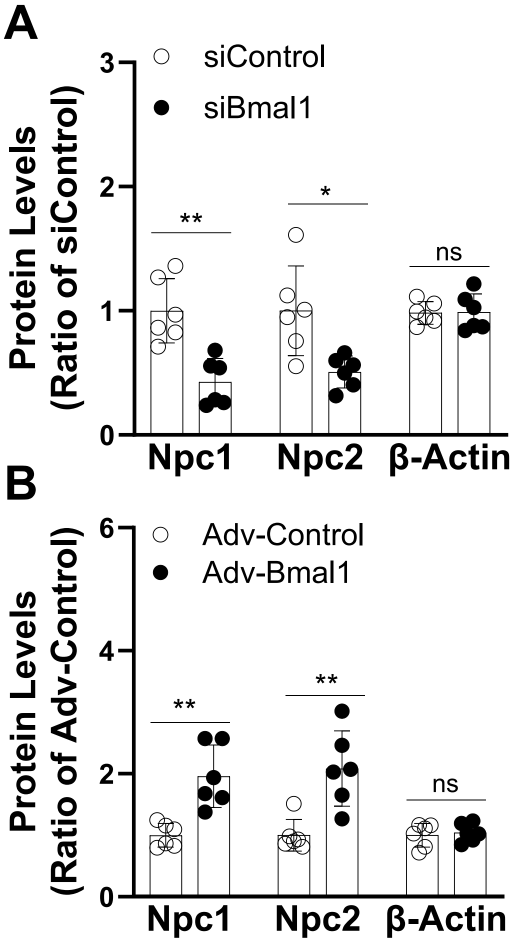

Supplementary Figure 11: Quantifications of protein bands shown in Figure 7. Protein bands (shown in Figure 7 B & D as representative images) were quantified using ImageJ and normalized to control proteins, as shown in (A) and (B).

(**A**) J774A.1 Mϕs were treated with various siRNAs, and mRNA and protein levels were measured after 48 h (Figure 7B).

(**B**) J774A.1 Mϕs were transduced with Adv-Control or Adv-Bmal1. After 48 h, Mϕs were used to measure protein levels (Figure 7D).

Mean ± SD, n=6, * P <0.05, ** P <0.01 and *** P <0.001 compared with Adv-Bmal1, multiple t-test followed by Holm-Šídák method.
