# Supplementary material for: Bmal1 is involved in the regulation of macrophage cholesterol homeostasis
Source: JCI Insight. 2025 Sep 30;10(21):e194304. doi: 10.1172/jci.insight.194304 (PMC12643489; doi:10.1172/jci.insight.194304)
Supplement: undefined [file jciinsight-10-194304-s009.pdf]

JCI insight

Bmal1 is involved in the regulation of macrophage cholesterol homeostasis

Xiaoyue Pan,[1,2,3] John O'Hare,[3] Cyrus Mowdawalla,[3] Samantha Mota,[1,3] Nan Wang,[4] and M. Mahmood Hussain[1,2,3,5]

[1]Department of Foundations of Medicine, NYU Grossman Long Island School of Medicine, Mineola, New York, USA. [2]Department of Cell Biology, SUNY Downstate Medical center, Brooklyn, New York, USA. [3]Diabetes and Obesity Research Center, NYU Langone Hospital–Long Island, Mineola, New York, USA. [4]Department of Medical Sciences, Columbia University, New York, New York, USA. [5]VA New York Harbor Healthcare System, Brooklyn, New York, USA.

Atherosclerotic cardiovascular disease is a major contributor to the global disease burden. We previously demonstrated that Clock-mutant mice, and mice with global or liver-specific Bmal1 deficiency, exhibit enhanced atherosclerosis because of overproduction of lipoproteins and sustained hyperlipidemia. Atherosclerosis initiation depends on cholesterol accumulation in subendothelial macrophages (Mφs). To clarify the role of Bmal1 in Mφ function and atherosclerosis, we used several global and myeloid-specific Bmal1-deficient mouse models. Mφ-specific Bmal1-deficient mice had similar plasma lipid levels, higher Mφ cholesterol content, and displayed greater atherosclerosis compared with controls. We attempted to understand molecular mechanisms for increased cellular cholesterol levels. Bmal1-deficient Mφs exhibited: (a) elevated expression of Cd36 and uptake of oxLDL; (b) diminished expression of Abca1 and Abcg1, and decreased cholesterol efflux and reverse cholesterol transport; and (c) reduced Npc1 and Npc2 expression and diminished cholesterol egress from lysosomes. Molecular studies revealed that Bmal1 directly regulates basal and cyclic expression of Npc1 and Npc2 by binding the E-box motif (CANNTG) sequence recognized by Bmal1 in their promoters and indirectly regulates the basal and temporal regulation of Cd36 and Abca1/Abcg1 involving Rev-erbα and Znf202 repressors, respectively. In conclusion, Mφ Bmal1 is a key regulator of the uptake of modified lipoproteins, cholesterol efflux, lysosomal cholesterol egress, and atherosclerosis and, therefore, may be a master regulator of cholesterol metabolism in Mφs. Restoration of Mφ Bmal1 expression or blocking of factors that decrease its activity may be effective in preventing atherosclerosis.

Introduction

Atherosclerosis poses a major human health burden worldwide and remains the major cause of death globally (1). This chronic disease is associated with lipid accumulation in large blood vessels and blood flow occlusion (2). Dysregulation of cholesterol metabolism and the inflammatory response are primary causal actors of atherosclerosis. Although several cell types are involved in etiology and progression, macrophages (Mφs) play a central role in the pathogenesis of atherosclerosis (3–5). Deregulation of cholesterol metabolism in Mφs is critical in the initiation of this disease (2, 6). Thus, studying Mφ cholesterol metabolism and atherosclerosis is critical.

Early atherosclerotic lesions are typified by the accumulation of cholesterol loaded Mφs in the arterial wall. Cholesterol levels increase in Mφs, through elevated uptake of modified lipoproteins by several scavenger receptors: Cd36, Sr-A, and Lox-1 (3, 6, 7). The modified lipoproteins are subsequently delivered to lysosomes, where cholesteryl esters are hydrolyzed to free cholesterol and fatty acids. Free cholesterol then undergoes egress to other cellular compartments, primarily the endoplasmic reticulum and plasma membrane. Two proteins, Niemann-Pick disease C1 (NPC1) and NPC2, play important roles in free cholesterol egress from lysosomes (8–10). From the plasma membrane, cholesterol efflux to the extracellular acceptors apoA1 and HDL occurs (11). In Mφs, cholesterol efflux is facilitated by the ATP binding cassette family A protein 1 (Abca1) and Abcg1 transporters (12–16). Abca1, which primarily delivers free cholesterol to lipid-free apoA1, is the most efficient transporter and is responsible for > 50% of Mφ cholesterol efflux (17). In contrast, Abcg1 enhances cholesterol efflux to high-density lipoprotein (HDL) (18, 19).

Conflict of interest: The authors have declared no conflict of interest exists.

Copyright: © 2025, Pan et al. This is an open access article published under the terms of the Creative Commons Attribution 4.0 International License.

Submitted: April 7, 2025
Accepted: September 23, 2025
Published: September 30, 2025

Reference information: *JCI Insight*. 2025;10(21):e194304. https://doi.org/10.1172/jci.insight.194304.

Circadian rhythms recur in intervals of approximately 24 hours. The circadian clock arises from autoregulatory transcriptional, translational, and posttranslational feedback loops of several transcription factors (TFs) encoded by "clock genes," including the circadian locomotor output cycles kaput (Clock), brain and muscle aryl hydrocarbon receptor nuclear translocator-like 1 (Arntl1 or Bmal1), or period and cryptochrome genes (20, 21). Bmal1:Clock heterodimers bind E-box sequences in the promoters of the *Per1/2/3* and *Cry1/2* genes and enhance their expression (22). When the cellular concentrations of Per and Cry increase, these proteins form complexes that suppress *Bmal1* transcription, thereby forming a negative feedback loop (23). This transcriptional autoregulatory loop is further modulated by several posttranslational modifications, such as phosphorylation and acetylation (24–26). In addition, Bmal1 expression is regulated by retinoic acid receptor–related orphan receptor α (Rorα), peroxisome proliferator-activated receptor γ coactivator 1-α (PGC1α) and reverse erythroblastosis virus α (Rev-erbα). Bmal1 expression is increased by Rorα and PGC1α, but it is suppressed by Rev-erbα, thus constituting a second regulatory loop (24, 25). Beyond this circuitry, circadian signals are transmitted to other genes via additional TFs called clock-controlled genes, which have intermediary roles in modulating metabolic pathways and physiological functions. Rhythmic changes in gene expression can be induced in cultured cells after brief exposure to high serum concentrations (serum shock) (27, 28).

Several biochemical, physiological, and behavioral processes exhibit circadian rhythms. Moreover, several diseases — e.g., sudden cardiac death, stroke, and heart attack — occur predominantly at certain times of the day. Clock genes are the main drivers of cyclic changes in biochemical pathways, thus preparing organisms for regular environmental changes. The importance of circadian mechanisms in humans is underscored by the observation of a strong correlation between the master molecular clock and the regulation of cardiovascular physiology (29, 30). *CLOCK* polymorphisms are associated with metabolic syndrome and obesity (31–34), whereas *BMAL1* polymorphisms are associated with susceptibility to hypertension and type 2 diabetes (35). Furthermore, night-shift workers have elevated risks of obesity and metabolic syndrome (36, 37). In addition, frequent travel across time zones or night-shift work increases cardiovascular disease risk (38–41).

Bmal1 is a key nonredundant enhancer of transcription generating molecular circadian rhythms (42, 43). Bmal1 normally interacts with Clock and subsequently increases target gene transcription. We have shown that Mφs from Clock mutant (*Clk*$^{\Delta19/\Delta19}$) mice, compared with WT mice, have defective cholesterol efflux and lower Abca1 expression (44). We have observed that the elevated atherosclerosis in clock mutant mice is due to diminished Mφ cholesterol efflux and reverse cholesterol transport (RCT). We have also reported that global and liver-specific Bmal1-deficient mice are prone to atherosclerosis and show diminished hepatic cholesterol efflux into bile (45). Others have also shown that global Bmal1 deficiency increases atherosclerosis in Ldlr KOK mice (46). The role of myeloid specific Bmal1 deficiency (*M-Bmal1*$^{-/-}$) in atherosclerosis has been examined by two groups reaching opposite conclusions (47, 48). Huo et al. have observed that *M-Bmal1*$^{-/-}$*Apoe*$^{-/-}$ mice have larger atherosclerotic lesions with more inflammatory resident Mφs and infiltrating Ly6c^{hi} monocytes than *Apoe*$^{-/-}$ mice (47). In contrast, Yang et al. have shown that *M-Bmal1*$^{-/-}$*Ldlr*$^{-/-}$ mice develop less atherosclerosis than *LysM*^{Cre}*Ldlr*$^{-/-}$ (48). Major differences between these studies included the mouse genetic backgrounds (*Apoe*$^{-/-}$ or *Ldlr*$^{-/-}$) and controls (*Apoe*$^{-/-}$ or *LysM*^{Cre}*Ldlr*$^{-/-}$). Here, using multiple *Apoe*$^{-/-}$ mouse models of atherosclerosis, we show that Mφ Bmal1 plays an important role in atherosclerosis. Biochemical studies indicate that Bmal1 regulates at least 3 critical pathways controlling cellular cholesterol homeostasis. Bmal1 regulates (a) Cd36 expression, thus controlling the uptake of modified lipoproteins; (b) Npc1 and Npc2, thus modulating cholesterol egress from lysosomes; and (c) Abca1 and Abcg1 expression, thus regulating cholesterol efflux and RCT. Hence, our findings suggest that Mφ Bmal1 is an atheroprotective master regulator of cholesterol metabolism.

Results

Mφ Bmal1 deficiency enhances atherosclerosis. We previously demonstrated that global and hepatic-specific Bmal1 deficiency increases atherosclerosis in *Apoe*$^{-/-}$ mice (45). Here, we used multiple *Apoe*$^{-/-}$ mouse models to address the role of Mφ-specific Bmal1 in atherosclerosis. First, we transplanted bone marrow cells from *Bmal1*$^{-/-}$ *Apoe*$^{-/-}$ or *Bmal1*$^{+/+}$ *Apoe*$^{-/-}$ mice into lethally irradiated *Apoe*$^{-/-}$ mice. After 4 weeks of Western diet (WD) feeding, greater atherosclerotic lesion areas, en face Oil Red O staining in the aortas, and collagen and Mφ content were observed in the aortic roots of *Apoe*$^{-/-}$ mice transplanted with bone marrow cells from *Bmal1*$^{-/-}$ *Apoe*$^{-/-}$ mice rather than *Bmal1*$^{+/+}$ *Apoe*$^{-/-}$ mice (Figure 1A). No significant differences were observed in body weight, plasma cholesterol, or triglycerides in *Apoe*$^{-/-}$ mice

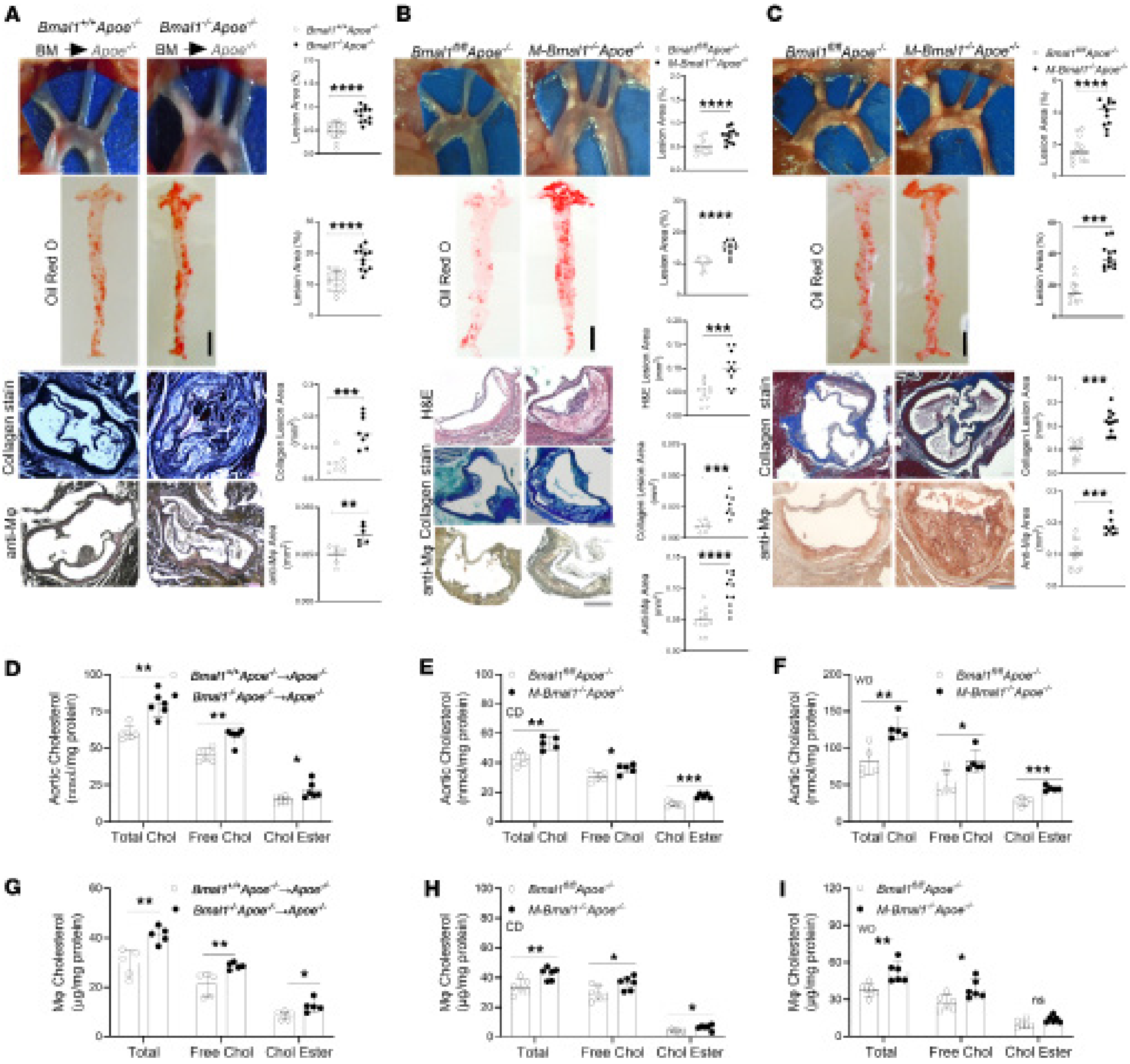

Figure 1. Mφ-specific Bmal1 deficiency increases atherosclerosis in various mouse models. (**A**) Lethally irradiated *Apoe*$^{-/-}$ mice (female, 8- weeks old, *n* = 12–15) were transplanted with bone marrow cells from *Bmal1*$^{-/-}$ *Apoe*$^{-/-}$ or *Bmal1*$^{+/+}$ *Apoe*$^{-/-}$ mice. Three months after transplantation, the animals were fed WD for 1 month. (**B**) *Bmal1*$^{fl/fl}$ *Apoe*$^{-/-}$ and *M-Bmal1*$^{-/-}$ *Apoe*$^{-/-}$ mice (male, *n* = 12) were fed a CD for 14 months. (**C**) *Bmal1*$^{fl/fl}$ *Apoe*$^{-/-}$ and *M-Bmal1*$^{-/-}$ *Apoe*$^{-/-}$ mice (male, 3–4 months old, *n* = 10–15) were fed a WD for 2 months. Aortas were collected at ZT 5. Aortic arches were dissected and photographed. Whole aortas were stained with Oil Red O, and images were quantified in ImageJ. Scale bar: 5 mm. Sections (10 μm) from cardiac/aortic junctions were stained with H&E to measure necrotic areas. Scale bar: 100 μm. Masson's trichrome and anti-Mφ antibodies to measure collagen and Mφ content. Scale bar: 100 μm. (**D**–**F**) Aortas were used to measure total, free, and esterified cholesterol. (**G**–**I**) BMDMs from multiple mouse models were cultured for 7 days and used to measure total, free, and esterified cholesterol (*n* = 5–6). Data are presented as mean ± SD, **P* < 0.05, ***P* < 0.01, ****P* < 0.001, and *****P* < 0.0001 versus control, 2-tailed, unpaired *t* test (**A**–**C**), or multiple unpaired *t* tests followed by Holm-Šídák method (**D**–**I**).

receiving cells from *Bmal1*$^{-/-}$ *Apoe*$^{-/-}$ or *Bmal1*$^{+/+}$ *Apoe*$^{-/-}$ control mice (Supplemental Figure 1, A and B; supplemental material available online with this article; https://doi.org/10.1172/jci.insight.194304DS1). Second, we fed myeloid-specific Bmal1-deficient *M-Bmal1*$^{-/-}$ *Apoe*$^{-/-}$ (*LysM*^{Cre}*-Bmal1*$^{fl/fl}$ *Apoe*$^{-/-}$) mice a chow diet (Figure 1B). Visualization of aortic arches and aortic Oil Red O staining revealed age-dependent increases in atherosclerosis in *M-Bmal1*$^{-/-}$ *Apoe*$^{-/-}$ mice, in contrast to *Bmal1*$^{fl/fl}$ *Apoe*$^{-/-}$ mice (Supplemental Figure 2, A and B). En face analysis of aortas at 14 months revealed elevated plaque formation in the aortic root and abdominal aorta in *M-Bmal1*$^{-/-}$ *Apoe*$^{-/-}$ mice (Figure 1B). Furthermore, the lesions

at the cardiac/aortic junction contained higher necrotic core, collagen, and Mφ content in *M-Bmal1*$^{-/-}$ *Apoe*$^{-/-}$ mice than *Bmal1*$^{fl/fl}$ *Apoe*$^{-/-}$ mice. Higher collagen content, perhaps, indicates the presence of stable plaques. Total plasma, HDL and non-HDL triglyceride, and cholesterol levels did not differ between *M-Bmal1*$^{-/-}$ *Apoe*$^{-/-}$ and *Bmal1*$^{fl/fl}$ *Apoe*$^{-/-}$ mice, as determined after separation by precipitation (Supplemental Figure 2, C and D) and by fast protein liquid chromatography (FPLC) (Supplemental Figure 2, E and F). Third, we studied the effect of WD feeding on atherosclerosis in *M-Bmal1*$^{-/-}$ *Apoe*$^{-/-}$ mice (Figure 1C). *Bmal1*$^{-/-}$ *Apoe*$^{-/-}$ mice showed greater atherosclerosis, lipid accumulation, collagen content, and Mφ infiltration than *Bmal1*$^{fl/fl}$ *Apoe*$^{-/-}$ mice (Figure 1C). Again, no significant differences were observed in plasma lipids in these groups (Supplemental Figure 3, A and B). We considered whether LysM^{Cre} might have affected atherosclerosis. However, LysM^{Cre} had no effect on atherosclerosis in *Apoe*$^{-/-}$ mice (Supplemental Figure 3, C and D). These studies indicate that Mφ-specific Bmal1 deficiency made *Apoe*$^{-/-}$ mice susceptible to atherosclerosis without affecting plasma lipid levels.

Increased Mφ cholesterol content in Bmal1-deficient Mφs. We previously showed that global Bmal1 deficiency increases plasma lipids and lipoproteins and may contribute to enhanced atherosclerosis (45). Here, we observed that Mφ-specific Bmal1 deficiency had no effect on plasma lipids or lipoproteins, suggesting that Mφ-Bmal1 affects atherosclerosis by unknown mechanisms. To unravel how Mφ-Bmal1 may contribute to atherosclerosis, we measured cellular cholesterol levels in different mouse models (Figure 1, D–F). Bmal1-deficient Mφs from different mouse models had higher levels of total, free, and esterified cholesterol levels compared with their respective controls (Figure 1, G–I). These studies suggest deregulation of cholesterol homeostasis in Bmal1-deficient Mφs. Attempts were then made to understand molecular mechanisms for increased cellular cholesterol accretions in Bmal1-deficient Mφs.

Bmal1 deficiency increases oxLDL uptake in Mφs. To elucidate the mechanisms for the elevated Mφ cholesterol levels during Bmal1 deficiency, we examined 3 contributing factors: uptake of modified lipoproteins, cellular cholesterol efflux, and lysosomal cholesterol egress. First, we hypothesized that Bmal1-deficient aortas and Mφs would show enhanced oxLDL uptake, thereby contributing to atherosclerosis. Chow-fed *Bmal1*$^{+/+}$ *Apoe*$^{-/-}$ and *Bmal1*$^{-/-}$ *Apoe*$^{-/-}$ mice were injected with Dil-labeled oxLDL. The aortas harvested from *Bmal1*$^{-/-}$ *Apoe*$^{-/-}$ mice showed ~2.5-fold higher assimilation of Dil-labeled oxLDL than controls (Figure 2A). ~~Similarly, *M-Bmal1*$^{fl/fl}$ *Apoe*$^{-/-}$ aortas showed increased oxLDL uptake compared with control *M-Bmal1*$^{fl/fl}$ *Apoe*$^{-/-}$ mice (Supplemental Figure 4)~~. Greater uptake of Dil-oxLDL was also observed in the aortas of *Apoe*$^{-/-}$ mice transplanted with *Bmal1*$^{-/-}$ *Apoe*$^{-/-}$ bone marrow cells rather than *Bmal1*$^{+/+}$ *Apoe*$^{-/-}$ bone marrow cells (Figure 2B). In addition, the aortas harvested from *M-Bmal1*$^{-/-}$*Apoe*$^{-/-}$ mice showed approximately 3.3-fold higher assimilation of DiI-labeled oxLDL than *Bmal1*$^{fl/fl}$*Apoe*$^{-/-}$ controls (Supplemental Figure 4). Thus, Bmal1 deficiency increased aortic oxLDL uptake.

Next, we isolated bone marrow–derived Mφs (BMDMs) from these mice and studied the uptake of Dil-oxLDL and [^{3}H]-cholesterol–labeled acetylated LDL (acLDL). Bmal1-deficient BMDMs from all mouse models took up more Dil-oxLDL (Figure 2C and Supplemental Figure 5A) and [^{3}H]-cholesterol–acLDL (Figure 2D and Supplemental Figure 5B), and they contained higher amounts of lipid peroxides compared with their respective controls (Figure 2E and Supplemental Figure 5C). These studies indicate that Bmal1-deficient BMDMs take up more modified lipoproteins and contain higher amounts of oxidized lipids than control BMDMs.

Bmal1 deficiency increases Cd36 expression and uptake of modified lipoproteins by decreasing Rev-erbα expression. To identify receptors involved in the increased uptake of modified lipoproteins, we measured the mRNA and protein levels of several scavenger receptors (Figure 2, F and G, and Supplemental Figure 5, D–F). Bmal1-deficient Mφs had higher mRNA and protein levels of Cd36 and Lox-1 than their respective controls, whereas the Sra1 mRNA and protein levels did not differ. Thus, Bmal1 deficiency specifically increases Cd36 and Lox-1 expression.

Next, we assessed whether Bmal1 might regulate Cd36 and Lox-1 in Mφs. WT Mφs were treated with siControl or siBmal1. siBmal1 significantly reduced Bmal1 mRNA and protein levels and increased Cd36 mRNA levels, but it had no effect on Lox-1 mRNA levels (Figure 2H and Supplemental Figure 5G). Moreover, siBmal1 significantly increased oxLDL uptake, and this uptake was inhibited in siCd36-treated cells (Figure 2I). These findings suggest that Bmal1 might regulate Cd36, thereby controlling the uptake of modified lipoproteins.

Bmal1 is a transcriptional enhancer. Because knockdown (KD) of Bmal1 increased Cd36 expression, we hypothesized that Bmal1 might decrease the expression of a repressor of Cd36. Bmal1 is known to

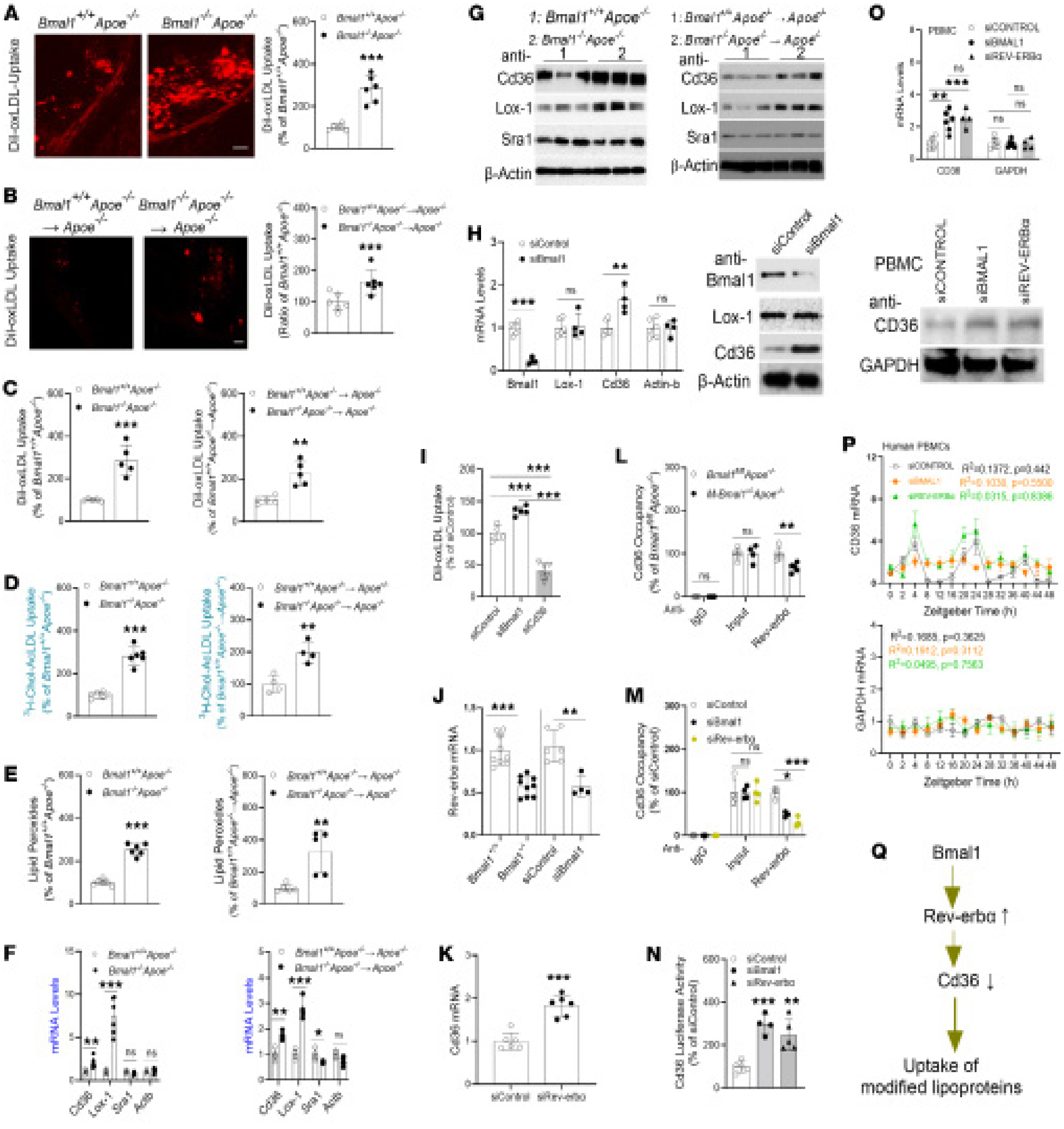

Figure 2. Mφ Bmal1 deficiency increases cellular cholesterol content, oxLDL uptake and Cd36 expression. (**A** and **B**) Mice were injected with Dil-ox-LDL (30 μg protein/50 μL) at ZT 12. After 18 hours, cardiac/aortic sections were photographed and quantified. Scale: 200 μm. (**C** and **D**) BMDMs were incubated with Dil-oxLDL for 4 hours (**C**), or with 5 μCi/mL [^{3}H]-cholesterol-acLDL (**D**) to measure uptake. (**E**) TBARS in BMDMs were measured. (**F** and **G**) BMDMs were used to measure mRNA and protein levels. (**H**) WT BMDMs were transfected with siControl or siBmal1. After 48 hours, RNA and protein levels were measured. (**I**) BMDMs were treated with different siRNA for 48 hours and incubated with Dil-oxLDL for 4 hours. (**J**) Rev-erbα mRNA levels were significantly lower in *Bmal1*$^{-/-}$ BMDMs than control mice (left, *n* = 9). KD of Bmal1 in BMDMs decreased Rev-erbα expression. (**K**) KD of Rev-erbα increased Cd36 expression. (**L**) Mφs were subjected to ChIP to determine the binding of Rev-erbα to the Cd36 promoter. (**M**) BMDMs were treated with different siRNA. After 48 hours, they were used to study the binding of Rev-erbα to the Cd36 promoter. (**N**) KD of clock genes increased Cd36 promoter activity. (**O**) Human differentiated PBMCs (2.0 × 10^6) were transfected with different siRNAs for 48 hours to measure mRNA (top) and protein (bot¬tom) levels. (**P**) BMAL1 or REV-ERBα KD abolished the cyclic expression of CD36 in Mφs, *n* = 3. (**Q**) Under normal conditions, Bmal1 increases expression of Rev-erbα, which acts as a repressor of CD36 and limits the uptake of modified lipoproteins. All values are mean ± SD, *n* = 4–6, **P* < 0.05, ***P* < 0.01 and ****P* < 0.001, 2-tailed, unpaired *t* test (**A**, **E**, and **K**), multiple *t* tests, followed by Holm-Šídák method correction (**F**, **H**, **L**, and **J**), or 1-way ANOVA with Tukey's test (**I**, **M**, **N**, and **Q**) or Cosinor (**P**).

increase the expression of Rev-erbα, a transcriptional repressor (24, 49–51). Indeed, we observed diminished Rev-erbα mRNA levels in *Bmal1*$^{-/-}$ Mφs and in siBmal1-treated WT Mφs (Figure 2J). Furthermore, siRev-erbα increased Cd36 expression (Figure 2K), thus indicating that Rev-erbα is a repressor of Cd36. ChIP analysis indicates that Rev-erbα interacted with the ROR element in the Cd36 promoter, and this binding was significantly diminished in Mφs obtained from *M-Bmal1*$^{-/-}$ *Apoe*$^{-/-}$ mice (Figure 2L~~ and Supplemental Figure 5L~~). In WT Mφs, Rev-erbα occupancy on the Cd36 promoter was significantly diminished in cells treated with siBmal1 but to a lesser extent than that observed in siRev-erbα–treated Mφs (Figure 2M). Furthermore, we obtained plasmids for expression of luciferase (pGL4.11-Cd36 promoter) under control of the Cd36 promoter (52). siBmal1 and siRev-erbα significantly increased luciferase activity (Figure 2N). These studies indicate that Rev-erbα reduced Cd36 expression.

Next, we extended these experiments to human monocyte-derived Mφs. Normal peripheral blood mononuclear cells (PBMCs) were differentiated into Mφs and used to study the effects of KD of *BMAL1* and *REV-ERBα* on *CD36* expression. KD of *BMAL1* and *REV-ERBα* significantly increased *CD36* mRNA and protein expression (Figure 2O and Supplemental Figure 5H), thus indicating that *BMAL1* and *REV-ERBα* regulate CD36 expression in human Mφs. Next, we studied the role of BMAL1 and REV-ERBα in cyclic expression of CD36. In siControl-treated Mφs, CD36 expression showed cyclic expression with peaks at 4 and 24 hours after serum synchronization (Figure 2P). KD of BMAL1 abolished these cyclic changes, whereas KD of REV-ERBα had no effect on the cyclic changes but increased the amounts of mRNA at the peak levels. These studies indicate that Bmal1 determines the cyclic expression of CD36, whereas REV-ERBα determines the extent of expression. Overall, these findings indicate that Bmal1 increased Rev-erbα expression, which in turn decreased CD36 expression and the uptake of modified lipoproteins (Figure 2Q).

Mφ Bmal1 deficiency decreases cholesterol efflux and RCT. The above studies show that Mφ specific Bmal1 deficiency increased uptake of modified lipoproteins, and Mφs assimilate more cholesterol and lipid peroxides, potentially by increasing Cd36 expression. Cells increase cholesterol efflux and RCT to decrease cellular free cholesterol content. Therefore, we determined whether Mφ-specific Bmal1 might play a role in regulating cholesterol efflux from Mφs and in vivo RCT in multiple mouse models. To study RCT, we injected ^{3}H-cholesterol–labeled Mφs from control and Bmal1-deficient mice into WT or *Apoe*$^{-/-}$ mice (Figure 3A). In all cases, Bmal1-deficient Mφs, compared with control Mφs, showed defects in RCT, as evidenced by diminished amounts of cholesterol in the plasma, liver, and feces (Figure 3, A and B). Furthermore, BMDMs from WD-fed Bmal1-deficient mouse models showed defects in cholesterol efflux to extracellular apoA1 and HDL (Figure 3C and Supplemental Figure 6A). These in vivo and cell culture studies indicate that Bmal1 deficiency decreased Mφ cholesterol efflux capacity.

Upregulated cellular Abca1/Abcg1 expression augments cholesterol efflux and RCT, thereby decreasing cellular free cholesterol content. Acat1 increases conversion of free cholesterol to esterified cholesterol for storage in cytosolic lipid droplets (16). Therefore, we measured changes in Abca1, Abcg1, and Acat1 mRNA and protein levels, and we determined the roles of Mφ-specific Bmal1 in their regulation. Protein and mRNA levels of Abca1 and Abcg1 were significantly reduced (Figure 3, D and E, and Supplemental Figure 6, B–D) in all mouse models. Srb1 levels were diminished in global Bmal1-KO mice but remained unchanged in Mφ-specific Bmal1-deficient Mφs (Figure 3, D and E, and Supplemental Figure 6E). The expression of Acat1, an enzyme involved in cholesterol esterification, did not change (Figure 3, D and E, and Supplemental Figure 6E). Therefore, defects in cholesterol efflux in Bmal1-deficient Mφs might be secondary to lower expression of Abca1 and Abcg1 transporters.

Next, we used KD approaches to address the role of Bmal1 in the regulation of Abca1 and Abcg1. siBmal1 significantly decreased Abca1 and Abcg1 expression, without affecting Srb1 expression, and additionally decreased cholesterol efflux (Figure 3, F and G). To determine whether overexpression of Bmal1 might also affect Abca1/Abcg1 expression and cholesterol efflux, we transduced the J774A.1 mouse Mφ cell line with adenoviruses for expression of Bmal1. Cells transduced with Adv-Bmal1 showed elevated expression of Abca1 and Abcg1 as well as greater cholesterol efflux to apoA1 and HDL than observed in cells transduced with Adv control (Figure 3, H and I). These studies indicate that Bmal1 KD decreases, whereas Bmal1 overexpression increases, Abca1/Abcg1 expression and cholesterol efflux.

We subsequently studied cyclic expression of Abca1 and Abcg1 in Mφs isolated from control and Bmal1-deficient mice. Serum shock studies indicate robust temporal changes in the expression of Abca1 and Abcg1, with major peaks at 20 and 40 hours in WT Mφs. However, these peaks were absent in Bmal1-deficient Mφs (Figure 3J). Furthermore, similar reductions in Abca1 and Abcg1 expression were

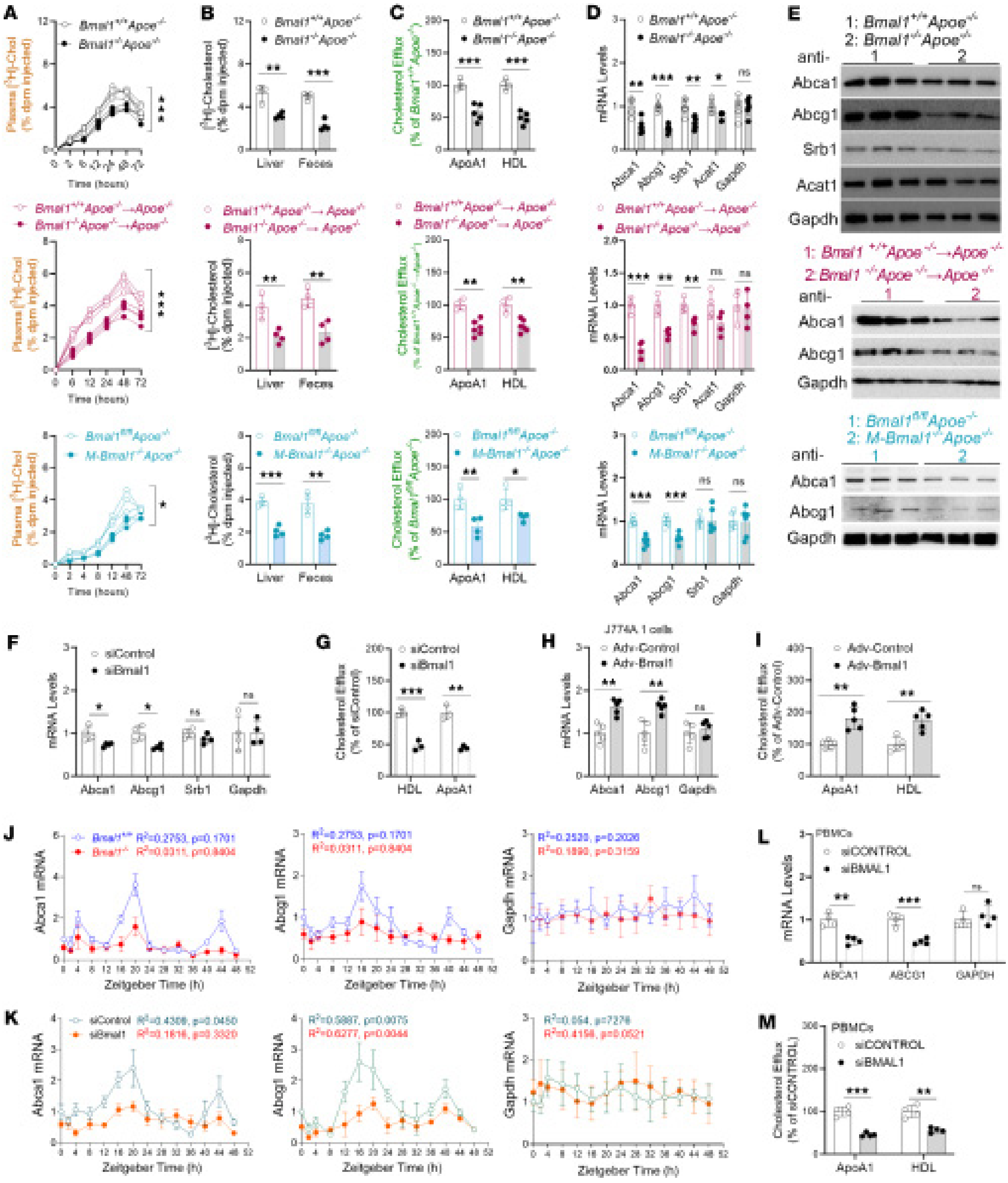

Figure 3. Mφ Bmal1 deficiency decreases cholesterol efflux and reverse cholesterol transport. (**A** and **B**) Mφs were incubated with $[^3H]$-cholesterol-acLDL for 24 hours and injected intraperitoneally at ZT 5 into WT mice, plasma (**A**), feces and liver (**B**) cholesterol were counted. (**C**) For efflux, Mφs were incubated with $[^3H]$-cholesterol-acLDL for 24 hours, washed, and incubated with purified ApoAI or HDL for 8 hours. (**D** and **E**) Mφs were used to measure mRNA (**D**) and protein (**E**) levels of cholesterol efflux-associated genes. (**F**) WT Mφs were treated with different siRNA. After 48 hours, mRNA levels of cholesterol efflux transporters were quantified. (**G**) SiControl or siBmal1-treated WT Mφs were incubated with $[^3H]$-cholesterol and acLDL for 18 hours, washed, and incubated with purified ApoAI or HDL for 8 hours to measure cholesterol efflux (n = 3). (**H** and **I**) *M-Bmal1*$^{-/-}$ Mφs were transduced with Adv-Control or Adv-Bmal1. After 72 hours, mRNA levels (**H**) and cholesterol efflux (**I**) were quantified. (**J**) BMDMs were collected at ZT5 (12:00) and

cultured for 7 days. Differentiated Mφs were subjected to 2 hours serum shock (9:30–11:30 AM) and collected at different times to measure mRNA levels. (**K**) BMDMs from *Bmal1*$^{fl/fl}$ mice were transfected with siBmal1 or siControl for 48 hours and subjected to 2 hours serum shock (9:30–11:30 AM). Mφs collected at different times were used to measure mRNA levels. (**L** and **M**) Human PBMCs (2.0 × 10^6) were differentiated into Mφs, transfected with the indicated siRNAs for 48 hours and subjected to gene expression (**L**) and cholesterol efflux (**M**) studies. All values are presented as mean ± SD, n = 4-6, $^*P < 0.05$, $^{**}P < 0.01$; $^{***}P < 0.001$, compared with control, multiple t tests followed by Holm-Šídák method (**B**–**D**, **F**–**I**, **L**, and **M**), 2-way ANOVA multiple comparisons (**A**) or Cosinor (**J** and **K**).

observed in WT Mφs treated with siBmal1 (Figure 3K). These studies indicate Bmal1's involvement in the temporal regulation of Abca1/Abcg1.

Next, we extended these experiments to human PBMCs to study the effects of KD of *BMAL1* on *ABCA1* and *ABCG1* gene expression and cholesterol efflux. KD of *BMAL1* significantly decreased *ABCA1* and *ABCG1* expression, as well as cholesterol efflux (Figure 3, L and M), thus indicating that Bmal1 also regulates cholesterol efflux in human Mφs.

Regulation of mouse Abca1 and Abcg1 by Bmal1. We previously demonstrated that Clock modulates Abca1 expression by regulating the Usf2 repressor (44). Here, we determined how Bmal1 regulates Abca1 and Abcg1, by examining expression changes in several repressors known to regulate Abca1 and Abcg1. Quantification of various mRNA and proteins in *Bmal1*$^{-/-}$ Mφs indicated significantly elevated (2- to 4-fold) Znf202 levels (Figure 4, A and B, and Supplemental Figure 7). Furthermore, siBmal1 increased Znf202 expression (Figure 4C). Thus, Znf202 might be regulated by Bmal1.

ZNF202 is a repressor (50) that controls the tissue-specific expression of *ABCA1* and *ABCG1* (54). Both human and mouse *ABCA1* and *ABCG1* promoters contain GnT motifs recognized by ZNF202 (53, 55, 56). Znf202 expression is downregulated during monocyte differentiation and foam cell formation (53, 55, 56). However, whether Znf202 shows diurnal rhythms, and might be involved in the diurnal regulation of Abca1 and Abcg1 expression, is unknown. Therefore, we assessed Znf202's potential involvement in regulating Abca1/Abcg1 and cholesterol efflux. siZnf202 increased Abca1/Abcg1 expression and cholesterol efflux, thus indicating that Znf202 is a repressor (Figure 4, D and E). To further determine whether Znf202 might act at the promoter level, we transfected WT BMDMs with a reporter construct for expression of luciferase under control of the Abca1 promoter, along with various siRNAs (Figure 4F). siBmal1 decreased Abca1 promoter luciferase activity, whereas siZnf202 increased promoter activity by 2.4-fold more than in the siControl group (Figure 4F). The role of Znf202 in regulating Abca1 and Abgc1 was further studied with ChIP (Figure 4G). Znf202 binding on the Abca1/Abcg1 promoters was significantly enhanced after KD of Bmal1. Therefore, Znf202 represses Abca1 expression at the transcriptional level, and Bmal1 regulates Abca1/Abcg1 by regulating Znf202 (Figure 4H).

Next, we extended these studies to human monocyte-derived Mφs. KD of BMAL1 in PBMCs decreased cholesterol efflux to apoA1 and HDL (Figure 5A), decreased the expression of ABCA1 and ABCG1, and increased the expression of ZNF202 (Figure 5, B and C, and Supplemental Figure 8). We then studied the binding of ZNF202 to the ABCA1 and ABCG1 promoters in CONTROL and siBMAL1-treated Mφs. siBMAL1 increased ZNF202 binding to these promoters (Figure 5D). Our findings indicate that ZNF202 binding to the ABCA1/G1 promoters was increased in Bmal1 deficiency, thus decreasing the expression of these transporters in human Mφs.

Lysosomes in Bmal1-deficient Mφs show enhanced cholesterol accumulation due to decreased Npc1 and Npc2 expression. After having demonstrated that elevated uptake of modified lipoproteins and decreased cholesterol efflux in Bmal1-deficient Mφs contributes to increasing cholesterol assimilation, we next sought to identify the subcellular organelles assimilating cholesterol. We subjected Mφs to differential ultracentrifugation (57, 58) and determined their purity by detecting specific markers (Supplemental Figure 9). Total, free, and esterified cholesterol levels were significantly higher in all subcellular organelles in *Bmal1*$^{-/-}$ *Apoe*$^{-/-}$ Mφs than *Bmal1*$^{+/+}$ *Apoe*$^{-/-}$ Mφs (Figure 6A); however, the highest accretions were in lysosomes and endosomes. Therefore, we sought to understand the mechanisms underlying cholesterol accumulation in lysosomes. Mφs were incubated with [^{3}H]-cholesterol–labeled acLDL, and lysosomal cholesterol levels were quantified at various times. Lysosomes of *Bmal1*$^{-/-}$ *Apoe*$^{-/-}$ Mφs showed greater cholesterol accumulation over time (Figure 6B). We hypothesized that this accumulation might have been due to defects in cholesterol egress from lysosomes. To examine this possibility, we pulse-labeled Mφs with [^{3}H]-cholesterol–acLDL for 4 hours, washed them, and incubated them in serum-free medium for chase experiments. At various times, lysosomes were isolated, and cholesterol egress from lysosomes was quantified (Figure 6C). Lysosomes from *Bmal1*$^{-/-}$ *Apoe*$^{-/-}$ Mφs showed less cholesterol egress than controls (Figure 6C). We

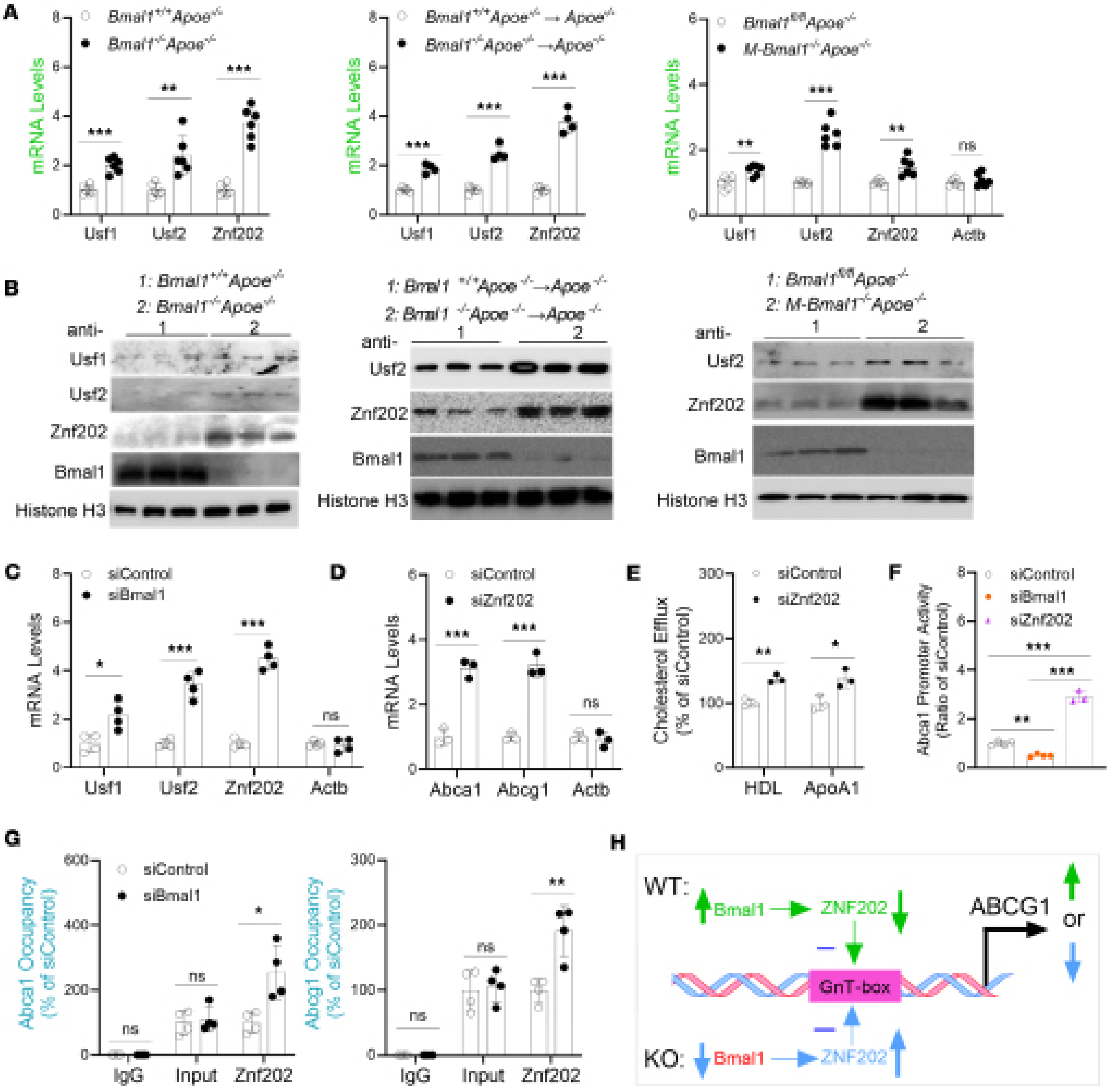

Figure 4. Identification of transcription factors regulating Abca1/Abcg1 in Bmal1-deficient Mφs. (**A** and **B**) Mφs from various mouse models were used to measure mRNA (**A**, *n* = 6) and protein (**B**) levels of cholesterol efflux transport-associated TFs. (**C**) WT Mφs were treated with siControl or siBmal1 for 48 hours and used to quantify mRNA levels (*n* = 4). (**D**) Mφs were transfected with siControl or siZnf202. After 48 hours, mRNA levels of Abca1 and Abcg1 were quantified. siZnf202 increased the expression of Abca1/Abcg1 (*n* = 3). (**E**) WT BMDMs were treated with siControl or siZnf202. After 48 hours, cholesterol efflux was quantified. siZnf202 increased cholesterol efflux in Mφs (*n* = 3). (**F**) WT BMDMs were transfected with a plasmid for expression of luciferase under the control of the Abca1 promoter. After 24 hours, cells were treated with siControl, siBmal1, or siZnf202, and luciferase activity was quantified after 48 hours (*n* = 3–4). (**G**) siControl- and siBmal1-treated Mφs were used to study binding of Znf202 to the Abca1 and Abcg1 promoters (*n* = 4). (**H**) In WT Mφs, Bmal1 represses the expression of Znf202 (green). Low levels of Znf202 increase Abcg1 expression and cholesterol efflux. Bmal1 deficiency increases Znf202, which in turn interacts with the GnT-box of Abcg1, thereby repressing Abcg1 transcription and decreasing cholesterol efflux (blue). All values are presented as mean ± SD, *n* = 3–4, **P* < 0.05, ***P* < 0.01, ****P* < 0.001, compared with siControl, multiple *t* tests followed by Holm-Šídák method (**A**, **C**–**E**, and **G**) or 1-way ANOVA followed by Tukey's test (**F**).

extended these studies to Mφs isolated from other mouse models (Figure 6, D and E). In all Bmal1-deficient Mφs, lysosomal [^{3}H]-cholesterol accretion was elevated (Figure 6D), and egress was diminished (Figure 6E). Because cholesterol egress from lysosomes in Mφs depends on Npc1 and Npc2 (10, 59, 60), we

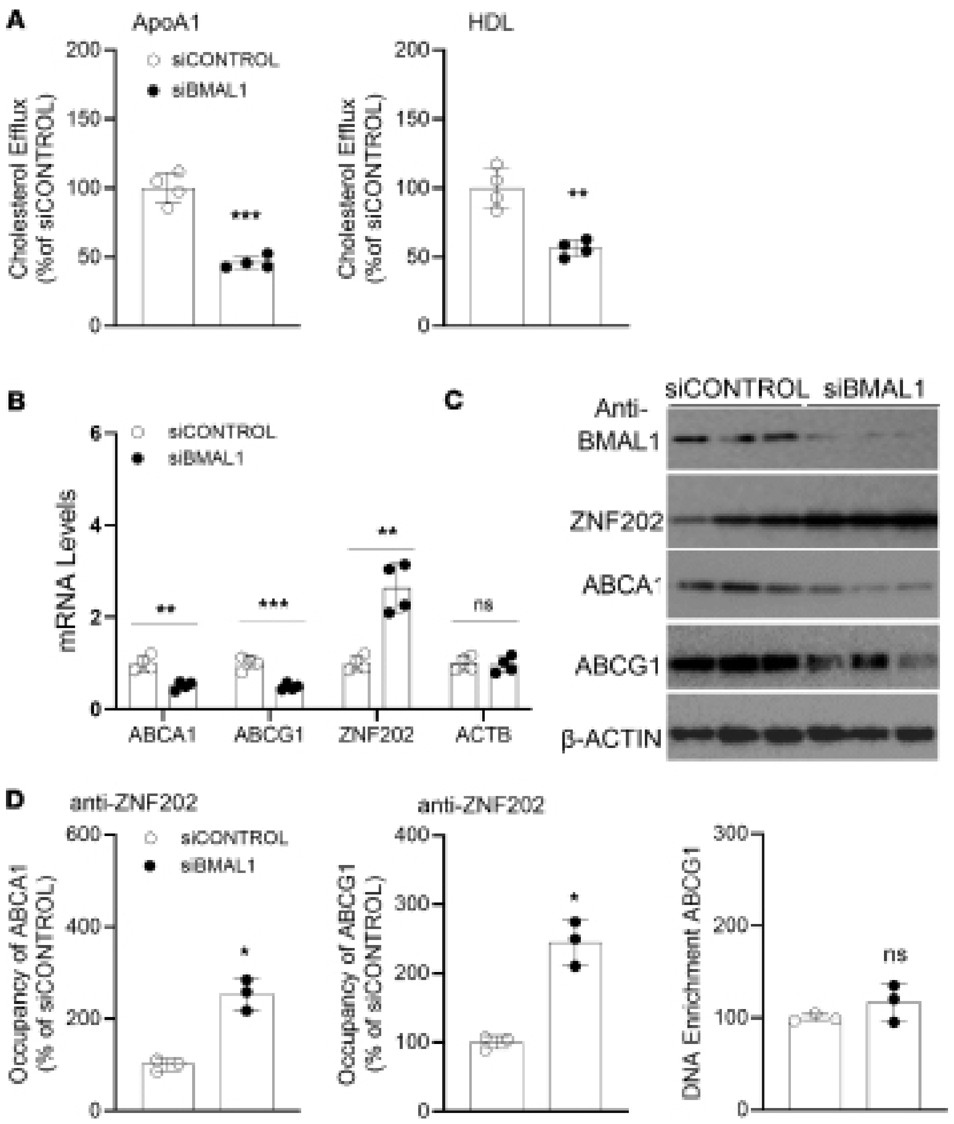

Figure 5. Bmal1 regulates ABCA1 expression by modulating Znf202 expression in human monocyte-derived macrophages. (**A**) Human PBMCs were treated with siCONTROL or siBMAL1. After 48 hours, cholesterol efflux to apoA1 and HDL were measured. siBmal1 decreased cholesterol efflux (n = 4). (**B** and **C**) Human PBMCs were treated with siCONTROL or siBMAL1. After 48 hours, mRNA (**B**, n = 4) and protein (**C**) levels of ABCA1, ABCG1, and ZNF202 were quantified. siBMALl1 increases ZNF202 expression. (**D**) Human PBMCs were treated with siCONTROL, siBMAL1, or ZNF202. After 48 hours, binding of ZNF202 to the ABCA1 or ABCG1 promoter was determined with ChIP. Sequences specific for the ABCA1 or ABCG1 promoter were amplified with qPCR (n = 3). All values are represented as mean ± SD. n = 3–4, *P < 0.05, **P < 0.01, ***P < 0.001 compared with control group, 2-tailed, unpaired t tests (**A** and **D**) or multiple t tests followed by Holm-Šídák method (**B**).

measured Npc1 and Npc2 mRNA and protein levels in Mφs isolated from various mouse models (Figure 6, F and G, and Supplemental Figure 10A). All Bmal1-deficient Mφs had diminished Npc1/Npc2 mRNA and protein levels. These results indicate that Bmal1 deficiency decreases the expression of Npc1 and Npc2 in Mφs. Therefore, this decreased expression might contribute to increased cholesterol accumulation in lysosomes in Bmal1-deficient Mφs.

Next, we extended these studies to human monocyte-derived Mφs. PBMCs were treated with siBMAL1, siNPC1, or siNPC2 and incubated with [^{3}H]-cholesterol–acLDL for 4 hours; subsequently, the radioactivity in lysosomes was quantified. The various siRNAs specifically decreased their targets' mRNA and protein levels (Figure 6H and Supplemental Figure 10B). In addition, siBMAL1 decreased the expression of *NPC1* and *NPC2*. Individual KD of BMAL1, NPC1, or NPC2 significantly decreased cholesterol egress (Figure 6I) and increased lysosomal cholesterol accretion (Figure 6J). We then asked whether *NPC1* and *NPC2* mRNA

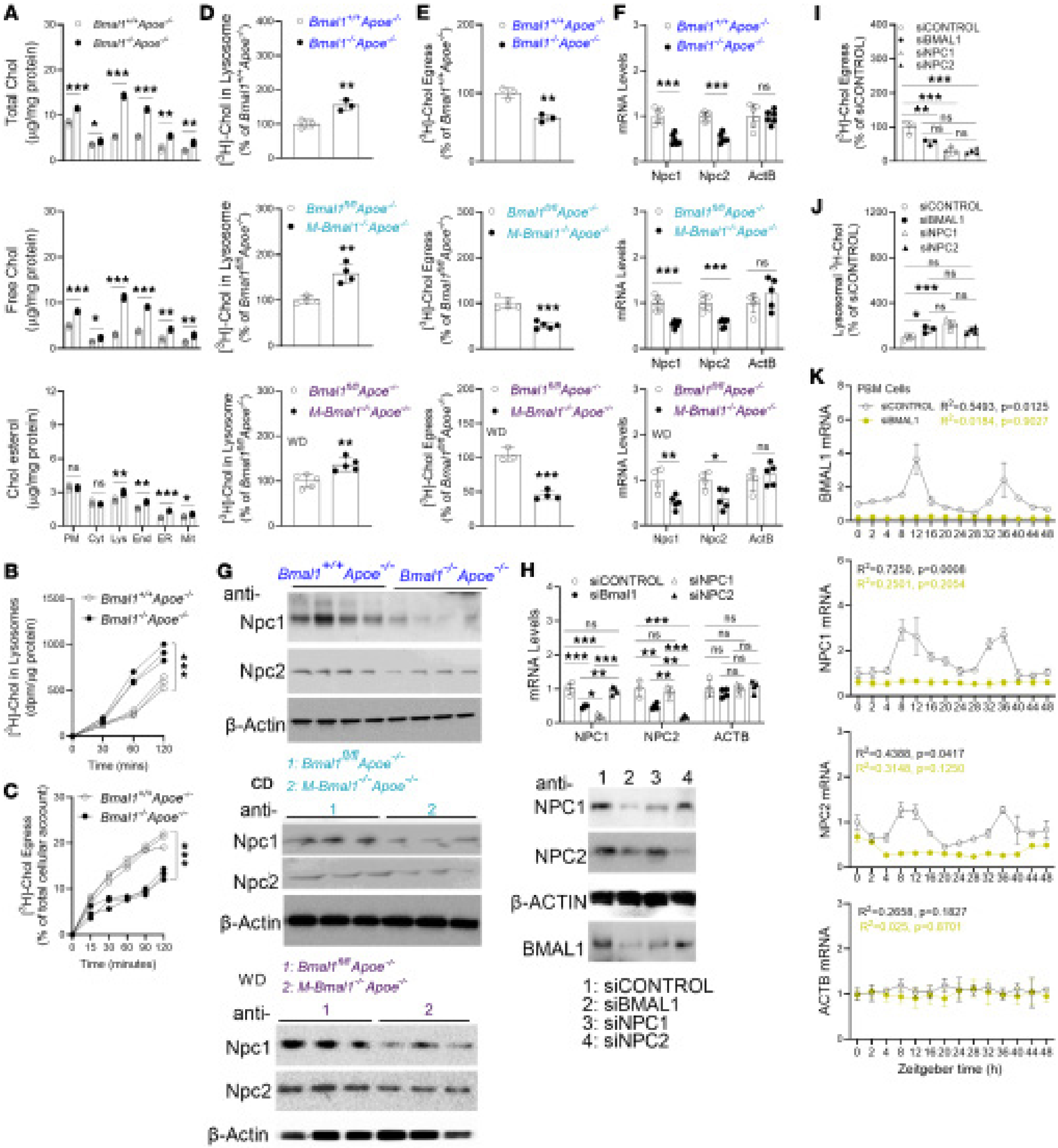

Figure 6. Bmal1 deficiency increases cholesterol assimilation and decreases cholesterol egress in lysosomes. (**A**) Various organelles were isolated from BMDMs, and lipids were quantified. (**B**) Mφs were incubated with 5 μCi/mL [^{3}H]-cholesterol-acLDL for various times. Counts in lysosomes were normalized to protein levels. (**C**) Mφs were pulse labeled in triplicates with 5 μCi/mL [^{3}H]-cholesterol-acLDL for 4 hours. The amounts of cholesterol at time 0 were set at 100%. Cholesterol remaining in lysosomes was used to calculate egress (n = 3). (**D** and **E**) Cultured BMDMs were pulse labeled in triplicate with 5 μCi/mL [^{3}H]-cholesterol-acLDL for 2 hours. Amounts in lysosomes were counted and normalized to protein (**D**), and lysosomal cholesterol egress were measured (**E**). (**F** and **G**) BMDMs were used to measure mRNA (**F**) and protein (**G**) levels. (**H**) Mφs from human PBMCs (2.0 × 10^6) were transfected with siRNAs for 48 hours and used to measure mRNA (top) and protein (bottom). (**I**) For lysosomal cholesterol egress measurements, Mφs were pulse labeled with 5 μCi/mL [^{3}H]-cholesterol for 4 hours, washed, and incubated in fresh medium for 2 hours. Lysosomes were then purified, and [^{3}H]-cholesterol counts were measured to calculate egress. (**J**) BMDMs were treated with various siRNAs for 48 hours and then supplemented with [^{3}H]-cholesterol for 4 hours. Lysosomal cholesterol levels were measured. (**K**) Human PBMC-derived Mφs were transfected with siRNAs. BMAL1 KD abolished the cyclic expression of NPC1 and NPC2 in Mφs. All values are represented as mean ± SD, n = 3–4, *P < 0.05, **P < 0.01, and ***P < 0.001 compared with control, 2-tailed, unpaired t tests (**D** and **E**) or multiple t tests followed by Holm-Šídák method (**A** and **F**) or 1-way ANOVA followed by Tukey's test (**H**–**J**), 2-way ANOVA (**B** and **C**), or Cosinor (**K**).

might show cyclic changes in response to serum synchronization and whether Bmal1 might play roles in their temporal expression. To examine this possibility, we subjected siCONTROL- and siBMAL1-treated PBMCs to serum synchronization. Bmal1 showed cyclic expression, with 2 peaks at 8–16 and 32–40 hours in siControl-treated cells (Figure 6K). Bmal1 expression was not observed in siBMAL1-treated human PBMCs. Both NPC1 and NPC2 showed similar peak expressions at 8–12 and 32–36 hours (Figure 6K). In siBMAL1-treated cells, the expression of NPC1 and NPC2 was significantly diminished and did not exhibit any significant temporal changes. Therefore, decreases in BMAL1 lowered NPC1/NPC2 temporal expression in PBMCs, most likely leading to defects in cholesterol egress from lysosomes.

We subsequently extended these studies to the mouse Mφ J774A.1 cell line. Npc1 and Npc2 showed cyclic expression, whereas these changes were absent in siBmal1-treated J774 cells (Figure 7A). KD of Bmal1 decreased the expression of Npc1/Npc2 (Figure 7B and Supplemental Figure 11A) and cholesterol egress (Figure 7C) as well as increased cholesterol levels in lysosomes (Figure 7C). These studies highlight the importance of Bmal1 in the control of basal and cyclic expression of Npc1 and Npc2 along with its role in lysosomal cholesterol trafficking. Subsequently, we increased the expression of Bmal1 by using Adv-Bmal1. Overexpression of Bmal1 increased Npc1/Npc2 expression (Figure 7D and Supplemental Figure 11B) and cholesterol egress and decreased lysosomal cholesterol (Figure 7E). These findings indicate that Bmal1 regulates Npc1/Npc2 and lysosomal cholesterol trafficking.

Because the Npc1 and Npc2 promoters contain E-boxes, we quantified E-box occupancy by Bmal1 and Clock in control and Bmal1-deficient Mφs (Figure 7, F and G). Bmal1 interacted with the E-boxes in WT Mφs, whereas this binding was not observed in Bmal1-deficient Mφs (Figure 7, F and G). In agreement with this finding, no E-box sequences were amplified after anti-Bmal1 ChIP. Use of anti-Clock IgGs during ChIP indicated that Clock also binds E-boxes, and this binding decreased in the absence of Bmal1 (Figure 7, F and G). These studies suggest that Bmal1 directly interacts with E-boxes in the promoters of Npc1/Npc2, thereby increasing their expression and cholesterol egress from lysosomes.

Discussion

Two prior studies have investigated the role of Mφ Bmal1 in atherosclerosis and reached opposite conclusions, suggesting that Mφ-specific Bmal1 deficiency either enhances (47) or decreases (48) atherosclerosis. In several various mouse models, we found that Bmal1 deficiency significantly increased atherosclerosis. First, we observed that transplantation of bone marrow cells from *Bmal1*$^{-/-}$ *Apoe*$^{-/-}$ mice into *Apoe*$^{-/-}$ mice significantly enhanced atherosclerosis (Figure 1A). Second, M-*Bmal1*$^{-/-}$ *Apoe*$^{-/-}$ mice developed significantly more atherosclerotic plaques than *Apoe*$^{-/-}$ mice fed either chow (Figure 1B) or a WD (Figure 1C). Thus, in various mouse models, Mφ Bmal1 deficiency enhanced atherosclerosis. We consequently suggest that Bmal1 protects against atherosclerosis by regulating Mφ function.

Hyperlipidemia and inflammation contribute to atherosclerosis. We observed that Mφ Bmal1 deficiency enhanced atherosclerosis without affecting plasma lipoproteins. Thus, Mφ Bmal1 regulated atherosclerosis by acting at the cellular level. Indeed, we found that Bmal1 regulated 3 different processes that control cellular cholesterol homeostasis. Our molecular studies show that Bmal1 directly enhanced transcription by binding E-boxes in the promoters of Npc1 and Npc2. Therefore, Npc1 and Npc2 mRNA and protein levels were increased by Bmal1 overexpression and decreased by Bmal1 KD. In contrast, Bmal1 indirectly regulated the expression of Cd36, Abca1, and Abcg1 by regulating intermediary clock-controlled TFs. We demonstrated that Bmal1 regulated Rev-erbα, which in turn repressed Cd36 expression by interacting with ROR elements in the Cd36 promoter. Similar attempts to identify clock-controlled repressors of Abca1/Abcg1 have identified Znf202 as an intermediary TF that regulated Abca1/Abcg1 expression. Thus, Bmal1 regulates cholesterol metabolism in Mφ by directly and indirectly modulating the expression of critical proteins in cholesterol homeostasis.

Mφ cholesterol metabolism is regulated by the liver X receptor (LXR) and sterol regulatory element binding protein 2 (SREBP2) (61, 62). The LXR modulates cholesterol efflux and RCT by regulating Abca1 and Abcg1 (62). Our findings indicate that Bmal1 was an additional regulator of Mφ cholesterol metabolism modulating at least 3 pathways in Mφ cholesterol metabolism. Unlike LXR and SREBP2, Bmal1 not only regulated basal expression but also the temporal expression of several proteins in Mφ cholesterol homeostasis. The advantages that might be provided by temporal regulation of Mφ cholesterol metabolism remain unclear. In hepatocytes, temporal changes may optimize cholesterol metabolism in sync with fasting and feeding. Mφ cholesterol metabolism under normal conditions might also be tuned to daily feeding and fasting rhythms, given that the aorta is

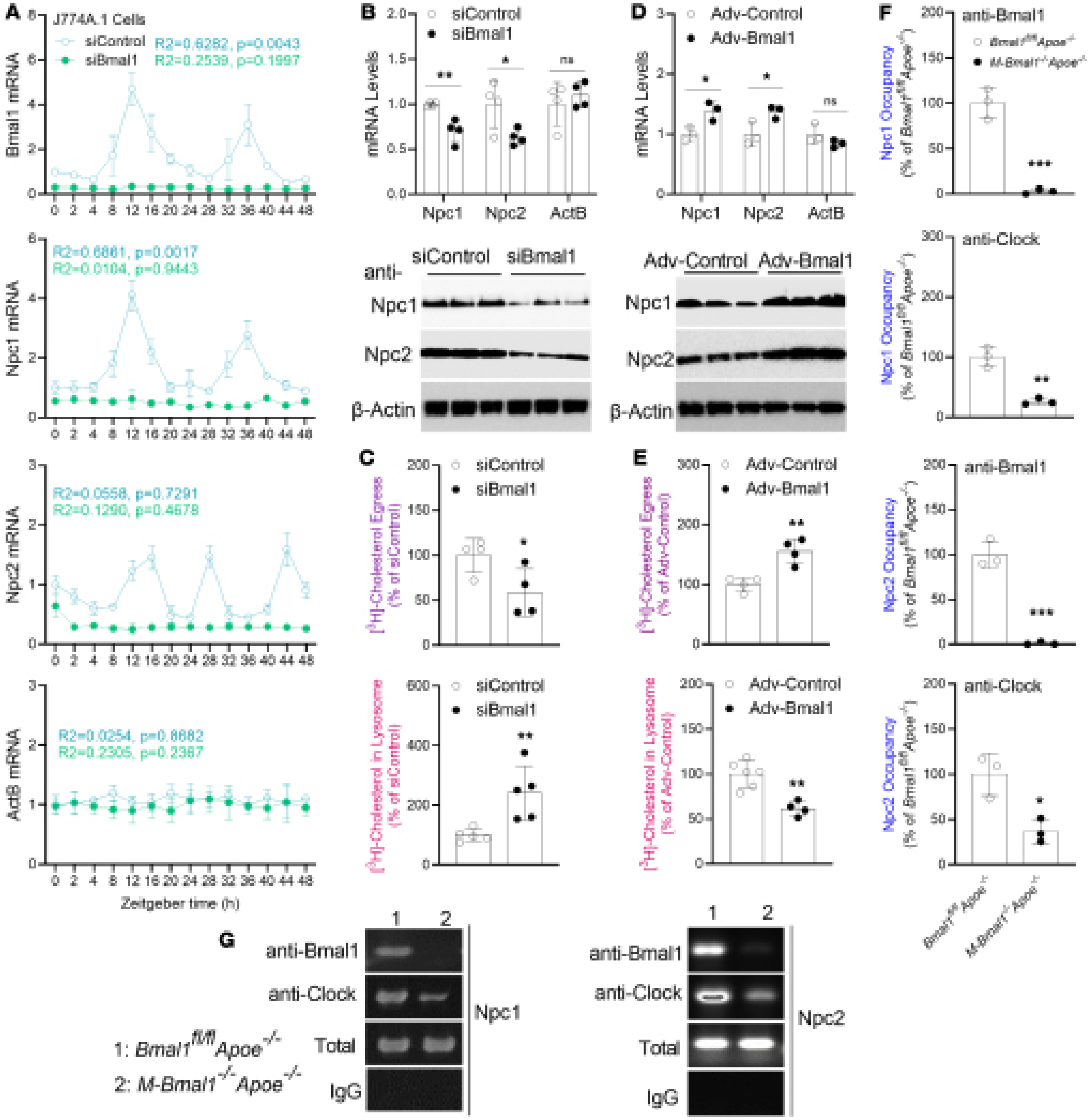

Figure 7. Bmal1 deficiency decreases Npc1 and Npc2 expression in J774A.1 Mφs. (**A**) Mouse J774A.1 Mφs were transfected with siBmal1 or siControl for 48 hours and subjected to 2 hours serum shock (9:30–11:30 am). Cells were collected at the indicated times to measure mRNA levels (*n* = 3). Values at time 0 (11:30 am) were normalized to 1. (**B**) J774A.1 Mφs were treated with various siRNAs, and mRNA and protein levels were measured after 48 hours (*n* = 4). (**C**) Cells were transfected with siControl or siBmal1. After 48 hours, they were labeled for 4 hours, and cholesterol accretion in lysosomes was quantified. For egress studies, cells were labeled with [^{3}H]-cholesterol for 4 hours. A zero-time point was collected. Other cells were then incubated in normal medium for 2 hours. Cholesterol counts in lysosomes at 0 hours and 2 hours were used to calculate egress (*n* = 4). (**D** and **E**) J774A.1 Mφs were transduced with Adv-Control or Adv-Bmal1. After 48 hours, Mφs were used to measure mRNA (*n* = 3) and protein levels (**D**, *n* = 3), as well as lysosomal cholesterol accretion and egress (**E**, *n* = 4–6). (**F** and **G**) BMDMs from 7-month-old male *M-Bmal1*$^{-/-}$ *Apoe*$^{-/-}$ and *Bmal1*$^{fl/fl}$ *Apoe*$^{-/-}$ mice were cultured for 7 days and used to study the binding of various circadian transcription factors to the Npc1 and Npc2 promoters with ChIP (*n* = 3). All values are mean ± SD, **P* < 0.05, ***P* < 0.01, and ****P* < 0.001 compared with Control group, 2-tailed, unpaired *t* tests (**C**, **E**, and **F**) or multiple *t* tests followed by Holm-Šídák method (**B** and **D**) or Cosinor analysis (**A**).

exposed to different amounts of lipoproteins at different times. Alternatively, temporal changes in Mφ cholesterol metabolism genes are secondary to the central control of whole-body circadian rhythms by Bmal1.

Our studies demonstrate that Bmal1 regulated the uptake of modified lipoproteins by upregulating

Cd36 expression. In Bmal1 deficiency, Cd36 expression was increased by low production of the Rev-erbα repressor. After uptake, modified lipoproteins were degraded at lysosomes, and cholesterol egress occurred from lysosomes to other subcellular organelles. This process was dependent on the Npc1 and Npc2 proteins, which shepherd cholesterol out of lysosomes. We demonstrated that Bmal1 directly regulated Npc1 and Npc2 expression by binding their promoters. After egress from lysosomes, most cholesterol reached the plasma membrane, which subsequently released cholesterol to extracellular acceptors such as apoA1 and HDL, in a process facilitated by Abca1 and Abcg1. We demonstrated that Bmal1 also regulated Abca1/Abcg1 through regulating the Znf202 repressor. Thus, 3 mechanisms controlling cellular homeostasis of cholesterol in Mφ were regulated by Bmal1.

We previously demonstrated that global and hepatocyte-specific Bmal1 deficiency increases plasma lipids and atherosclerosis, thus suggesting that hyperlipidemia might be a causal factor in atherosclerosis (45). In this study, we demonstrated that myeloid-specific Bmal1 deficiency did not affect circulating lipids and lipoproteins but significantly enhanced atherosclerosis in various mouse models. Thus, Bmal1 plays cell-specific roles in controlling cellular cholesterol metabolism and atherosclerosis. Others have shown that Mφ Bmal1 deficiency enhances atherosclerosis by affecting resident inflammatory Mφs and infiltrating Ly6c^{hi} monocytes (47). We also found that an inflammatory marker TNF-α was increased in Bmal1-deficient Mφs (Supplemental Figure 1, C–E). Thus, Mφ Bmal1 most likely regulates several processes to guard against atherosclerosis in *Apoe*$^{-/-}$ mice.

Atherosclerosis studies involving bone marrow transplantation are confounded by the genotypes of donor and recipient mice as well as by the ability of recipient mice to develop atherosclerosis. WT mice do not develop atherosclerosis; therefore, we selected *Apoe*$^{-/-}$ mice as recipients because they are widely used and develop robust atherosclerosis. For donors, we used *Bmal1*$^{-/-}$*Apoe*$^{-/-}$ and *Bmal1*$^{+/+}$*Apoe*$^{-/-}$ mice so that bone marrow cells are only deficient in Bmal1. The use of *Bmal1*$^{-/-}$ donor mice is contraindicated, as bone marrow cells from these mice express ApoE and this may regress atherosclerosis in *Apoe*$^{-/-}$ mice and the contribution of the Bmal1 deficiency cannot be properly assessed.

In summary, Bmal1 controls the temporal expression of multiple proteins involved in Mφ cholesterol homeostasis. By coordinating several pathways in cholesterol metabolism, Bmal1 may act as a master regulator. A better molecular level understanding of the regulation of Mφ functions in the pathogenesis of atherosclerosis through circadian-clock genes might provide opportunities for better diagnosis, prognosis, and therapeutic interventions.

Methods

Sex as a biological variable. We have used both male and female mice in this project.

Materials. [^{3}H]-Cholesterol (1 Ci/mL, 9.25 MBq, NET139250UC) was purchased from NEN Life Science Products. Chemicals and solvents were from various vendors (Supplemental Table 1).

Animals and diet. All mice were on a C57Bl6J background. *Bmal1*$^{+/-}$*Apoe*$^{-/-}$ mice were bred to obtain *Bmal1*$^{-/-}$ *Apoe*$^{-/-}$ and *Bmal1*$^{+/+}$ *Apoe*$^{-/-}$ mice. Various Mφ-specific Bmal1-deficient mouse strains were generated by crossing Bmal1 floxed (*Bmal1*$^{fl/fl}$) mice with C57BL/6J or *Apoe*$^{-/-}$ mice expressing the Cre recombinase transgene under control of the lysozyme M promoter (B6.129-Lyz2$^{tml(cre)Ifo}$/J, Jackson Laboratory). *M-Bmal1*$^{-/-}$ *Apoe*$^{-/-}$ *and Bmal1*$^{fl/fl}$ *Apoe*$^{-/-}$ mice were fed a WD containing protein, carbohydrates, fat, and cholesterol at 17%, 48.5%, 21.2%, and 0.2% by weight, respectively (TD 88137, Harlan Teklad).

Cell culture. BMDMs were obtained through isolation of bone marrow cells from female and male mice and through incubation in RPMI 1640 with 10% FBS and 1% penicillin-streptomycin supplemented with 25% L-cell–conditioned medium in tissue culture plates. Fresh medium was added on days 3 and 5. After 7 days, Mφs were fully differentiated, and the culture medium was changed to 1× DMEM with 1% penicillin-streptomycin supplemented with 5% L-cell–conditioned medium. In some experiments, after 7 days, cells were subjected to serum shock, cholesterol efflux assays, or Dil-oxLDL uptake assays. Cells were transfected with siRNAs or transduced with adenoviruses for KD and overexpression as previously described (27, 44, 45, 63, 64).

Human PBMCs were differentiated into Mφs by culturing in RPMI-1640 supplemented with 10% human serum and 25 ng/mL recombinant human Mφ colony-stimulating factor (M-CSF, PeproTech) for 7 days. PBMCs (2.0 × 10^6) were plated in 6-well cell culture plates in RPMI-1640 medium with L-glutamine. After 4 hours, cells were treated with siRNAs for 48 hours, before being subjected to cholesterol efflux assays, isolation of total RNA, or detection of protein levels.

Subcellular fractionation of Mφs. Cells were lysed in NP-40 buffer (25 mM Tris, pH 7.5, 300 mM NaCl, 1

mM EDTA, and 2% NP-40) with protease inhibitor cocktail for 30 min on ice. The lysates were centrifuged at 1,000*g* for 5 minutes. The supernatant contained the cytosolic and membrane fractions. The pellets were suspended in NP-40 lysis buffer, passed 10 times through a 25 G needle and subjected to differential ultracentrifugation (44, 57) and used to quantify cholesterol levels. We verified the following subcellular markers in each fraction: calnexin (ER marker), ferritin (mitochondrial marker), ERK1/2 (endosome marker), LAMP1 (lysosome marker), NPC1 (lysosome marker), Na,K-ATPase α1 (plasma membrane), and Gapdh (cytosol). In most experiments, the lysosome fraction was purified with a lysosome isolation kit (Abcam, Ab234047).

Measurement of cholesterol accumulation and egress in lysosomes. To study cholesterol accretion, we incubated Mφs in triplicates with 10 μCi/mL [^{3}H]-cholesterol–acLDL (50 μg/mL). At different times, Mφs were centrifuged (21,500*g*, 20 min, 4°C) and washed, and homogenates were subjected to cell fractionation to isolate organelles and were counted (44, 57). For egress studies, cells were pulse labeled with 10 μCi/mL [^{3}H]-cholesteryl–acLDL (50 μg/mL) for 4 hours. The amounts of cholesterol after 4 hours were set at 100% to calculate time-dependent egress from lysosomes during subsequent chase times. The cells were then washed twice quickly with 5 mL buffer A (150 mM NaCl and 50 mM Tris-chloride, pH 7.4) containing 0.25% BSA, refed with 5 mL medium (DMEM 10% LDS, 1% penicillin-streptomycin, and 20 mM HEPES, pH 7.4), and placed in a CO_2 incubator for the indicated chase times. At each time point, cells were collected, and lysosome fractions were isolated using kits. Lipids were extracted and separated on TLC plates, and cholesterol bands were quantified. Amounts in lysosomes were used to calculate accretions and egress.

Ex vivo cholesterol efflux from BMDMs, J774A.1 Mφs, or PBMCs. For gene expression studies, cells were placed in 10% DMEM plus 25% L-cell–conditioned medium for 1 week. Total RNA was extracted and analyzed with quantitative PCR (qPCR) (44, 45). For cholesterol efflux assays, BMDMs were labeled with [^{3}H]-cholesterol (5.0 μCi/mL) with acLDL (50 μg/mL) for 24 hours, washed with PBS, and incubated in DMEM containing 0.2% BSA for 1 hour and subsequently in the same medium with or without apoAI (15 μg/mL) or HDL (50 μg/mL) for 8 hours. Radioactivity in the medium and total cell-associated radioactivity were determined by scintillation counting. The assays were performed in quadruplicate and are presented as percentage efflux, as previously described (44, 45).

Uptake of Dil-labeled oxLDL by Mφs. BMDMs from various mouse models, such as *Bmal1*$^{fl/fl}$ *Apoe*$^{-/-}$ mice and *M-Bmal1*$^{-/-}$ *Apoe*$^{-/-}$ mice, were cultured in 10% DMEM plus 25% L-cell–conditioned medium for 1 week. BMDMs or PBMCs were incubated for 4 hours at 37°C with 8 μg/mL Dil-labeled oxidized LDL (L34358, Thermo Fisher Scientific, Invitrogen) in α-MEM containing 2.5% lipoprotein-deficient serum (Sigma, S5394). Cells were washed with PBS, then homogenized in 50 mM Tris-HCl buffer, pH 7.4, and 1.15% KCl, and centrifuged (900*g*, 10 min, 4°C). The supernatants were used to measure fluorescence as previously described (44). For some experiments, differentiated Mφs from WT mice were transfected with siBmal1 or siControl for 48 hours, before being incubated with Dil-oxLDL (5 μg/mL) in serum-free DMEM at 37°C for 4 hours.

Serum synchronization studies. J774A.1 cells, BMDMs, or PBMCs were transfected with various siRNAs. After 48 hours, the cells were washed and starved in the same medium without FBS for 18 hours. Subsequently, cells were treated with medium containing 50% horse serum for 2 hours, and the medium was subsequently changed back to starvation medium (27, 44, 45, 64). Cells were harvested at 4-hour intervals for analysis.

Plasma and Mφ lipid analyses and lipoprotein profiling. Plasma and Mφ cholesterol, free cholesterol, and triglycerides were measured with commercial kits. Lipoprotein profiles were determined after FPLC, as described previously (44, 45). Pooled plasma samples from 6 mice per genotype were used for FPLC.

Measurement of lipid peroxides. Lipid peroxides were measured in isolated Mφs as TBARS. Mφs were homogenized in 1.15% KCl in 50 mM Tris-HCl buffer, pH 7.4, and then centrifuged (10,800*g*, 50 min, 4°C). The supernatants were used to measure lipid peroxides with a TBARS assay kit (10009055, Cayman Chemical Company) (44, 45, 63).

In vivo RCT. BMDMs from various mouse models were loaded with cholesterol by incubation with acLDL (50 μg protein/mL) and 5 μCi [^{3}H]-cholesterol for 24 hours. The labeled Mφs were injected i.p. into WT mice. Plasma was collected at 0, 6, 12, 24, and 48 hours, and feces were collected at 48 hours to measure tracer counts, as previously described (44, 45).

Quantification of atherosclerosis. The proximal aorta was collected after saline perfusion. The aortic root and ascending aorta were sectioned at a thickness of 10 μm. Alternate sections were used for Oil Red O, H&E, Masson's trichrome, and Mφ staining, as previously described (44, 45).

Bone marrow transplantation. *Apoe*$^{-/-}$ mice (age 8 weeks) were lethally irradiated and transplanted with bone marrow cells derived from *Bmal1*$^{-/-}$ *Apoe*$^{-/-}$ mice and *Bmal1*$^{+/+}$ *Apoe*$^{-/-}$ mice, as previously described (27, 44).

Western blotting analysis. Proteins from tissues or cells were separated under nonreducing conditions, transferred to nitrocellulose membranes, and blocked for 2 hours in TBS buffer containing 0.1% Tween 20 and 5% nonfat dry milk at room temperature. The blots were washed 3 times and incubated overnight at 4°C in the same buffer containing 0.5% dry milk and primary antibodies (1:100–1:1,000 dilution), washed, and incubated with mouse horseradish peroxidase–conjugated secondary antibodies (1:1,000–1:4,000) in 1.0% skim milk for 1 hour at room temperature. Immunoreactivity was detected by chemiluminescence, as previously described (44, 45, 63).

qPCR. Total RNA from Mφs, J774A.1, and human PBMCs were isolated with TRIzol. Subsequently, cDNA was synthesized with an OmniScript RT (Qiagen) kit. mRNA levels were measured with a SYBR Green kit for qPCR. 18S rRNA was used as a reference control. The data were analyzed as arbitrary units, as previously described (44, 45, 64).

ChIP. ChIP assays using polyclonal antibodies were performed to study the binding of different TFs to gene promoters. Proteins were cross-linked to DNA and sheared, and protein/DNA complexes were immunoprecipitated with specific antibodies to various TFs. DNA samples recovered after immunoprecipitation were subjected to PCR to detect coimmunoprecipitated DNA with specific primers (Supplemental Table 1).

Statistics. All metabolic and imaging experiments were repeated at least twice on different days and yielded similar results. Data are presented as mean ± SD for n = 6–15 animals per time point. Statistical testing was performed with 2-tailed unpaired t test, or multiple unpaired t tests, with followed by Holm-Šídák method. Temporal comparisons between 2 groups were performed with 2-way ANOVA using Tukey's multiple-comparison test, as indicated in the figure legends. Three or 4 (multiple) groups were performed using 1-way ANOVA followed by Tukey's test. Differences were considered statistically significant when $P < 0.05$. GraphPad Prism 10 was used for graphing and statistical evaluation. The circadian rhythm patterns were identified by fitting a cosine curve to the data. R^2 measures the goodness of fit.

Study approval. All animal experiments were approved by the IACUC of SUNY Downstate Medical Center or NYU Long Island School of Medicine.

Data availability. Values for all data points in graphs are reported in the Supporting Data Values file.

Author contributions

XP conceived the ideas, designed and performed experiments, analyzed and interpreted data, provided supervision, wrote the draft of the article, and extensively revised the manuscript. JO, CM, and SM performed experiments and analyzed data. NW performed bone marrow transplantation experiments. MMH provided the study concept and supervision, interpreted data, and performed critical and extensive manuscript revisions. All authors read the drafts and approved the manuscript.

Funding support

This work is the result of NIH funding, in part, and is subject to the NIH Public Access Policy. Through acceptance of this federal funding, the NIH has been given a right to make the work publicly available in PubMed Central.

- NIH grants R56 HL137912 and R01HL169313 to XP.
- American Heart Association Scientist Development grant 2300158 and Grant-in-Aid 16GRNT30960027 to XP.
- NIH grants R01DK121490, R01HL137202, P01HL166214, R01HL158054 and R01HL160470 to MMH.
- VA Merit Award BX004113 to MMH.

Acknowledgments

The content is solely the responsibility of the authors and does not necessarily represent the official views of the VA and NIH.

Address correspondence to: Xiaoyue Pan, Department of Foundations of Medicine, NYU Grossman Long Island School of Medicine, 101 Mineola Blvd., Suite 4-004, Mineola, New York, 11501, USA. Phone: 516.663.3446; Email: xiaoyue.pan@nyulangone.org. Or to: M. Mahmood Hussain, Department of Foundations of Medicine, NYU Grossman Long Island School of Medicine, 101 Mineola Blvd., Suite 4-003, Mineola,

New York, 11501, USA. Phone: 516.663.1670; Email: Mahmood.hussain@nyulangone.org.

1. Nedkoff L, et al. Global trends in atherosclerotic cardiovascular disease. *Clin Ther*. 2023;45(11):1087–1091.
2. Bjorkegren JLM, Lusis AJ. Atherosclerosis: recent developments. *Cell*. 2022;185(10):1630–1645.
3. Moore KJ, et al. Macrophages in atherosclerosis: a dynamic balance. *Nat Rev Immunol*. 2013;13(10):709–721.
4. Olefsky JM, Glass CK. Macrophages, inflammation, and insulin resistance. *Annu Rev Physiol*. 2010;72:219–246.
5. Tabas I, Bornfeldt KE. Macrophage phenotype and function in different stages of atherosclerosis. *Circ Res*. 2016;118(4):653–667.
6. Tabas I. Macrophage death and defective inflammation resolution in atherosclerosis. *Nat Rev Immunol*. 2010;10(1):36–46.
7. Vogel A, et al. Lipid scavenging macrophages and inflammation. *Biochim Biophys Acta Mol Cell Biol Lipids*. 2022;1867(1):159066.
8. Chang TY, et al. Niemann-Pick type C disease and intracellular cholesterol trafficking. *J Biol Chem*. 2005;280(22):20917–20920.
9. Storch J, Xu Z. Niemann-Pick C2 (NPC2) and intracellular cholesterol trafficking. *Biochim Biophys Acta*. 2009;1791(7):671–678.
10. Meng Y, et al. Cholesterol handling in lysosomes and beyond. *Trends Cell Biol*. 2020;30(6):452–466.
11. Favari E, et al. Cholesterol efflux and reverse cholesterol transport. *Handb Exp Pharmacol*. 2015;224:181–206.
12. Baldan A, et al. ATP-binding cassette transporter G1 and lipid homeostasis. *Curr Opin Lipidol*. 2006;17(3):227–232.
13. Jessup W, et al. Roles of ATP binding cassette transporters A1 and G1, scavenger receptor BI and membrane lipid domains in cholesterol export from macrophages. *Curr Opin Lipidol*. 2006;17(3):247–257.
14. Oram JF, Heinecke JW. ATP-binding cassette transporter A1: a cell cholesterol exporter that protects against cardiovascular disease. *Physiol Rev*. 2005;85(4):1343–1372.
15. Wang X, et al. Macrophage ABCA1 and ABCG1, but not SR-BI, promote macrophage reverse cholesterol transport in vivo. *J Clin Invest*. 2007;117(8):2216–2224.
16. Westerterp M, et al. ATP-binding cassette transporters, atherosclerosis, and inflammation. *Circ Res*. 2014;114(1):157–170.
17. Tall AR, et al. Regulation and mechanisms of macrophage cholesterol efflux. *J Clin Invest*. 2002;110(7):899–904.
18. Linsel-Nitschke P, Tall AR. HDL as a target in the treatment of atherosclerotic cardiovascular disease. *Nat Rev Drug Discov*. 2005;4(3):193–205.
19. Yvan-Charvet L, et al. Combined deficiency of ABCA1 and ABCG1 promotes foam cell accumulation and accelerates atherosclerosis in mice. *J Clin Invest*. 2007;117(12):3900–3908.
20. Reppert SM, Weaver DR. Molecular analysis of mammalian circadian rhythms. *Annu Rev Physiol*. 2001;63:647–676.
21. Lowrey PL, Takahashi JS. Mammalian circadian biology: elucidating genome-wide levels of temporal organization. *Annu Rev Genomics Hum Genet*. 2004;5:407–441.
22. Gekakis N, et al. Role of the CLOCK protein in the mammalian circadian mechanism. *Science*. 1998;280(5369):1564–1569.
23. Darlington TK, et al. Closing the circadian loop: CLOCK-induced transcription of its own inhibitors per and tim. *Science*. 1998;280(5369):1599–1603.
24. Green CB, et al. The meter of metabolism. *Cell*. 2008;134(5):728–742.
25. Levi F, Schibler U. Circadian rhythms: mechanisms and therapeutic implications. *Annu Rev Pharmacol Toxicol*. 2007;47:593–628.
26. Bass J, Lazar MA. Circadian time signatures of fitness and disease. *Science*. 2016;354(6315):994–999.
27. Pan XY, et al. Diurnal regulation of MTP and plasma triglyceride by CLOCK is mediated by SHP. *Cell Metab*. 2010;12(2):174–186.
28. Balsalobre A, et al. A serum shock induces circadian gene expression in mammalian tissue culture cells. *Cell*. 1998;93(6):929–937.
29. Thosar SS, et al. Role of the circadian system in cardiovascular disease. *J Clin Invest*. 2018;128(6):2157–2167.
30. Quyyumi AA. Circadian rhythms in cardiovascular disease. *Am Heart J*. 1990;120(3):726–733.
31. Garaulet M, Madrid JA. Chronobiology, genetics and metabolic syndrome. *Curr Opin Lipidol*. 2009;20(2):127–134.
32. Garaulet M, et al. CLOCK gene is implicated in weight reduction in obese patients participating in a dietary programme based on the Mediterranean diet. *Int J Obes (Lond)*. 2010;34(3):516–523.
33. Scott EM, et al. Association between polymorphisms in the Clock gene, obesity and the metabolic syndrome in man. *Int J Obes (Lond)*. 2008;32(4):658–662.
34. Sookoian S, et al. Genetic variants of Clock transcription factor are associated with individual susceptibility to obesity. *Am J Clin Nutr*. 2008;87(6):1606–1615.
35. Woon PY, et al. Aryl hydrocarbon receptor nuclear translocator-like (BMAL1) is associated with susceptibility to hypertension and type 2 diabetes. *Proc Natl Acad Sci U S A*. 2007;104(36):14412–14417.
36. Brum MC, et al. Shift work and its association with metabolic disorders. *Diabetol Metab Syndr*. 2015;7:45.
37. Cheng WJ, et al. Night shift work and the risk of metabolic syndrome: Findings from an 8-year hospital cohort. *PLoS One*. 2021;16(12):e0261349.
38. Ha M, Park J. Shiftwork and metabolic risk factors of cardiovascular disease. *J Occup Health*. 2005;47(2):89–95.
39. Kawachi I, et al. Prospective study of shift work and risk of coronary heart disease in women. *Circulation*. 1995;92(11):3178–3182.
40. Esquirol Y, et al. Shift work and cardiovascular risk factors: new knowledge from the past decade. *Arch Cardiovasc Dis*. 2011;104(12):636–668.
41. Vyas MV, et al. Shift work and vascular events: systematic review and meta-analysis. *BMJ*. 2012;345:e4800.
42. Bunger MK, et al. Mop3 is an essential component of the master circadian pacemaker in mammals. *Cell*. 2000;103(7):1009–1017.
43. Chen Z, et al. Small molecule modifiers of circadian clocks. *Cell Mol Life Sci*. 2013;70(16):2985–2998.
44. Pan X, et al. Impaired cholesterol metabolism and enhanced atherosclerosis in clock mutant mice. *Circulation*. 2013;128(16):1758–1769.
45. Pan X, et al. Global and hepatocyte-specific ablation of Bmal1 induces hyperlipidaemia and enhances atherosclerosis. *Nat Commun*. 2016;7:13011.
46. Yang G, et al. Timing of expression of the core clock gene Bmal1 influences its effects on aging and survival. *Sci Transl Med*. 2016;8(324):324ra16.
47. Huo M, et al. Myeloid Bmal1 deletion increases monocyte recruitment and worsens atherosclerosis. *FASEB J*. 2017;31(3):1097–1106.

48. Yang G, et al. Bmal1 deletion in myeloid cells attenuates atherosclerotic lesion development and restrains abdominal aortic aneurysm formation in hyperlipidemic mice. *Arterioscler Thromb Vasc Biol*. 2020;40(6):1523–1532.
49. Bass J. Circadian topology of metabolism. *Nature*. 2012;491(7424):348–356.
50. Ramakrishnan SN, Muscat GE. The orphan Rev-erb nuclear receptors: a link between metabolism, circadian rhythm and inflammation? *Nucl Recept Signal*. 2006;4:e009.
51. Kojetin DJ, Burris TP. REV-ERB and ROR nuclear receptors as drug targets. *Nat Rev Drug Discov*. 2014;13(3):197–216.
52. Kuang Z, et al. The intestinal microbiota programs diurnal rhythms in host metabolism through histone deacetylase 3. *Science*. 2019;365(6460):1428–1434.
53. Porsch-Ozcurumez M, et al. The zinc finger protein 202 (ZNF202) is a transcriptional repressor of ATP binding cassette transporter A1 (ABCA1) and ABCG1 gene expression and a modulator of cellular lipid efflux. *J Biol Chem*. 2001;276(15):12427–12433.
54. Probst MC, et al. Screening for functional sequence variations and mutations in ABCA1. *Atherosclerosis*. 2004;175(2):269–279.
55. Schmitz G, Langmann T. Transcriptional regulatory networks in lipid metabolism control ABCA1 expression. *Biochim Biophys Acta*. 2005;1735(1):1–19.
56. Langmann T, et al. ZNF202 is inversely regulated with its target genes ABCA1 and apoE during macrophage differentiation and foam cell formation. *J Lipid Res*. 2003;44(5):968–977.
57. Josekutty J, et al. Microsomal triglyceride transfer protein inhibition induces endoplasmic reticulum stress and increases gene transcription via Ire1α/cJun to enhance plasma ALT/AST. *J Biol Chem*. 2013;288(20):14372–14383.
58. Brown AJ, et al. Cholesterol and oxysterol metabolism and subcellular distribution in macrophage foam cells. Accumulation of oxidized esters in lysosomes. *J Lipid Res*. 2000;41(2):226–237.
59. Carstea ED, et al. Niemann-Pick C1 disease gene: homology to mediators of cholesterol homeostasis. *Science*. 1997;277(5323):228–231.
60. Strauss JF, et al. Sterols and intracellular vesicular trafficking: lessons from the study of NPC1. *Steroids*. 2002;67(12):947–951.
61. Edwards PA, et al. Regulation of gene expression by SREBP and SCAP. *Biochim Biophys Acta*. 2000;1529(1-3):103–113.
62. Wang B, Tontonoz P. Liver X receptors in lipid signalling and membrane homeostasis. *Nat Rev Endocrinol*. 2018;14(8):452–463.
63. Pan X, et al. Nonalcoholic fatty liver disease in CLOCK mutant mice. *J Clin Invest*. 2020;130(8):4282–4300.
64. Pan X, Hussain MM. Bmal1 regulates production of larger lipoproteins by modulating cAMP-responsive element-binding protein H and apolipoprotein AIV. *Hepatology*. 2022;76(1):78–93.
