# Supplementary material for: Bmal1 is involved in the regulation of macrophage cholesterol homeostasis
Source: JCI Insight. 2025 Sep 30;10(21):e194304. doi: 10.1172/jci.insight.194304 (PMC12643489; doi:10.1172/jci.insight.194304)
Supplement: Unedited blot and gel images [file jciinsight-10-194304-s010.pdf]

Figure 2G

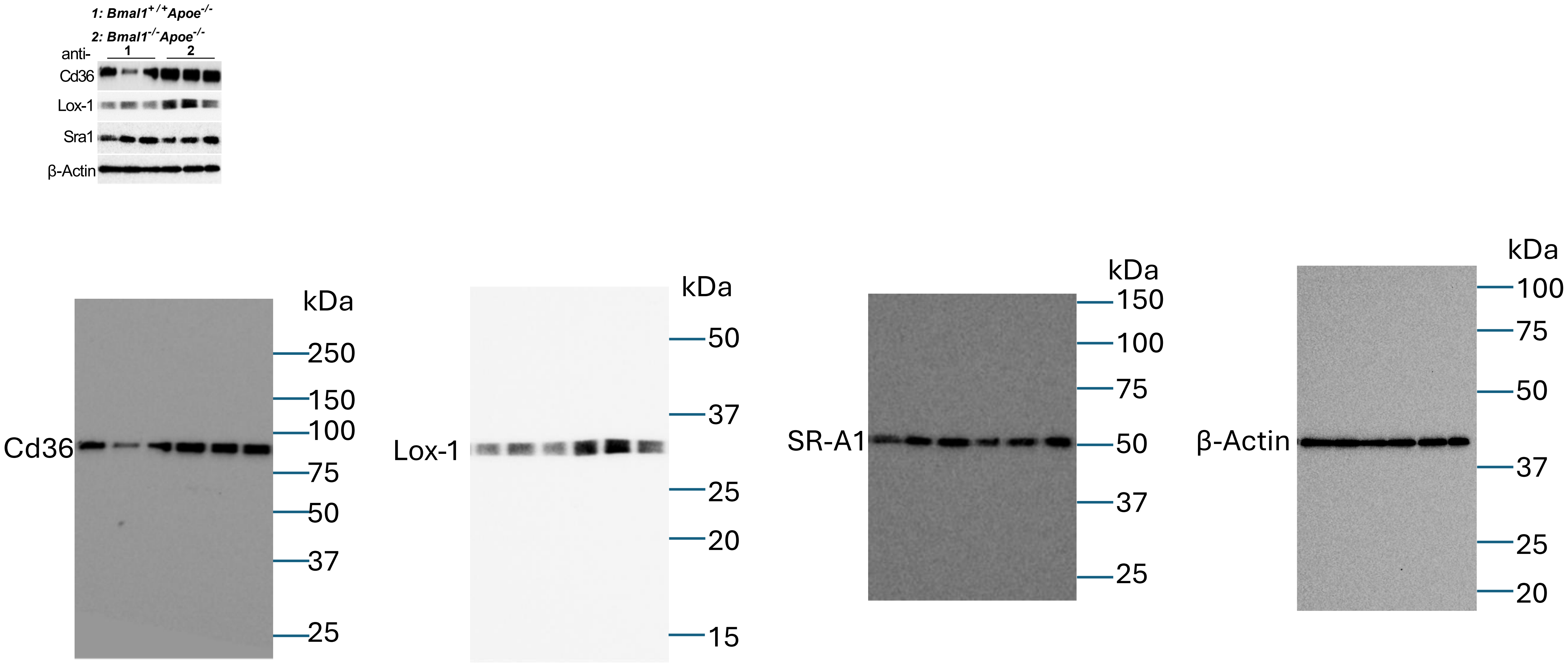
1: Bmal1+/+Apoe-/-
2: Bmal1-/-Apoe-/-
anti-
1
2
Cd36
Lox-1
Sra1
β-Actin
Cd36
kDa
250
150
100
75
50
37
25
Lox-1
kDa
50
37
25
20
15
SR-A1
kDa
150
100
75
50
37
25
β-Actin
kDa
100
75
50
37
25
20

Figure 2G continued

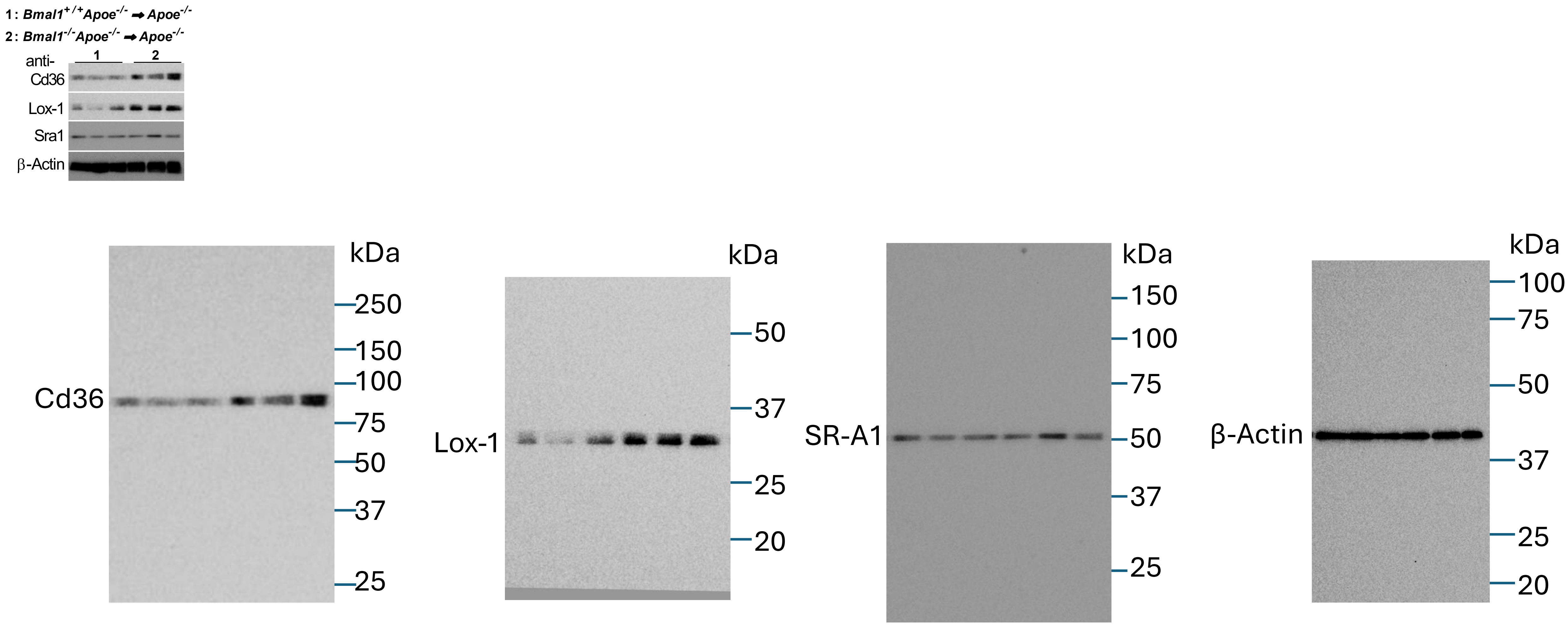

Figure 2H

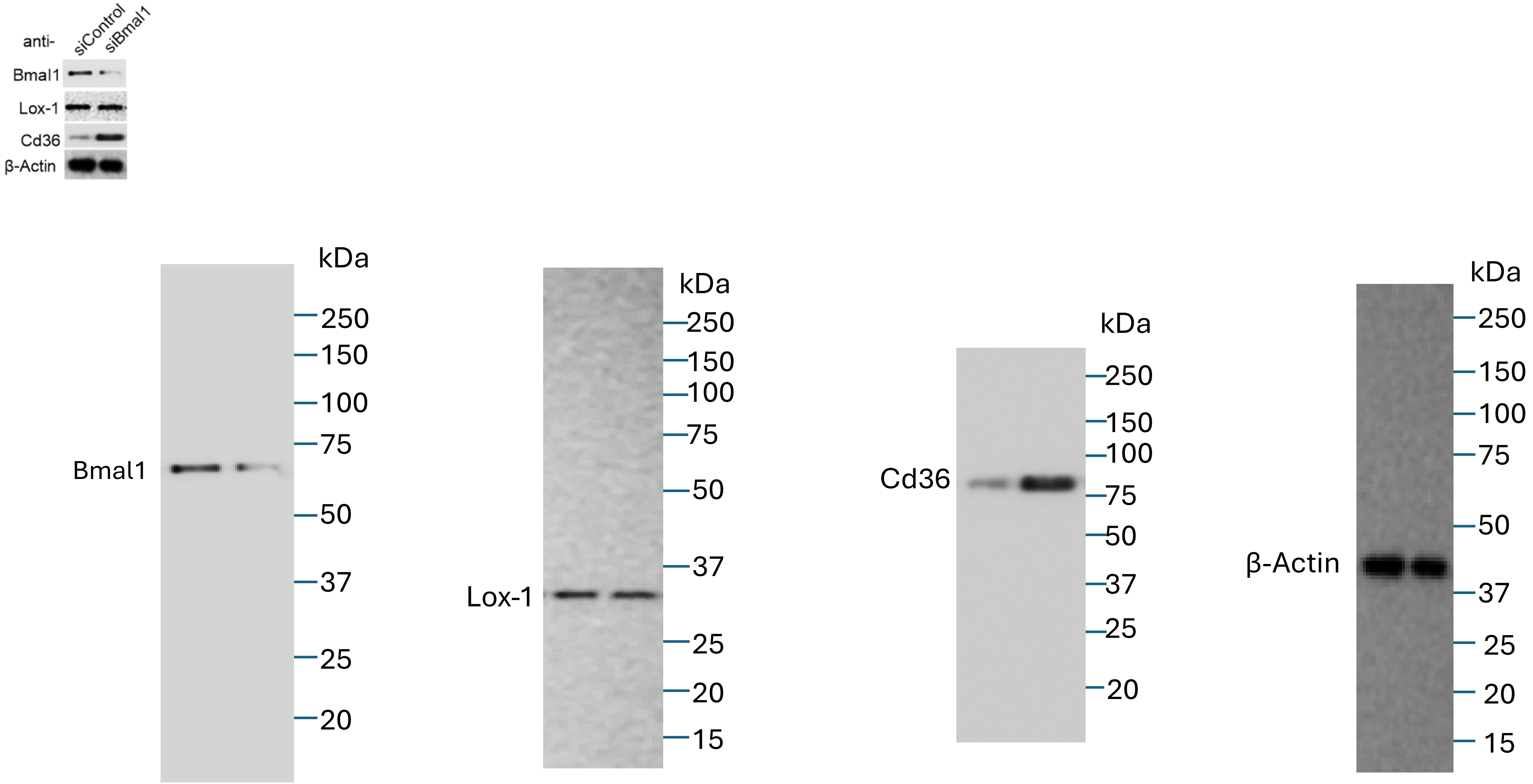

anti-
siControl
siBmal1
Bmal1
Lox-1
Cd36
β-Actin
kDa
250
150
100
75
50
37
25
20
15

Figure 2L

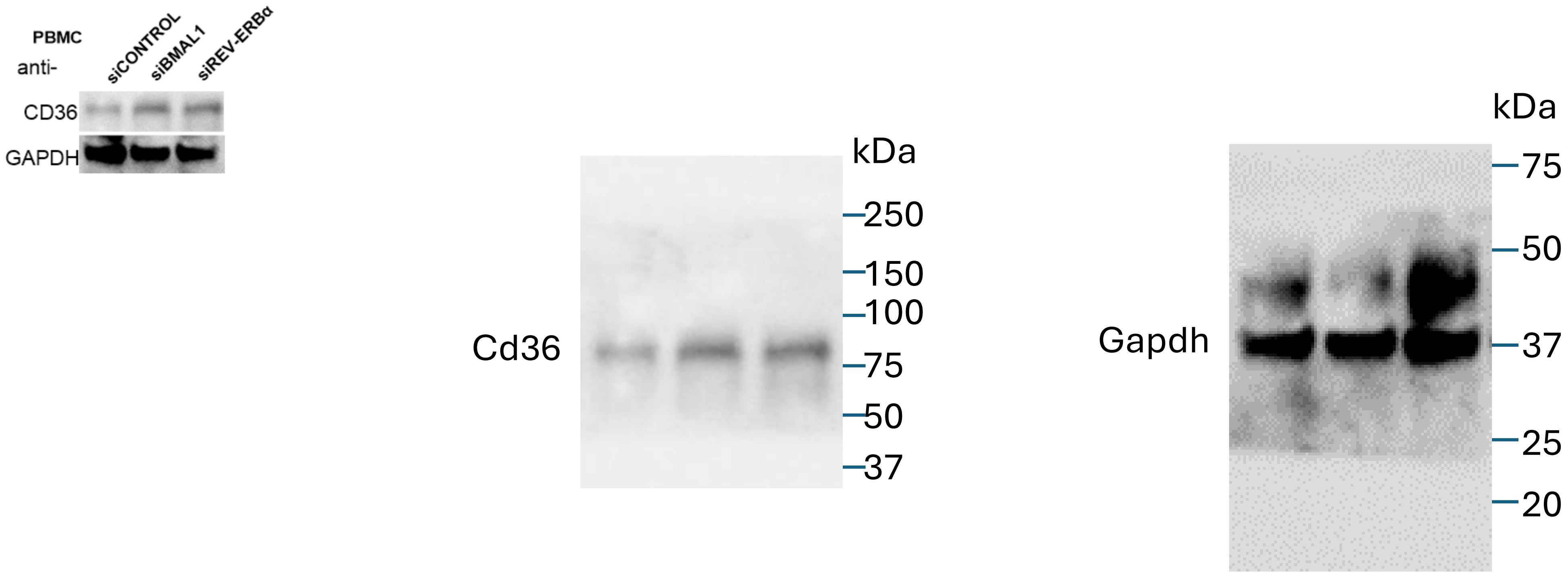
PBMC
anti-
siCONTROL
siBMAL1
siREV-ERBα
CD36
GAPDH
Cd36
kDa
250
150
100
75
50
37
Gapdh
kDa
75
50
37
25
20

Figure 3E

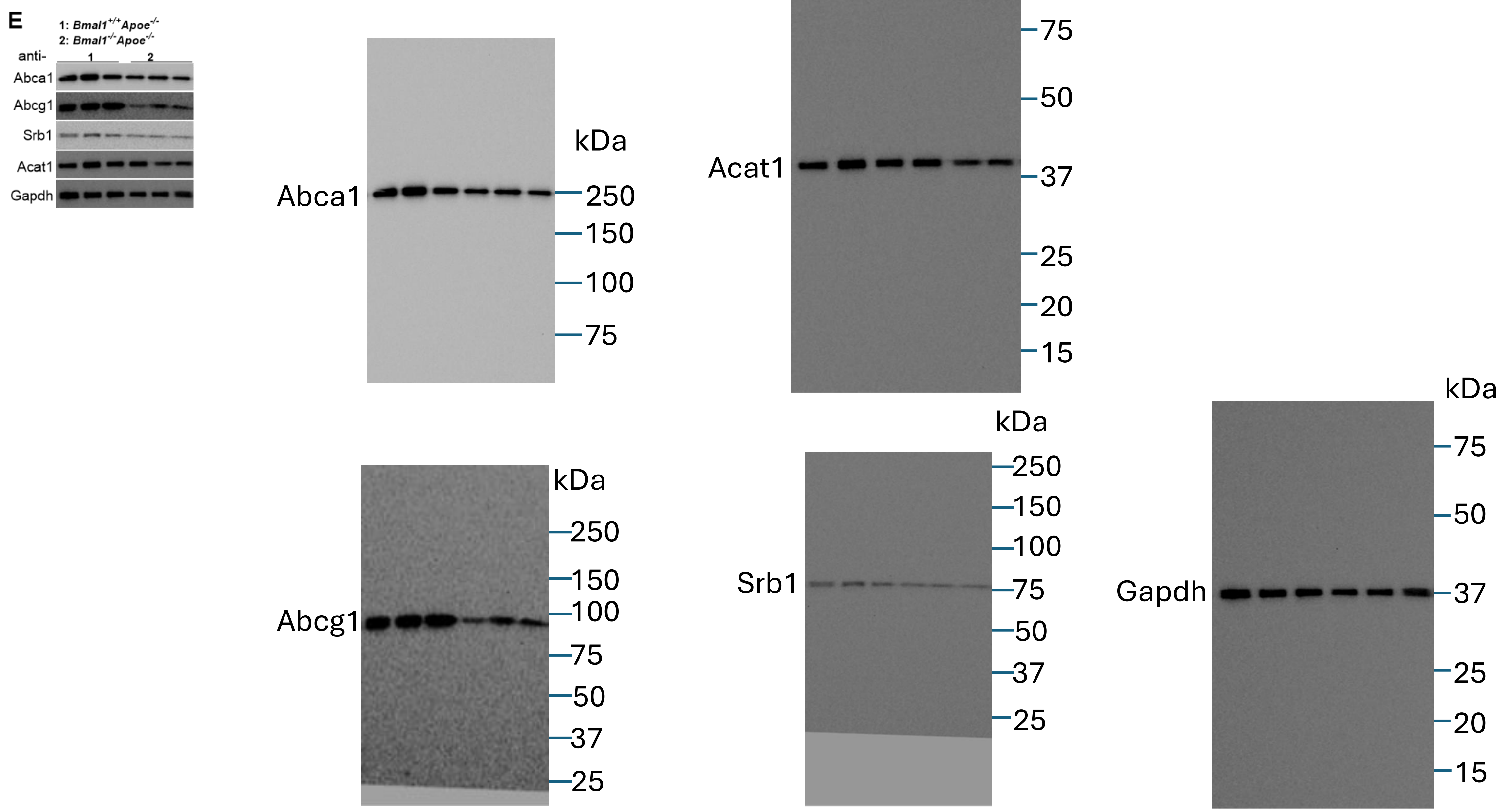
E
1: Bmal1+/+Apoe-/-
2: Bmal1-/-Apoe-/-
anti-
1
2
Abca1
Abcg1
Srb1
Acat1
Gapdh
Abca1
kDa
250
150
100
75
Acat1
kDa
75
50
37
25
20
15
Abcg1
kDa
250
150
100
75
50
37
25
Srb1
kDa
250
150
100
75
50
37
25
Gapdh
kDa
75
50
37
25
20
15

Figure 3E continued

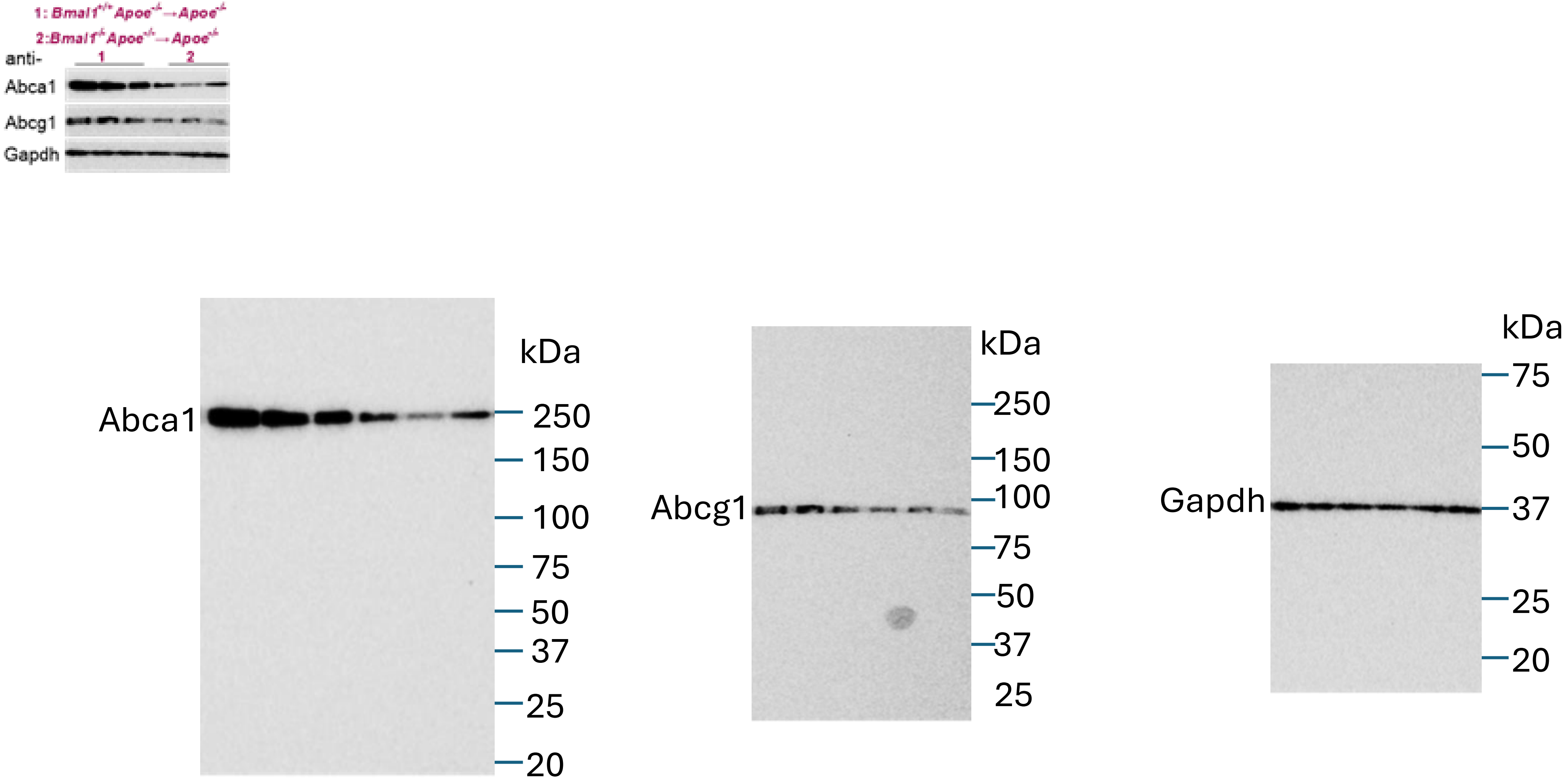

Figure 3E continued

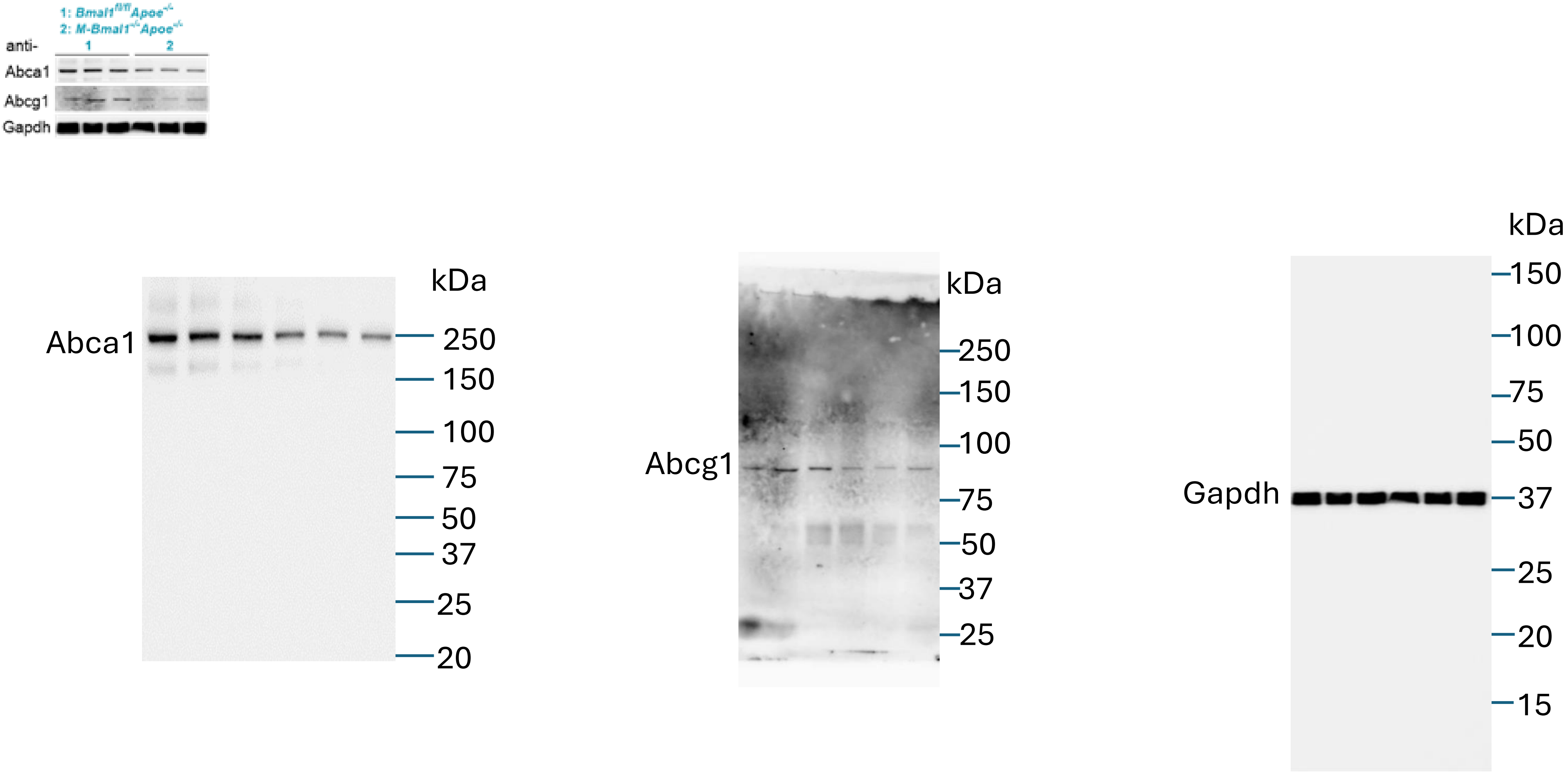

Figure 4B

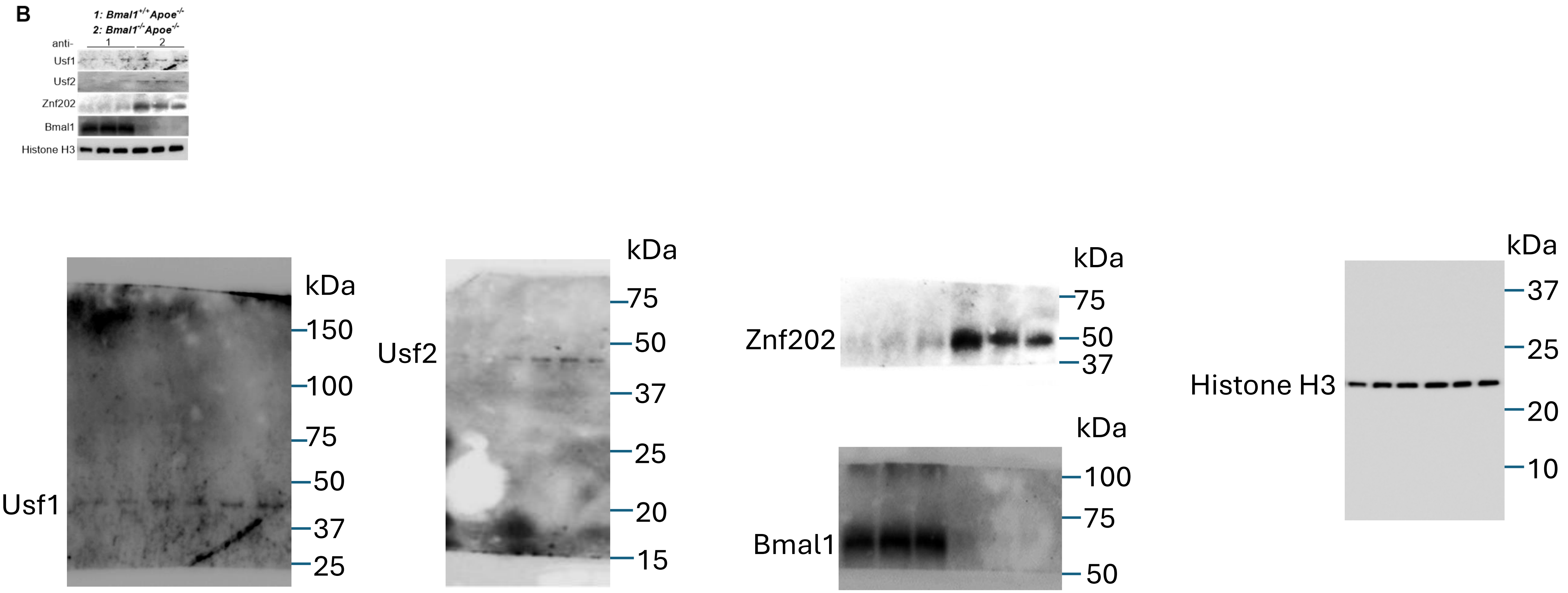
B
1: Bmal1+/+Apoe-/-
2: Bmal1-/-Apoe-/-
anti-
1
2
Usf1
Usf2
Znf202
Bmal1
Histone H3
kDa
150
100
75
50
37
25
Usf1
kDa
75
50
37
25
20
15
Usf2
kDa
75
50
37
Znf202
kDa
100
75
50
Bmal1
kDa
37
25
20
10
Histone H3

Figure 4B continued

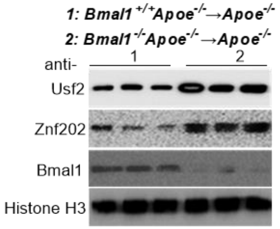

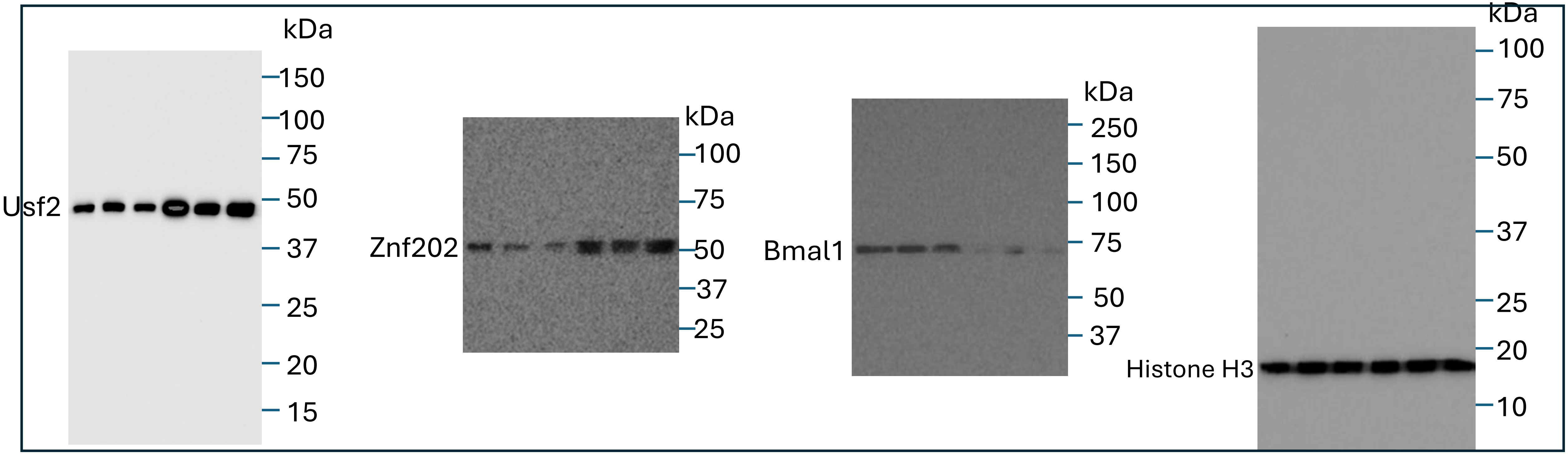

Figure 4B continued

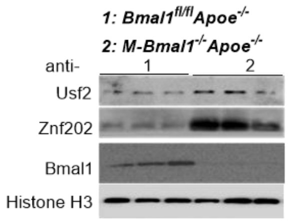

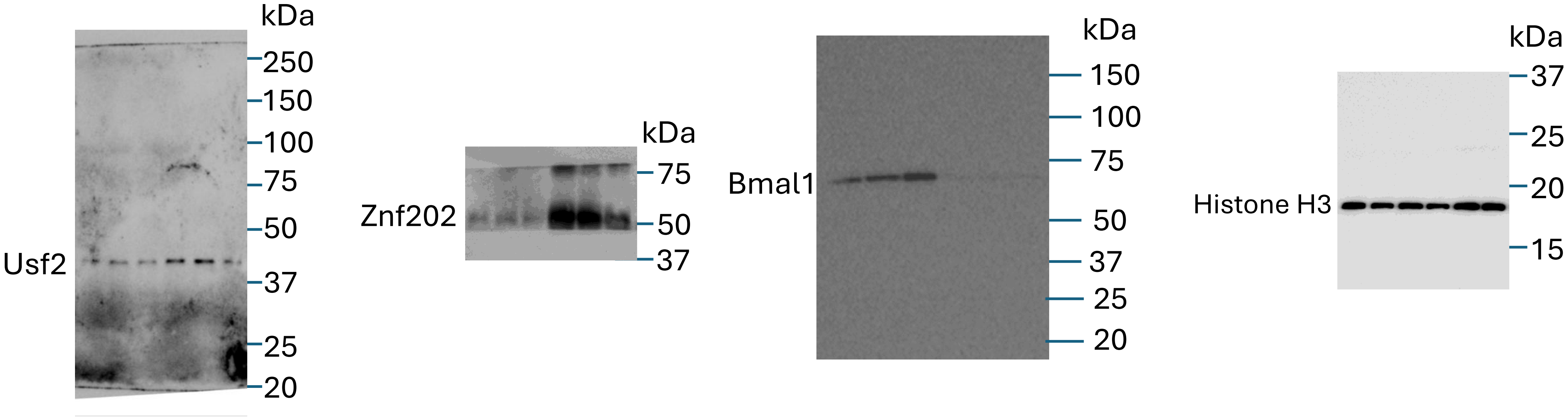

Figure 5C

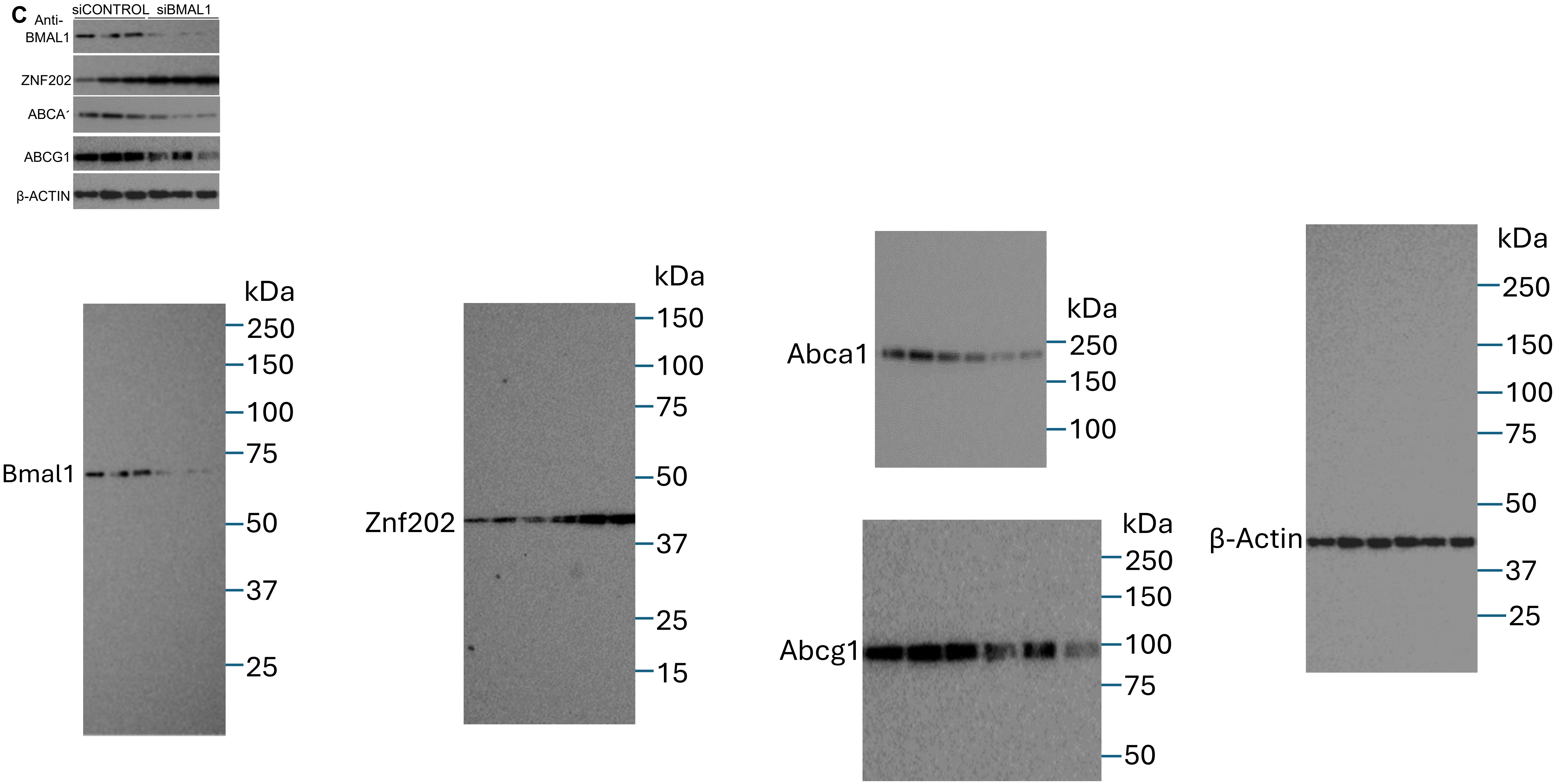
C
Anti-
siCONTROL
siBMAL1
BMAL1
ZNF202
ABCA´
ABCG1
β-ACTIN
Bmal1
kDa
250
150
100
75
50
37
25
Znf202
kDa
150
100
75
50
37
25
15
Abca1
kDa
250
150
100
Abcg1
kDa
250
150
100
75
50
β-Actin
kDa
250
150
100
75
50
37
25

Figure 6G

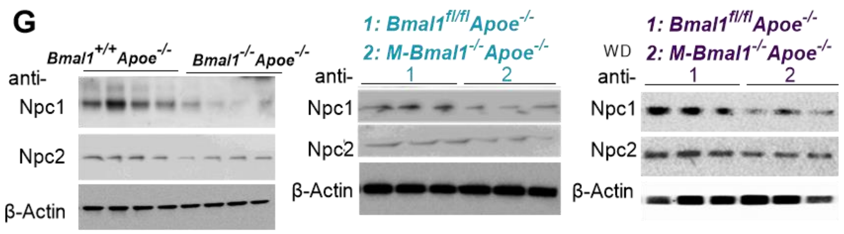
G
Bmal1+/+Apoe-/-
Bmal1-/-Apoe-/-
anti-
Npc1
Npc2
β-Actin
1: Bmal1fl/flApoe-/-
2: M-Bmal1-/-Apoe-/-
anti-
1
2
Npc1
Npc2
β-Actin
1: Bmal1fl/flApoe-/-
WD
2: M-Bmal1-/-Apoe-/-
anti-
1
2
Npc1
Npc2
β-Actin

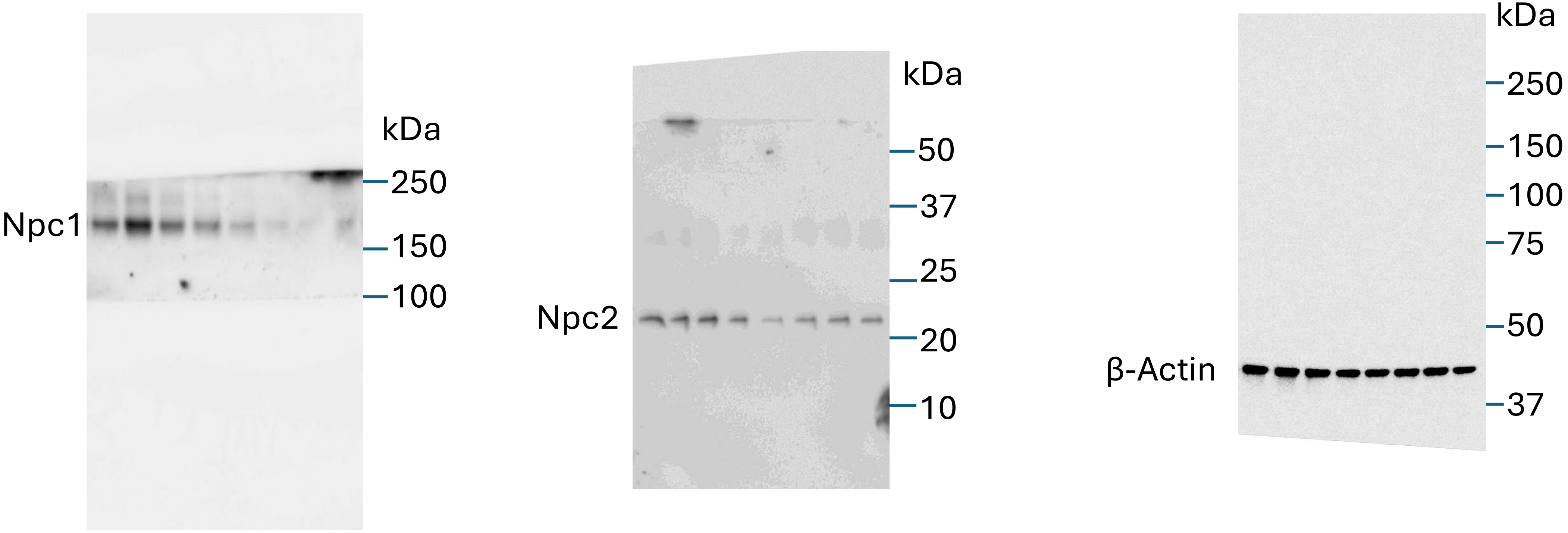
Npc1
kDa
250
150
100
Npc2
kDa
50
37
25
20
10
β-Actin
kDa
250
150
100
75
50
37

Figure 6G continued

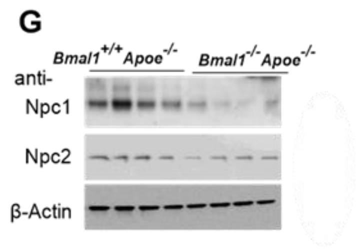

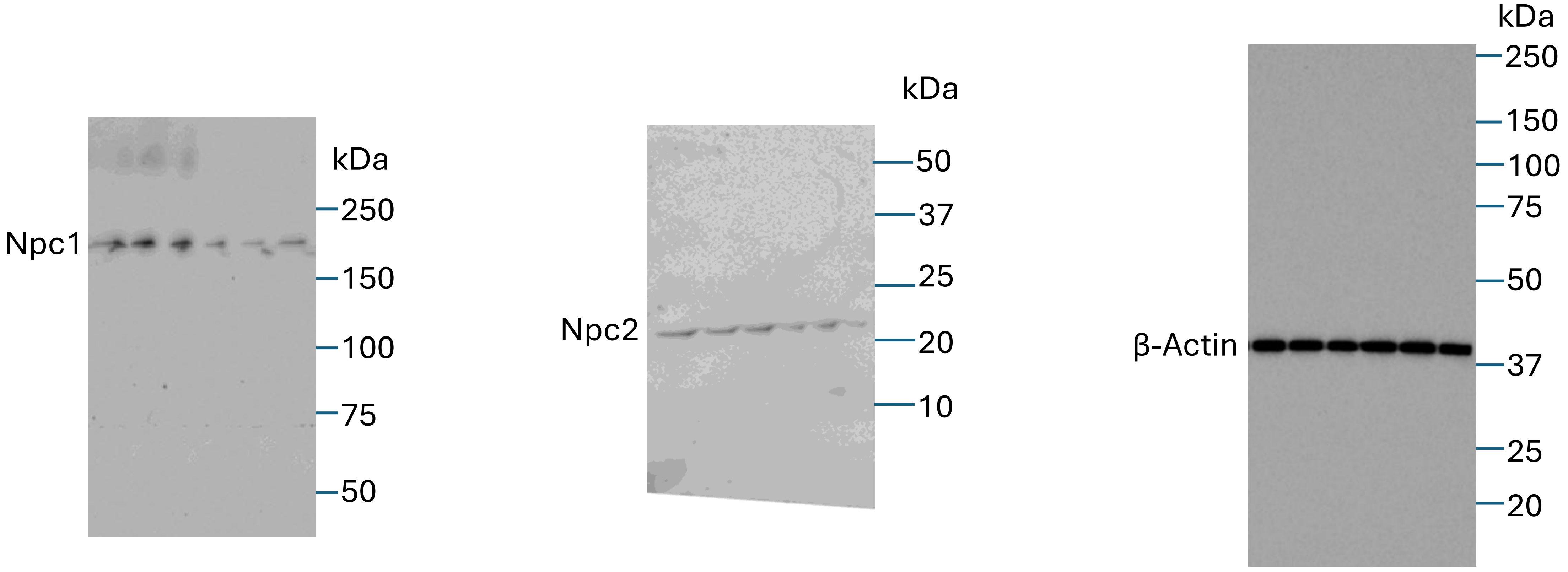

Figure 6G continued

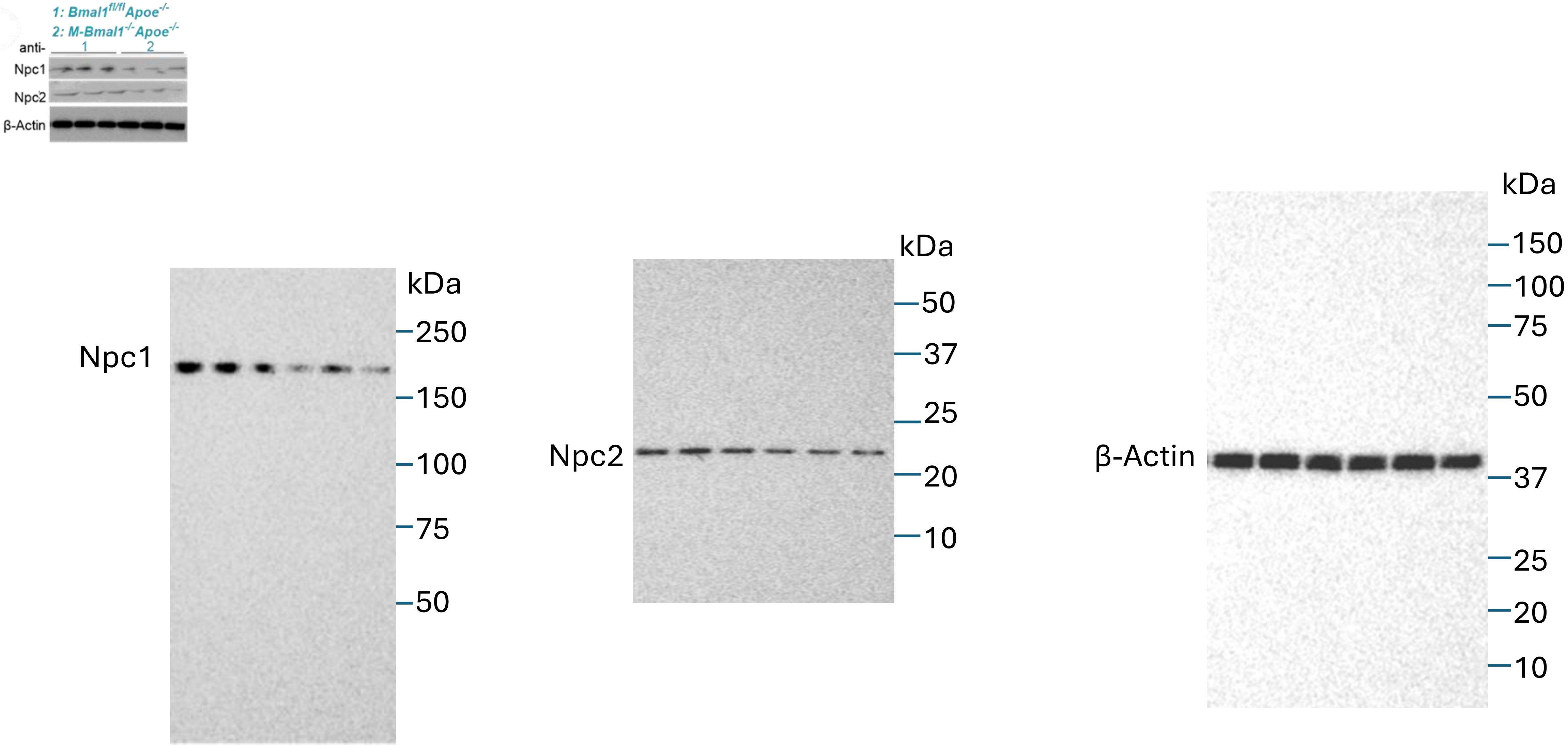

Figure 6H

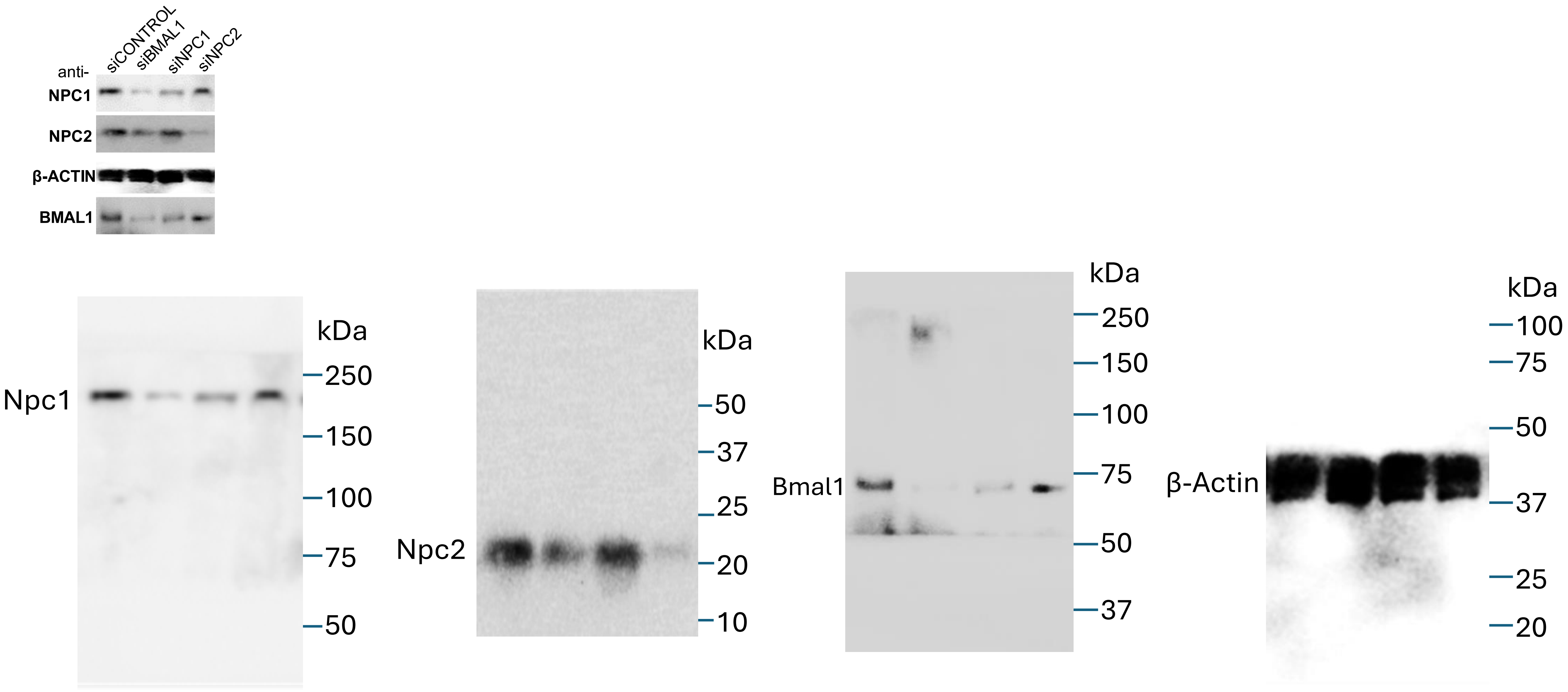
anti-
siCONTROL
siBMAL1
siNPC1
siNPC2
NPC1
NPC2
β-ACTIN
BMAL1
Npc1
kDa
250
150
100
75
50
Npc2
kDa
50
37
25
20
10
Bmal1
kDa
250
150
100
75
50
37
β-Actin
kDa
100
75
50
37
25
20

Figure 7B

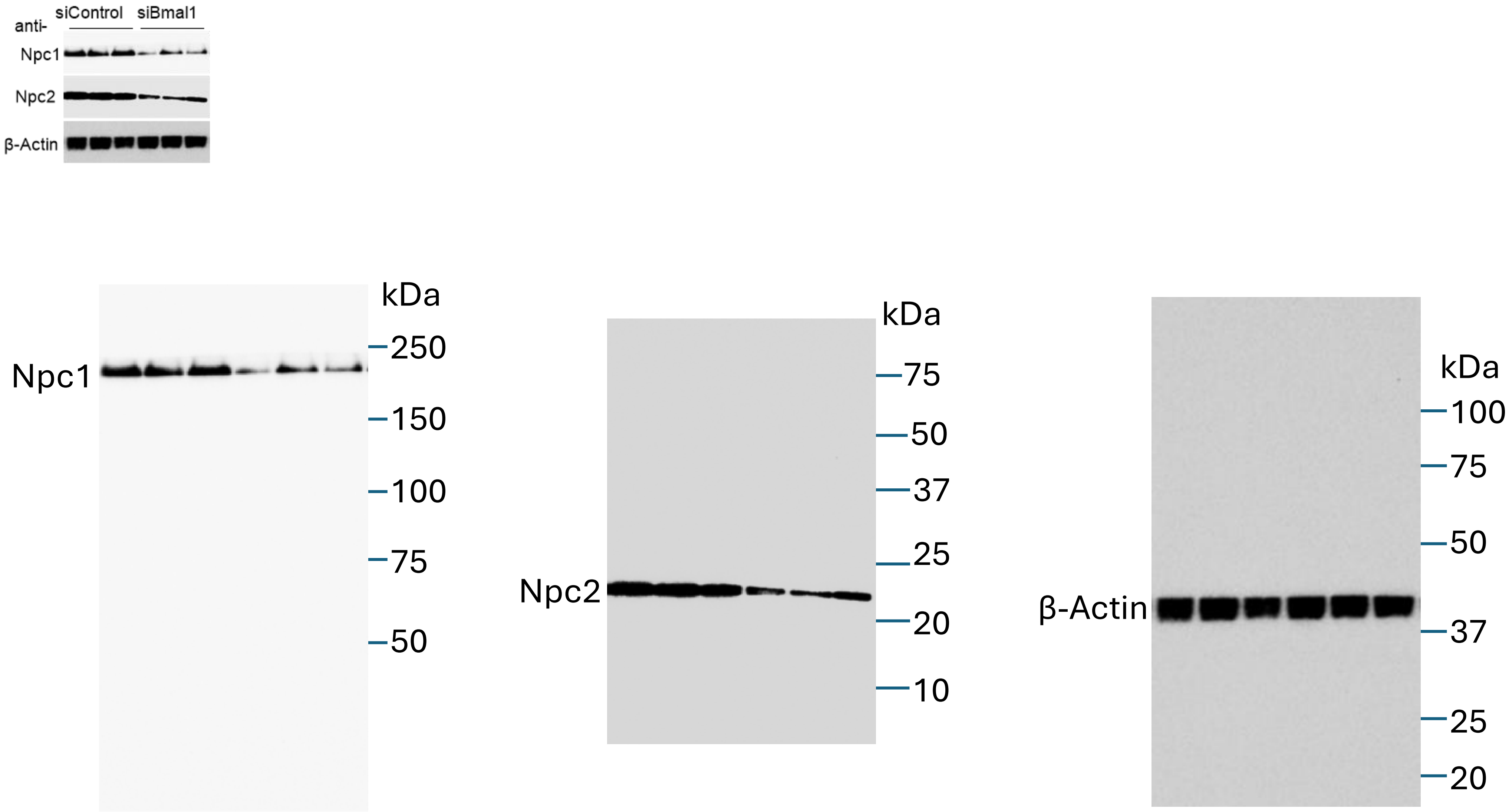
anti-
siControl
siBmal1
Npc1
Npc2
β-Actin
kDa
250
150
100
75
50
Npc1
kDa
75
50
37
25
20
10
Npc2
kDa
100
75
50
37
25
20
β-Actin

Figure 7D

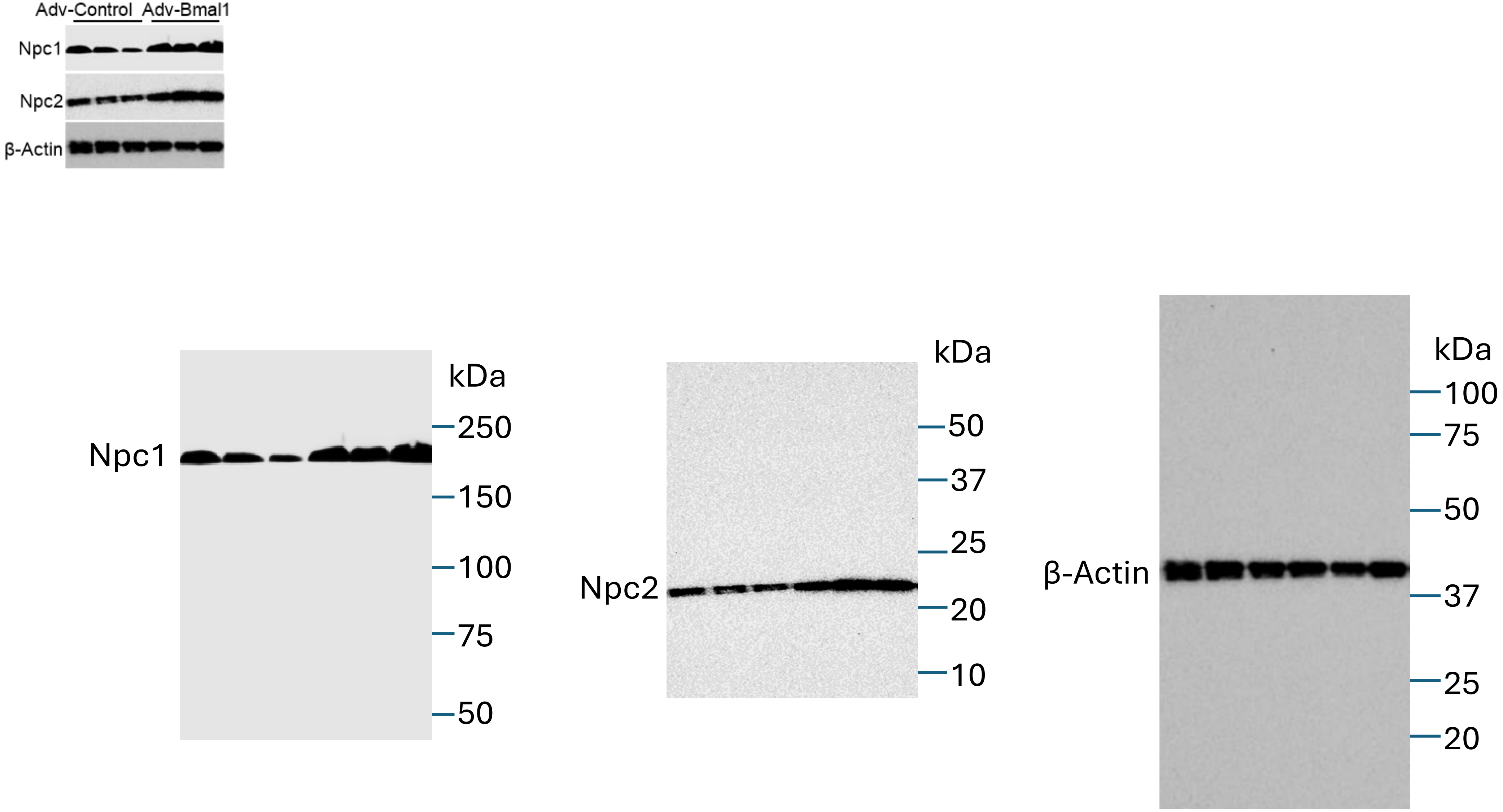

Adv-Control
Adv-Bmal1
Npc1
Npc2
β-Actin
Npc1
kDa
250
150
100
75
50
Npc2
kDa
50
37
25
20
10
β-Actin
kDa
100
75
50
37
25
20

Figure 7G

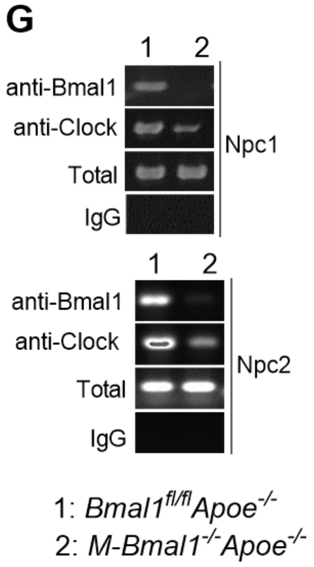
G
1
2
anti-Bmal1
anti-Clock
Total
IgG
Npc1
1
2
anti-Bmal1
anti-Clock
Total
IgG
Npc2
1: Bmal1fl/fl Apoe-/-
2: M-Bmal1-/- Apoe-/-

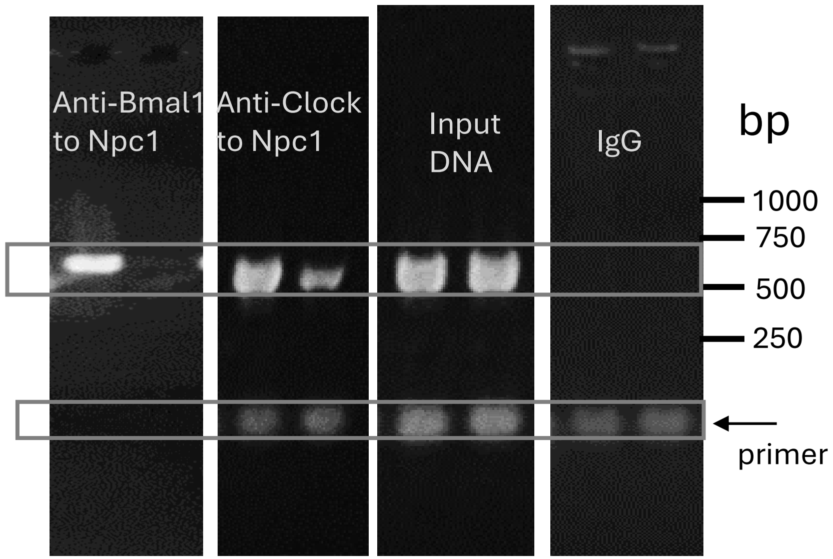
Anti-Bmal1 to Npc1
Anti-Clock to Npc1
Input DNA
IgG
bp
1000
750
500
250
primer

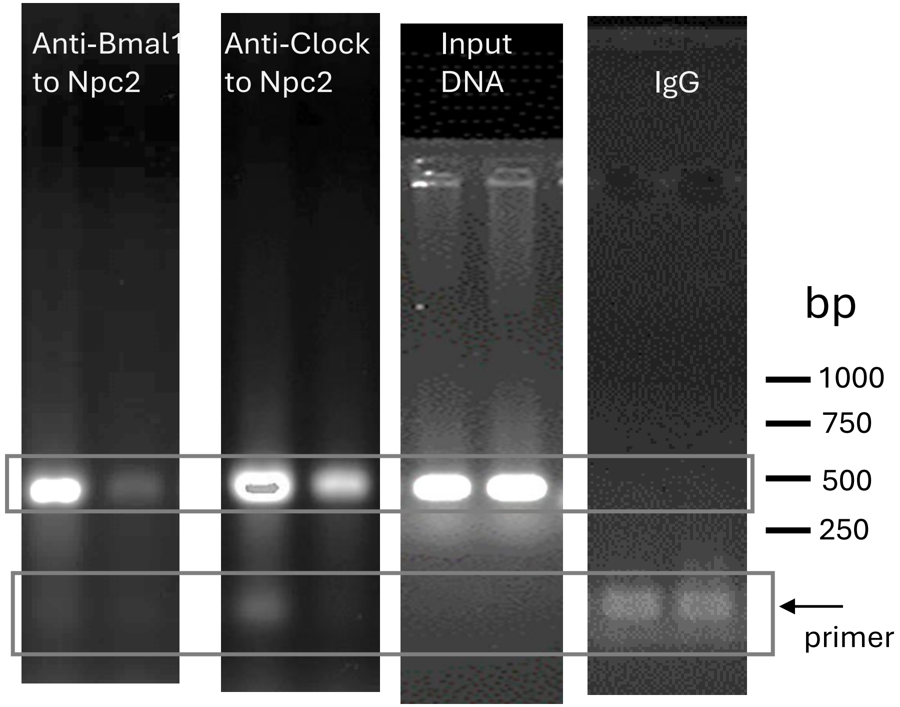
Anti-Bmal1 to Npc2
Anti-Clock to Npc2
Input DNA
IgG
bp
1000
750
500
250
primer

Sup Figure 5E

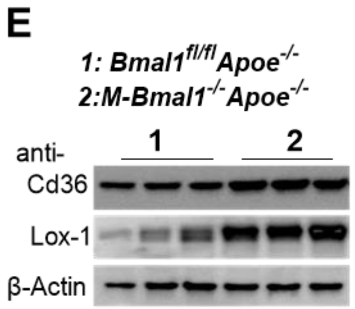

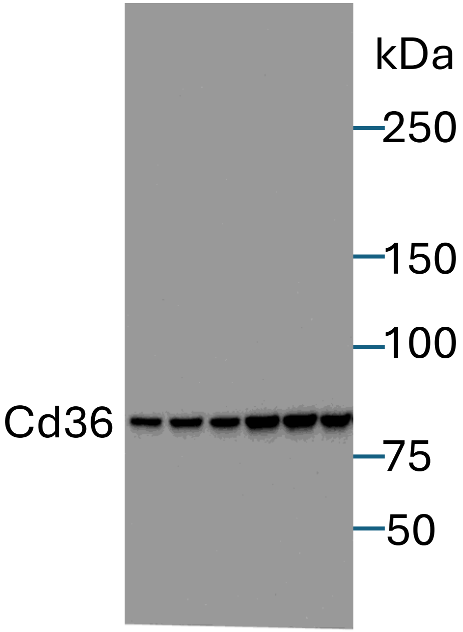

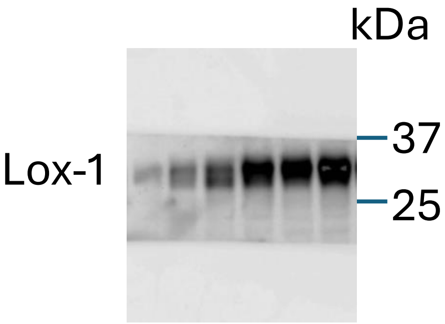

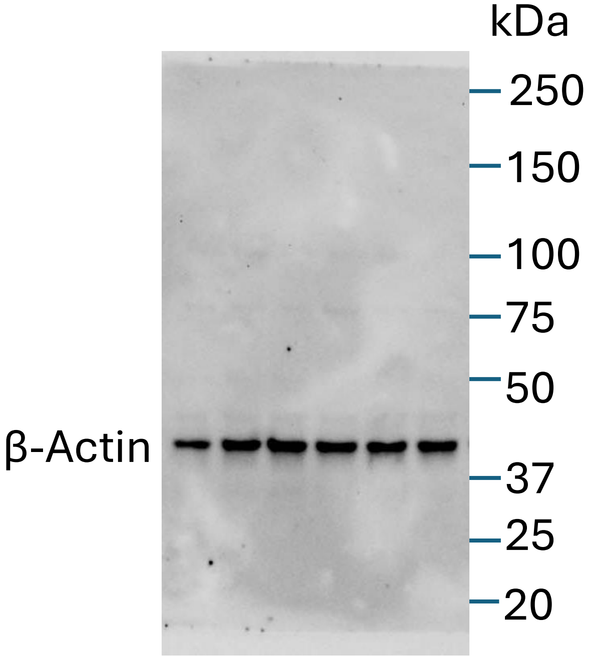

Sup Figure 5E continued

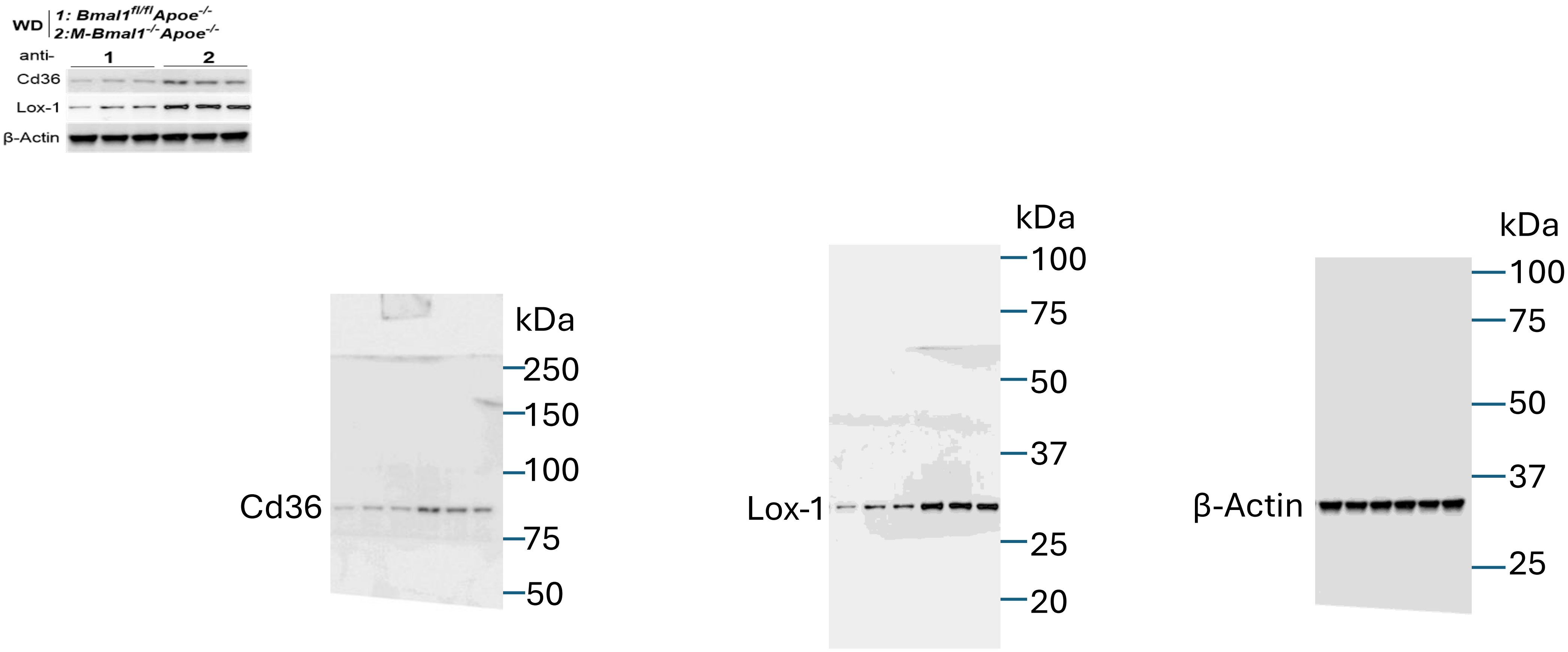

Sup Figure 6

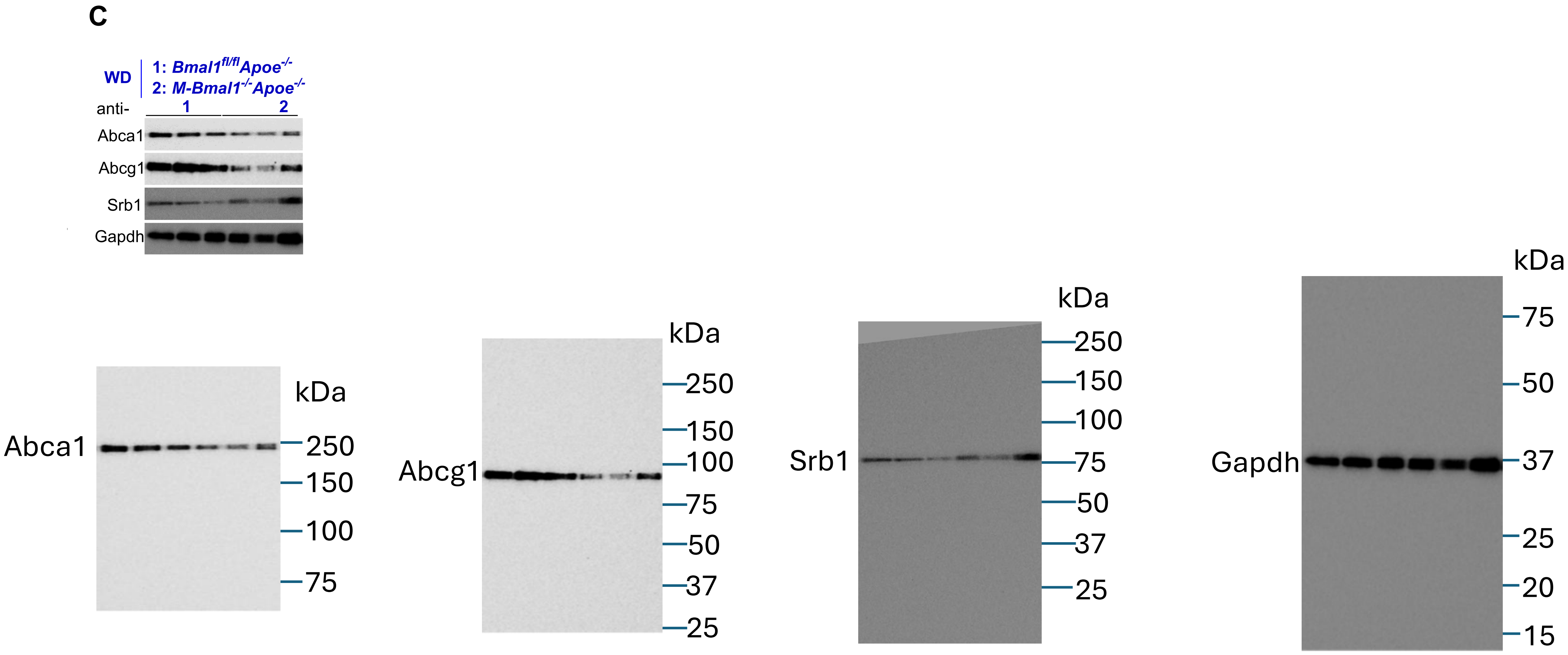
C
WD
1: Bmal1fl/fl Apoe-/-
2: M-Bmal1-/- Apoe-/-
anti-
1
2
Abca1
Abcg1
Srb1
Gapdh
Abca1
kDa
250
150
100
75
Abcg1
kDa
250
150
100
75
50
37
25
Srb1
kDa
250
150
100
75
50
37
25
Gapdh
kDa
75
50
37
25
20
15

Sup Figure 9

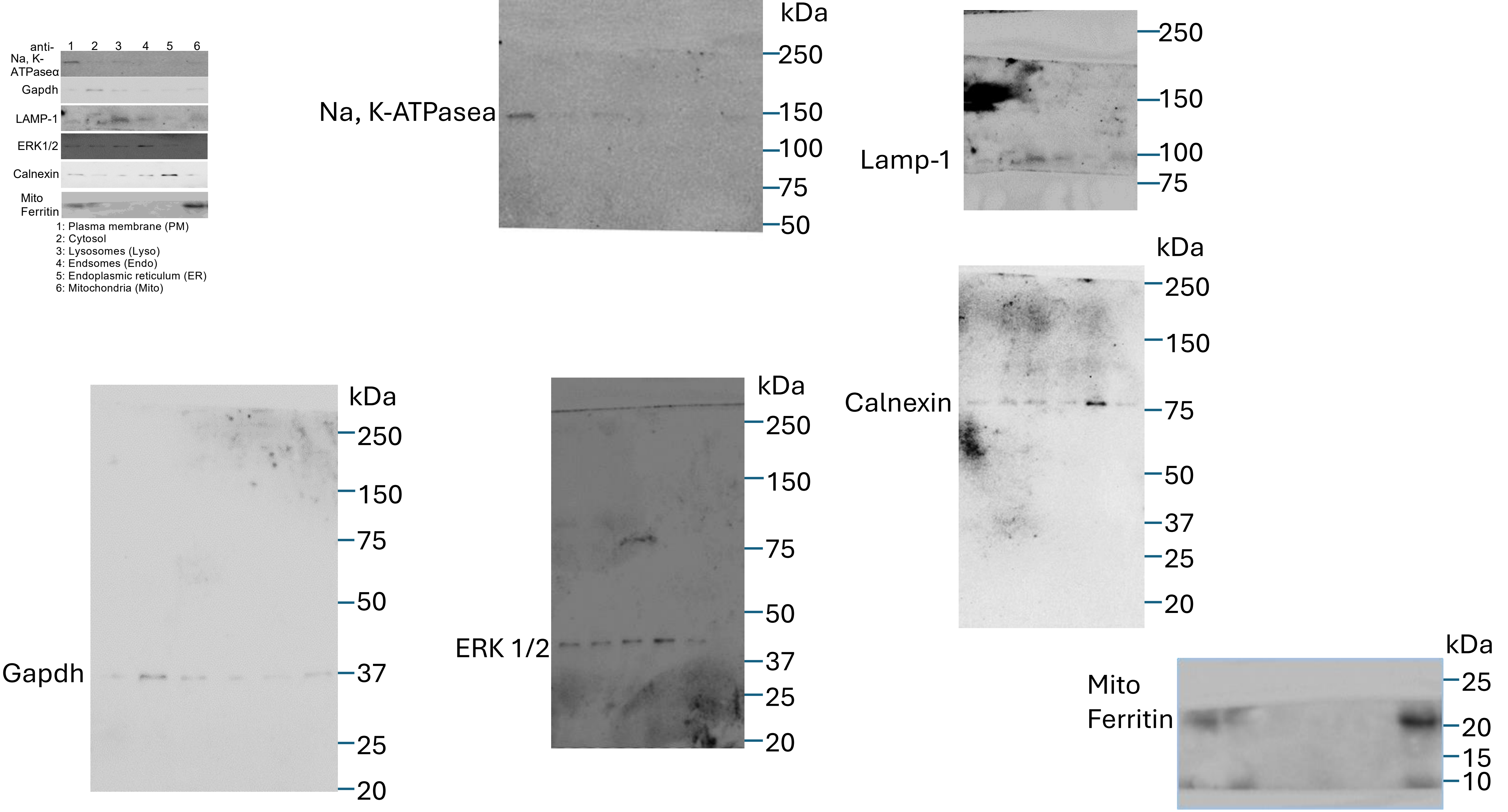
anti-
1 2 3 4 5 6
Na, K-ATPaseα
Gapdh
LAMP-1
ERK1/2
Calnexin
Mito Ferritin
1: Plasma membrane (PM)
2: Cytosol
3: Lysosomes (Lyso)
4: Endsomes (Endo)
5: Endoplasmic reticulum (ER)
6: Mitochondria (Mito)
Na, K-ATPasea
kDa
250
150
100
75
50
Lamp-1
kDa
250
150
100
75
Calnexin
kDa
250
150
75
50
37
25
20
Gapdh
kDa
250
150
75
50
37
25
20
ERK 1/2
kDa
250
150
75
50
37
25
20
Mito Ferritin
kDa
25
20
15
10
